# Discovery of new muscarinic acetylcholine receptor antagonists from *Scopolia tangutica*

**DOI:** 10.1038/srep46067

**Published:** 2017-04-07

**Authors:** Nana Du, Yanfang Liu, Xiuli Zhang, Jixia Wang, Jianqiang Zhao, Jian He, Han Zhou, Lijuan Mei, Xinmiao Liang

**Affiliations:** 1 Key Lab of Separation Science for Analytical Chemistry, Dalian Institute of Chemical Physics, Chinese Academy of Sciences, Dalian, China; 2 University of Chinese Academy of Sciences, Beijing, China; 3 Key Lab of Tibetan Medicine Research, Northwest Institute of Plateau Biology, Chinese Academy of Sciences, Xining 810008, China; 4 Co-innovation Center of Neuroregeneration, Nantong University, Nantong, 226019, China

## Abstract

*Scopolia tangutica (S. tangutica*) is a traditional Chinese medicinal plant used for antispasmodics, anesthesia, analgesia and sedation. Its pharmacological activities are mostly associated with the antagonistic activity at muscarinic acetylcholine receptors (mAchRs) of several known alkaloids such as atropine and scopolamine. With our recent identification of four hydroxycinnamic acid amides from *S. tangutica*, we hypothesized that this plant may contain previously unidentified alkaloids that may also contribute to its *in vivo* effect. Herein, we used a bioassay-guided multi-dimension separation strategy to discover novel mAchR antagonists from *S. tangutica*. The core of this approach is to use label-free cell phenotypic assay to first identify active fractions, and then to guide purification of active ligands. Besides four tropanes and six cinnamic acid amides that have been previously isolated from *S. tangutica*, we recently identified two new tropanes, one new cinnamic acid amide, and nine other compounds. Six tropane compounds purified from *S. tangutica* for the first time were confirmed to be competitive antagonists of muscarinic receptor 3 (M3), including the two new ones **8** and **12** with IC_50_ values of 1.97 μM and 4.47 μM, respectively. Furthermore, the cinnamic acid amide **17** displayed 15-fold selectivity for M1 over M3 receptors. These findings will be useful in designing lead compounds for mAchRs and elucidating mechanisms of action of *S. tangutica*.

Natural products are a rich source of lead compounds for drug discovery and development^1^,^2^. *Scopolia tangutica (S. tangutica*) is a traditional Chinese medicinal (TCM) plant found in Tibet, Yunnan, Sichuan, Gansu, and Qinghai Provinces, and has been used for antispasmodics, anesthesia, analgesia and sedation for many years in China. Its pharmacological activities are mostly associated with several known tropane alkaloids including scopolamine and atropine that are highly potent antagonists of muscarinic acetylcholine receptors (mAchRs)^3^,^4^,^5^. mAchRs are a family of five G protein-coupled receptors (GPCRs), among which M1, M2 and M3 receptors are proven drug targets. mAchRs are found in periphery system and central nervous system, and related to diseases such as asthma, Alzheimer’s disease, memory impairment, chronic obstructive pulmonary disease, Parkinson’s disease, and depression^6^,^7^,^8^,^9^,^10^,^11^,^12^. Tropane alkaloids contain an octane framework and several chiral centers^13^, which often make medicinal chemistry optimization difficult. Thus, identification of new alkaloids from *S. tangutica* may provide a possibility to discover novel lead-like ligands for mAchRs.

We speculated, based on two observations, that *S. tangutica* may contain new alkaloids contributing to its *in vivo* pharmacology. First, traditional approaches such as solvent extraction followed by thin-layer chromatography^14^,^15^ or one-dimensional (1D) high performance liquid chromatography-mass spectrometry (HPLC-MS)^16^,^17^,^18^ often result in isolation and identification of small sets of compounds because of the chemical diversity of many medicinal plants. Second, our recent non-aqueous solid phase extraction (SPE)^19^ and 2D-HPLC methods^20^ have resulted in the identification of four hydroxycinnamic acid amides from *S. tangutica* for the first time^21^. This study also suggested the presence of a large number of minor alkaloids. Since a large quantity of plants is required to obtain a sufficient amount of compounds from these minor alkaloids for pharmacology profiling, non-targeted isolation will be a laborious and time-consuming work. Therefore, activity-guided preparation is an ideal method to accelerate the discovery of novel lead-like compounds^1^,^22^. The main idea of the strategy is to apply label-free cell phenotypic assay afforded by resonant waveguide grating (RWG) biosensor to first identify active fractions, and then to guide the purification of active compounds. Surface bound evanescent waves and tunable light source provided by the label-free screening device, RWG biochemical assay characterizes the process of dynamic mass redistribution (DMR) caused by probes interaction through refractive index variations^23^. The 384-well biosensor assay permits a holistic, pathway sensitive readout of receptor pharmacology with high throughput^24^,^25^,^26^. The noninvasive and holistic measurement of the label-free technique enables multiple assay formats to identify and elucidate the pharmacology of hit ligands or multiple targets all within a single screening campaign, especially for GPCRs^27^,^28^.

Herein, we applied the label-free cell phenotypic assay-guided preparation strategy to discover minor active alkaloids from *S. tangutica*. This workflow assisted us to isolate a series of pure compounds and identify their antagonistic activities on the endogenous M3 receptor in HT-29 cells^29^.

## Results

### Identification of active fractions of alkaloid constituents from *S. tangutica*

**Figure 1:**
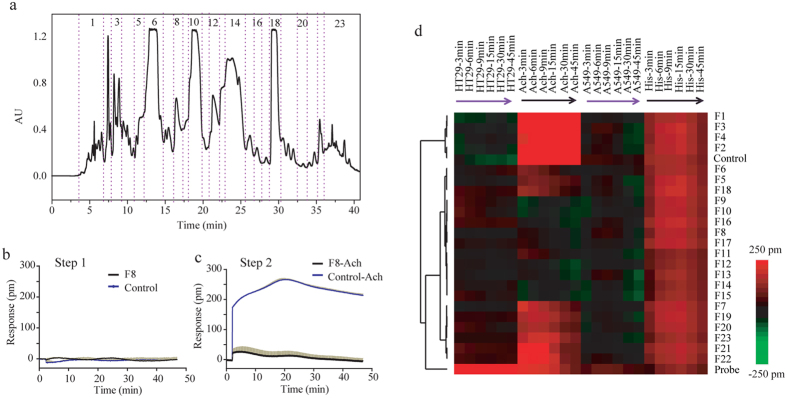
Label-free cell phenotypic profiling guided compound preparation and identification. (**a**) Chromatography of the first dimensional preparation and fraction collection. (**b**) Representative dynamic mass redistribution (DMR) traces of fraction 8 (F8) and buffer (control) in HT-29 cells (pm represented picometer, shift in resonant wavelength of the biosensor after poststimulation by fraction) (**c**) The DMR traces of 16 μM acetylcholine after the pretreatment with F8 or buffer for 1 hr. DMR traces in (**b,c**) represent the mean ± s.d. (n = 4). (**d**) DMR heat map of 23 fractions and probes in HT-29 and A549 cell lines. The heat map was obtained by cluster analysis of the DMR profiles of the 23 fractions in both cell lines. For each fraction, real responses of both the fraction and the probe after the fraction pretreatment, each at six discrete time points post-stimulation (3, 6, 9, 15, 30, 45 min), were used for the cluster analysis. All fractions were assayed at 1.25 mg/L. The probe was acetylcholine (Ach) for M3 receptor in HT-29, and histamine (His) for histamine receptors in A549. The control was buffer. Color code is green, negative; red, positive; and black, zero response.

To perform the activity-guided purification, the alkaloids enriched from *S. tangutica* using the SPE method^19^ were the first subject to separation on an XCharge C18 column. Results showed that the enriched alkaloids gave rises to a series of well separated and symmetric peaks even at an overloading amount on the column (Fig. 1a). Twenty-three fractions (F1 to F23) were collected sequentially according to visible peaks and these fractions have little peak overlapping (Fig. S1).

Given that *S. tangutica* is used to treat spasm and asthma, we screened these fractions on M3 receptor in HT-29 due to its high expression of M3 receptor endogenously and robust DMR signals after treatment with agonist^29^. The screening was performed via a two-step assay, of which the first step was to examine the agonistic activity of each fraction, and the second step to examine the ability of each fraction to block the DMR signal arising from the activation of M3. For instance, F8 triggers little DMR signal in HT-29 cells, similar to the control signals (Fig. 1b). However, the fraction almost completely blocks the DMR of 16 μM acetylcholine, a non-selective agonist for muscarinic receptors (Fig. 1c), suggesting that F8 contains at least one M3 antagonist.

To illustrate the effect of all fractions in both cell lines, we produced a heat map of all fractions based on cluster analysis of all DMR responses obtained (Fig. 1d). Results show that F8 to F17 induce no clear DMR signals in HT-29, but have obvious inhibitory effects on the acetylcholine DMR, while F5, F6, F7 and F18 show partial inhibition. Histamine receptor (H receptor), another receptor also related to asthma, was also tested and A549 cell line was preferred for its endogenous expression^30^ of H receptor, based on the fast proliferation and well adhering property of this cell line. As a result, nearly all fractions have little effect on the histamine DMR in A549. It suggests that these fractions F5 to F18 may contain M3 antagonists. Thus, these active fractions were chosen to purify compounds for investigating new M3 antagonists.

### Purification of alkaloid compounds from active fractions of *S. tangutica*

**Figure 2:**
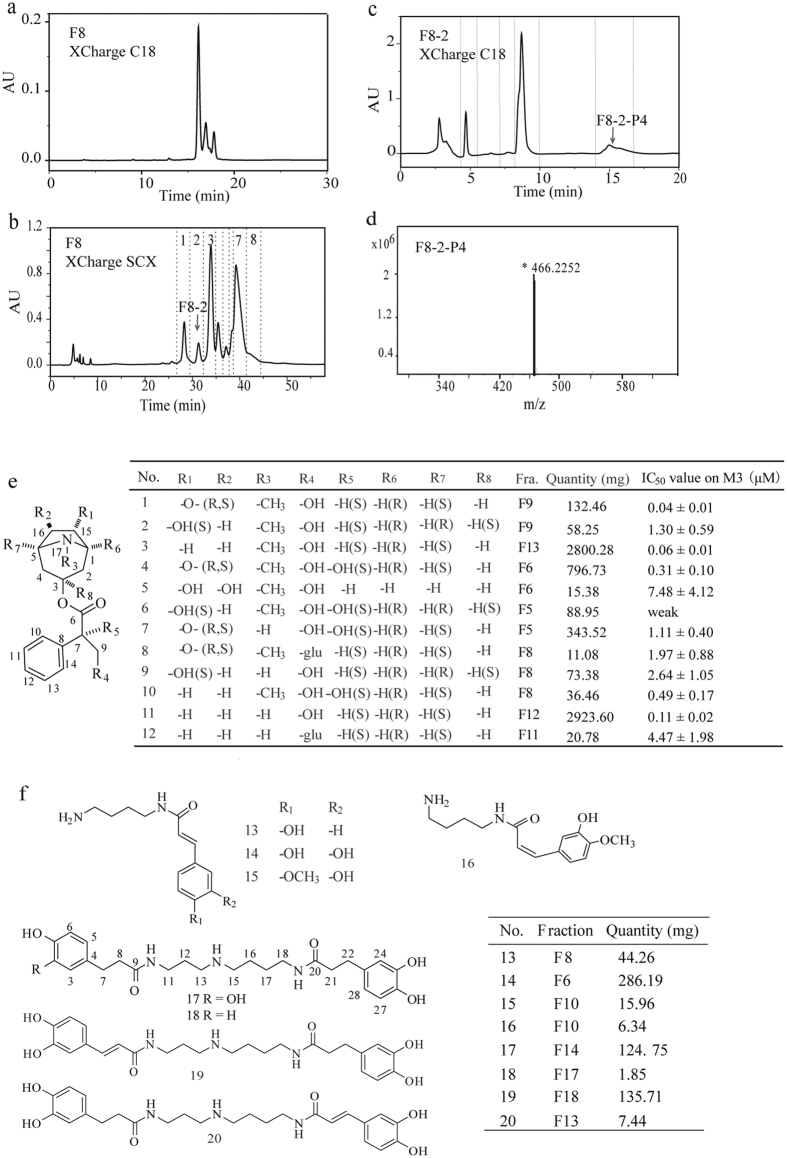
Purification of compounds from *S. tangutica*. (**a**) Chromatography of F8 on an XCharge C18 column in the first dimensional separation (1D) method. (**b**) Chromatography of F8 on XCharge SCX column in the second dimension. (**c**) Chromatography of F8-2 on an XCharge C18 column in the third dimension. (**a**–**c**) were all detected in a wavelength of 210 nm. (**d**) MS spectrum of F8-2-P4 (**8**) at a positive ESI mode. (**e**) Twelve tropane compounds isolated from *S. tangutica*. -O- is epoxide; -O- (R,S) means that R_1_-linked carbon in R-configuration and R_2_-linked carbon in S-configuration; -OH(S) means that hydroxyl-linked carbon in S-configuration. (**f**) Eight cinnamic acid amides isolated from *S. tangutica.*

We employed multi-dimension HPLC to purify and identify active compound(s) from each active fraction. First, XCharge SCX (SCX) column was used as the second dimensional liquid chromatography, due to the difference in separation selectivity from the first dimension. For instance, compared to the XCharge C18 column in the first dimension separation (Fig. 2a), F8 exhibited different chromatography behavior on the SCX column, leading to further fine separation of this fraction (Fig. 2b). Thus, we performed systematical preparation of all active fractions (F5 to F18) and obtained 111 secondary fractions in total using the SCX column. We further performed purity analyses of all these secondary fractions on both XCharge C18 and SCX columns. Results showed that out of the 111 fractions, only five were pure.

Second, we performed the third dimensional separation to obtain pure compounds from the fractions. An XCharge C18 column in a different mobile phase system from the first dimensional separation was used to realize further purification and desalting process synchronously. In the third dimension, peaks were systematically collected, including minor peaks like F8-2-P4, the fourth peak of F8-2 (Fig. 2c), the purity of which was confirmed by MS (Fig. 2d). As a result, we obtained 255 final fractions in total. Purity analyses show that 136 fractions have purity higher than 90%. For the remaining 119 fractions, their insufficient quantities make it difficult to further purify compounds of high structural similarity, including homologues and isomers, such as F16-7-P6 (Fig. S3) and F16-7-P7 (Fig. S4). Furthermore, among the pure fractions, duplications between adjacent fractions result in repeated isolation of the same compound, such as the compound norhyoscyamine that was obtained as a secondary fraction F12-8 and a final fraction F12-10-P1.

Third, MS and Nuclear Magnetic Resonance (NMR) were used to determine chemical structures of active compounds. Two new tropane compounds and one cinnamic acid amide were structurally characterized in detail and known compounds were elucidated in Supporting information. Compound **8** was a new monomer with a formula of C_23_H_31_NO_9_. The predominant fragment ions were 466.2079 and 304.1550, which showed the character of neutral loss of 162 Da (one glycocyl group). The carbon at the chemical shift of 102.93 and its corresponding proton at 4.43 in HSQC spectrum indicated the presence of glucose. The coupling constant (*J* = 8.0 Hz) between 4.43 (1′-H) and 3.14 (2′-H) confirmed the configuration of glucose as β-D-glucose. Chemical shifts of protons in ^1^H NMR spectrum except hydrogen linked to β-D-glucose were in consistent with that of scopolamine in same configuration and additionally, mass fragment 304.1550 for [M+H]^+^ of **8** and ultraviolet spectrum uniformity further confirmed the structure as a combination of scopolamine and β-D-glucose. Coupling between 102.93 (C-1′) and 4.22, 4.14 (9-H) in HMBC and coupling between 4.43 (1′-H) and 4.22, 4.14 (9-H) in NOESY demonstrated β-D-glucose was linked at C-9 of **8**. Therefore, this compound was identified to be glycoscopolamine.

Compound **12** was another new tropane alkaloid with a formula of C_22_H_31_NO_8_. Based on the same reasoning as **8**, a neutral loss of 162 Da (one glycocyl group), ^13^C and ^1^H NMR and coupling constant (*J* = 8.0 Hz) between 4.44 (1′-H) and 3.15 (2′-H) confirmed the presence of β-D-glucose. Hydrogen signals in ^1^H NMR rid of glucose was in accordance with norhyoscyamine (**11**), and mass fragment of 276.1591 for [M+H]^+^ and ultraviolet spectrum consistence assisted this point. Coupling between 102.94 (C-1′) and 4.23, 4.13 (9-H) in HMBC and coupling between 4.44 (1′-H) and 4.23, 4.13 (9-H) in NOESY demonstrated β-D-glucose was linked at C-9 of **12**. Therefore, this compound was identified to be glyconorhyoscyamine. Compound **18** was a cinnamic acid amide. The molecular formula is determined by MS and ^13^C NMR as C_25_H_35_N_3_O_5_ with an m/z ion 458.2647 for [M+H]^+^. The ^1^H and ^13^C spectrum are of high similarity to compound **17** (N1,N10-di-dihydrocaffeoylspermidine), except the removal of a hydroxyl at aromatic ring. Symmetric aromatic carbons 129.88 and 115.42, and corresponding protons 7.05 (d, *J* = 8.4 Hz, 2 H, 3-H, 5-H) and 6.74 (dd, *J* = 8.3, 2 H, 2-H, 6-H) confirmed the presence of 1, 4-disubstituted benzene. The corresponding signals between symmetric carbon 129.88 and 2.76 (H-7) and 2.44 (H-8) in HMBC confirmed the linked position of 1, 4-disubstituted benzene as Fig. 2f shown. This compound was characterized as N1-p-dihydrocoumaroyl-N10- dihydrocaffeoyl spermidine. The specific assignment of the three new compounds was supplied in Supporting information.

Overall, twelve tropane compounds and eight cinnamic acid amides were identified from *S. tangutica* (Fig. 2e and f, Fig. S6 ~ S39). These compounds were isolated in varied quantities with a span of three orders of magnitude from 1.85 mg to 2.8 g. Two new tropane compounds and one new cinnamic acid amide were identified as glycoscopolamine (**8**), glyconorhyoscyamine (**12**), and N1-p-dihydrocoumaroyl -N10-dihydrocaffeoyl spermidine (**18**), respectively, all with small quantity. For instance, compound **8** (fraction F8-2-P4) was purified as a minor peak of the secondary fraction F8-2, which was a small peak in the separation of the original fraction F8. Furthermore, six tropane compounds and three cinnamic acid amides were discovered from *S. tangutica* for the first time. They are characterized as dihydroanisodamine (**5**), dihydroanisodine (**6**), noranisodine^31^ (**7**), noranisodamine (**9**)^32^, deepoxyanisodine^33^ (**10**), norhyoscyamine (**11**)^33^,^34^, N-trans-p- Coumaroylputrescine^35^,^36^ (**13**), N-trans-isoferuloylputrescine^37^,^38^ (**15**) and N-cis- isoferuloylputrescine^38^ (**16**), based on their mass fragment and NMR spectrum. The elucidation and specific assignments of these compounds were in supporting information. Other known compounds are isolated as scopolamine^39^,^40^ (**1**), anisodamine^41^,^42^ (**2**), hyoscyamine^43^,^44^ (**3**), anisodine^31^ (**4**), N-Caffeoylputrescine^36^,^45^,^46^ (**14**), N1,N10-di-dihydrocaffeoylspermidine^21^,^47^ (**17**), Scotanamine D^21^ (19), and N1-caffeoyl-N3-dihydrocaffeoyl spermidine^48^ (**20**)

### Pharmacological characterization of tropane compounds on M3 receptor

**Figure 3:**
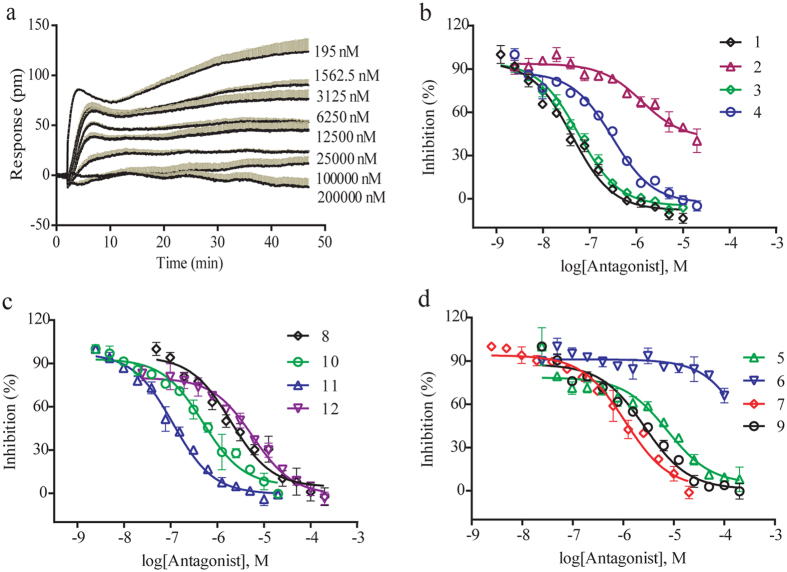
Label-free pharmacological profiling of tropane alkaloids on M3 receptor in HT-29 cells. (**a**) Real-time DMR response of 16 μM acetylcholine after pretreatment with compound **8** at different doses. (**b**–**d**) The dose-dependent inhibition of twelve tropane compounds on the DMR amplitudes at 30 min post-stimulation with 16 μM acetylcholine. Data were represented as mean ± s.d.

We systematically characterized the pharmacology of twelve tropane compounds using a two-step DMR assay on M3 receptor in HT-29. Results show that all these compounds induce little DMR response in the first step (exemplified by compound **8** and compound **12** in Fig. S40), but all dose-dependently inhibit the acetylcholine-induced DMR in the second step (exemplified by compound **8** and compound **12** in Fig. 3a and Fig. S40). Among the four known tropane alkaloids, scopolamine (**1**), hyoscyamine (**3**) and anisodine (**4**) are potent antagonists with IC_50_ values of 0.04 ± 0.01 μM, 0.06 ± 0.01 μM, and 0.31 ± 0.10 μM, respectively. However, anisodamine (**2**) displays partial efficacy, but moderate potency, to inhibit the acetylcholine DMR, indicating that anisodamine may be an allosteric modulator of M3 receptor. The order of inhibition potency is **1** > **3** > **4** > **2,** in consistent with previous report^49^.

Two new ligands **8** and **12** are also active to antagonize M3 receptor, resulting in IC_50_ values of 1.97 ± 0.88 μM and 4.47 ± 1.98 μM, respectively. Compounds **5, 6, 7, 9, 10** and **11** are atropine analogs isolated from *S. tangutica* for the first time and their inhibition values on M3 receptor are first reported here, as 7.48 ± 4.12 μM, 69.9 μM (weak), 1.11 ± 0.40 μM, 2.64 ± 1.05 μM, 0.49 ± 0.17 μM and 0.11 ± 0.02 μM respectively. All drugs dose-dependently block the acetylcholine DMR (Fig. 3c and Fig. 3d), yielding a potency order of **1** > **11** > **10** > **7** > **8** > **9** > **12** > **5** > **6**. Of note, **6** does not fully inhibit the acetylcholine DMR at the highest dose.

The twelve tropane ligands isolated from *S. tangutica* are atropine analogs and share high similarity in structure, permitting structure-activity relationship analysis. Among these compounds, the potency of **1** is comparable as that of **3**, indicating that the introduction of an epoxide ring at R_1_ and R_2_ does not damage the antagonistic potency of the backbone. Centered with **1**, bonding hydroxyl at R_5_ (**4**) decreases its potency by about 8 folds. The introduction of hydroxyl at R_5_ and removal of methyl at R_3_ (**7**) reduce the activity by approximately 25 folds. Replacing hydroxyl at R_4_ with glucose group (**8**) exhibits about 50-fold decrease in inhibitory activity. Centered with **3**, the introduction of hydroxyl at R_5_ (**10**) and removal of methyl at R_3_ (**11**) slightly reduce activity, while addition of hydroxyl at R_1_ (**2**) greatly reduce activity to partial inhibition level. Placing hydroxyl at both R_1_ and R_5_ is **6,** which is nearly inactive on M3 receptor. Overall, the modifications of R_1_ and R_2_ are vital to regulate the activity of hyoscyamine (**3**). Introduction of the epoxide ring at R_1_ and R_2_ (**1**) have little effect, but presence of hydroxyl at R_1_ or R_2_ (**2**) or both (**5**) damage inhibitory activity tremendously. Removal of methyl at R_3_ (**3** vs **11** and **4** vs **7**) reduced activity fewer than 5 folds. This structure-activity relationship will provide guidance for the design of lead compounds for M3 receptor.

### Pharmacological characterization of cinnamic acid amides

**Figure 4:**
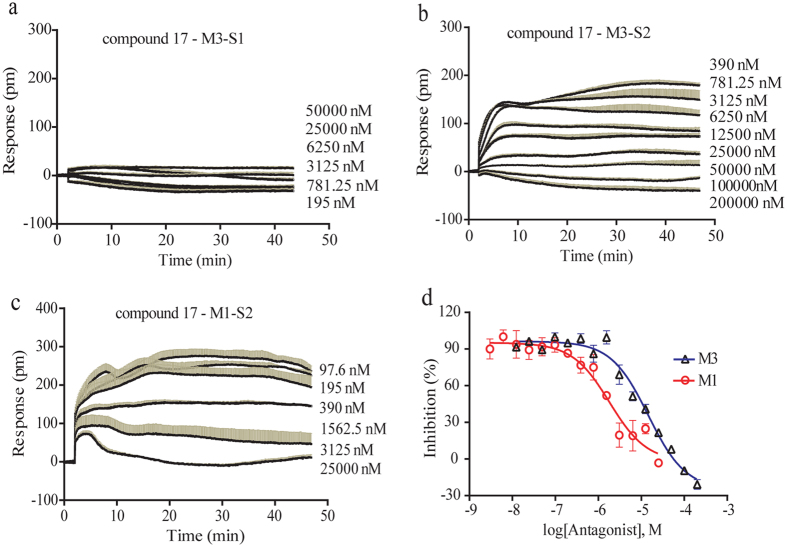
Selectivity of compound **17** for M3 and M1 receptors. (**a**) Real-time DMR responses of compound **17** at different doses in HT-29. (**b**) Real-time DMR responses of 16 μM acetylcholine with pretreatment by **17** at varied doses in HT-29. (**c**) Real-time DMR responses of 4 μM acetylcholine in the presence of dosed **17** in the M1-transfected CHO cells. (**d**) The dose-dependent inhibition of **17** on the DMR amplitudes at 30 min post stimulation with acetylcholine. Data were represented as mean ± s.d.

We examined the pharmacology of eight cinnamic acid amides on M3 receptor in HT-29 using the two-step DMR assay. As a result, among these cinnamic acid amides, only **17** is active to antagonize M3 receptor, as it stimulates little response in HT-29 cells and dose-dependently inhibited the acetylcholine DMR (Fig. 4a and Fig. 4b). The two-dimensional purification and mass spectrum of this active compound were shown in Fig. S5. Considering that **17** is a novel chemical ligand displaying M3 antagonistic activity, we examined its selectivity over M1 receptor. Result showed that **17** also dose-dependently inhibited the acetylcholine DMR (Fig. 4c) in the M1-transfected CHO cells. The dose-dependent inhibition on M3 and M1 were presented in Fig. 4d and IC_50_ values obtained were 15.57 ± 5.09 μM and 1.82 ± 0.86 μM for M3 in HT-29 and M1 in CHO-M1 cells, respectively. Together, compound **17** is a novel muscarinic receptor antagonist.

### Competitive antagonism of alkaloid compounds

**Figure 5:**
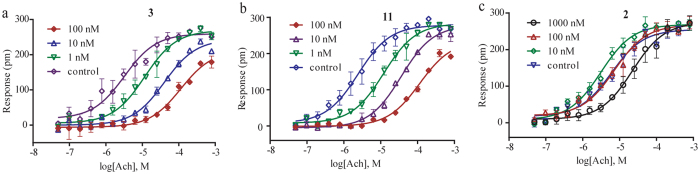
Competitive antagonism of alkaloid compounds. (**a**–**c**) The dose responses of acetylcholine at 30 min post-stimulation in the presence of **3, 11** and **2,** each at different, fixed doses. Here, the compound and acetylcholine are added to co-stimulate HT-29 cells. The control is without addition of compounds. Data were represented as mean ± s.d.

We applied a co-stimulation DMR assay to determine whether active alkaloid compounds are competitive antagonists or not. Here, acetylcholine at a series of concentrations were prepared in the presence of compound **2**, **3**, and **11**, each at a fixed dose, and then used to co-stimulate HT-29 cells. The three compounds display diverse pharmacological actions. The control without existence of compound is in the left and both **3** and **11** dose-dependently shift the acetylcholine dose curve to right (Fig. 5a and 5b), suggesting that both act as a competitive antagonist for M3 receptor. In contrast, compound **2** (anisodamine) exhibited complicated effect; at a low dose (10 nM) it increases the potency of acetylcholine, but at a high dose (1 μM) it decreases the potency of acetylcholine (Fig. 5c). Further investigation of the biochemical mechanism of these compounds will be of great interest.

## Discussion

What is critical to isolate new bioactive alkaloids from traditional Chinese medicinal plants is to have a high-resolution separation system and an effective activity-guided protocol. The separation system requires high separation selectivity, resolution and symmetric peak shape so that alkaloid products can be pure enough for structure determination and activity identification. This is particularly essential for isolation and identification of minor active alkaloids that have great potential to be new ligands, compared to abundant constitutes. *S. tangutica* is a promising TCM to discover novel bioactive drugs from its alkaloid constituents in large-scale multi-dimension preparation. We here applied a large-scale multi-dimensional preparation approach to systematically isolate compounds from *S. tangutica.* Considering the diversity of chemical constituents in a medicinal plant, bioassay-guided separation is a good strategy to improve efficiency and precision in the discovery of active ligands. We here employed an unbiased and label-free cell phenotypic profiling approach to guide the isolation of minor active compounds from *S. tangutica.*

As a result, we have isolated for the first time five tropane alkaloids **7, 8, 9, 10** and **12,** and demonstrated their antagonistic activity on M3 receptor. Among them, two glucuronide conjugated scopolamine analogs (**8** and **12**) are similar to metabolites of scopolamine and anisodine, and **7** is one of the metabolites of scopolamine^31^,^50^, implicating that the metabolites of muscarinic receptor antagonists may also contribute to their *in vivo* effects. Of note, there are a great number of other minor or trace compounds remaining as a mixture of isomers or homologues and in low quantity in *S. tangutica*. Further study of them is important to discover new active ones. These results presents the power of the label-free cell phenotypic profiling-guided preparation protocol to isolate and discover novel chemicals from traditional Chinese medicinal plants, and this technique can also be extended to a wide range of TCM plants.

## Methods

### Materials

*S. tangutica* was from Northwest Institute of Plateau Biology, Chinese Academy of Sciences. XCharge C18 and XCharge SCX columns were purchased from Acchrom Co. (Dalian, China). Na_2_SO_4_ and NaH_2_PO_4_ were from Sinopharm Chemical Reagent Co. (Beijing, China). Formic acid (FA) and phosphoric acid were bought from J&K Chemical Co. (Shanghai, China). Acetonitrile (ACN) and ethanol were purchased from Fulltime corporation in Anhui province. The mass spectrum information of purified compounds was obtained using Agilent 1290 Infinity UPLC and 6540 UHD Q-TOF and Mass Hunter software was used to process mass spectra data. The structures of compounds were identified with Bruker 400 MHz NMR spectrometer and Bruker 500 MHz NMR spectrometer. Optical rotation values were tested using Jasco P-1020 polarimeter (Japan); infrared spectrums (IR) were obtained by Bruker Tensor27 FTIR spectrometer; ultraviolet (UV) spectrums were acquired with Shimadzu UV-2401PC spectrometer (Japan).

Acetylcholine chloride was from Sigma Chemical Co. (St Louis, MO, USA). DMR assay was performed on Epic^®^ BT system with Epic^®^ 384-well biosensor cell culture microplate (Corning, NY, USA). Human colorectal adenocarcinoma cell line (HT-29) and Human lung adenocarcinoma cell (A549) were purchased from the cell bank of Shanghai Institute of Cell Biology, Chinese Academy of Sciences. M1-transfected CHO cell line (CHO-M1) was kindly given by Professor Olivier Civelli in University of California, Irvine. All compounds were dissolved in dimethyl sulfoxide (DMSO), stocked in 50 mM and diluted by HBSS to the necessary concentrations.

### Purification of alkaloids compounds from *S. tangutica*

50 kg of *S. tangutica* tuber was crushed and extracted with 95% ethanol. After concentration, an XCharge SCX (60 μm, dia.) was used to enrich alkaloids from the extraction. Alkaloid constituents was separated on the first dimension chromatography on an XCharge C18 (100 mm × 316 mm, I.D.; 7 μm, dia.). The optimized mobile phase was: A: ACN; B: 200 mM Na_2_SO_4_; and C: H_2_O; keeping B at 10%, the gradient condition from 5% A to 15% A over 30 min at a flow rate of 330 mL/min. Peaks were recorded at 210 nm and 23 factions were collected.

The second dimensional preparations of active fractions F5 to F18 were performed on the XCharge SCX (100 mm × 290 mm, I.D.; 7 μm, dia.), and the mobile phase was: A: ACN; B: 100 mM NaH_2_PO_4_ (pH = 2.83); C: H_2_O. Keeping B at 30%, ACN shifted from 35% to 50% over 30 min at a flow rate of 320 mL/min, and the peaks were monitored at 210 nm.

The third dimensional fractions were purified on the XCharge C18 (20 mm × 250 mm, I.D.; 7 μm, dia.) using HPLC with 2525 binary pump and 2489 photodiode array detection system (waters, USA). The mobile phase condition was: A: ACN; B: 0.1% FA in H_2_O (v/v); 6% A for compound **18**, **19** and **20**; 4% A for **1, 2, 11, 12,** and **17**; 3% A for **3** and **13**; 2% A for **8, 9, 10, 13, 14, 15** and **16**; 0% A for **4, 5, 6** and **7** at a flow rate of 20 mL/min.

### Cell culture

The mediums used to culture HT-29, CHO-M1 and A549 were McCoy’s 5 A, F12 and F12K (Sango Biotech, Shanghai, China), respectively. 10% fetal bovine serum (Gibco, Life Technologies) was supplemented. Penicillin and streptomycin were used with the concentration of 50 μg/mL and 100 μg/mL, respectively.

### Dynamic mass redistribution (DMR) assays

All DMR assays were performed using an Epic^®^ BT system. HT-29 cells and A549 cells were seeded in Epic^®^ 384-well biosensor microplate (Corning) with a density of 32000 cells per well and 15000 cells per well, respectively. Then the microplate was cultured for 22 hrs or 15 hrs in the corresponding cell medium to form a confluent monolayer for HT-29 or CHO-M1 cells, respectively. After being washed, the cells were maintained with assay buffer and incubated for 1 hr.

For profiling of the fractions, a 2-min baseline was first established, followed by adding twenty three fractions at 1.25 mg/L and recording the fraction-induced DMR signals for 1 hr. Then, after 2-min baseline was re-established, acetylcholine at 16 μM and histamine at 8 μM were added for HT-29 and A549 cell line, respectively. The DMR responses were recorded for another 1 hr.

For the IC_50_ determination of pure compounds, a 2-min baseline was first established. Then compounds at varied doses were added manually and the DMR response was recorded for 1 h. Afterwards a 2-min baseline was re-established, and acetylcholine at 16 μM and acetylcholine at 4 μM were added to HT-29 and CHO-M1, respectively. DMR response was monitored for another 1 h.

For co-stimulation assays, a 2-min baseline was first established. Acetylcholine dose series in the absence and presence of a given ligands at fixed doses were used to stimulate the HT-29 cells.

### Data analysis

Data process and analysis were performed on Microsoft excel 2010 and GraphPad Prism 6.02 (GraphPad Software Inc., San Diego, CA, USA). All IC_50_ values reported were shown as mean ± standard deviation in duplicate from two dependent experiments (n = 4). The heat map was completed by Cluster 3.0 and Treeview after processing in Microsoft excel 2010.

## Additional Information

**How to cite this article:** Du, N. *et al*. Discovery of new muscarinic acetylcholine receptor antagonists from *Scopolia tangutica. Sci. Rep.* **7**, 46067; doi: 10.1038/srep46067 (2017).

**Publisher's note:** Springer Nature remains neutral with regard to jurisdictional claims in published maps and institutional affiliations.

## Supplementary Material

Supplementary Information
