# Supplementary material for: Discovery of new muscarinic acetylcholine receptor antagonists from Scopolia tangutica
Source: Sci Rep. 2017 Apr 7;7:46067. doi: 10.1038/srep46067 (PMC5384254; doi:10.1038/srep46067)
Supplement: Supplementary Information [file srep46067-s1.doc]

**Supporting information**

**Discovery of new muscarinic acetylcholine receptor antagonists from *Scopolia tangutica***

Nana Du ^a,b^, Yanfang Liu *^a^, Xiuli Zhang ^a^, Jixia Wang ^a^, Jianqiang Zhao ^b,c^, Jian He ^a^, Han Zhou ^a^, Lijuan Mei ^c^ and Xinmiao Liang *^a,d^

^a^ Key Lab of Separation Science for Analytical Chemistry, Dalian Institute of Chemical Physics, Chinese Academy of Sciences, Dalian, China

^b^ University of Chinese Academy of Sciences, Beijing, China

^c^ Key Lab of Tibetan Medicine Research, Northwest Institute of Plateau Biology, Chinese Academy of Sciences, Xining 810008, China

^d^ Co-innovation Center of Neuroregeneration, Nantong University, Nantong, 226019, China

*Corresponding authors: Yanfang Liu, Xinmiao Liang

Tel.: +86 411 84379541; fax: +86 411 84379539.

E-mail addresses: [liuyanfang@dicp.ac.cn](mailto:liuyanfang@dicp.ac.cn), liangxm@dicp.ac.cn

**Table of contents**

Fig. S1 The analysis of fractions F1 ~ F23 on XCharge C18.

Fig. S2 The analysis of fractions F1 ~ F23 on XCharge SCX.

Fig. S3 TIC of F16-7-P6.

Fig. S4 TIC of F16-7-P7.

Fig. S5 Purificantion of compound **17**.

Fig. S6 ^1^H spectrum of **5** in D_2_O.

Fig. S7^1^H spectrum of **6** in D_2_O.

Fig. S8 DEPT 135 spectrum of **6** in D_2_O.

Fig. S9 HSQC spectrum of **6** in D_2_O.

Fig. S10 ^1^H spectrum of **7** in D_2_O.

Fig. S11 ^13^C spectrum of **8** in D_2_O.

Fig. S12 ^1^H spectrum of **8** in D_2_O.

Fig. S13 HSQC spectrum of **8** in D_2_O.

Fig. S14^1^H, ^1^H-COSY spectrum of **8** in D_2_O.

Fig. S15 DEPT 135 spectrum of **8** in D_2_O.

Fig. S16 NOESY spectrum of **8** in D_2_O.

Fig. S17 HMBC spectrum of **8** in D_2_O.

Fig. S18 ^1^H spectrum of **9** in D_2_O.

Fig. S19 DEPT 135 spectrum of **9** in D_2_O.

Fig. S20 HSQC spectrum of **9** in D_2_O.

Fig. S21 ^1^H spectrum of **10** in D_2_O.

Fig. S22 DEPT 135 spectrum of **10** in D_2_O.

Fig. S23 HSQC spectrum of **10** in D_2_O.

Fig. S24 ^1^H spectrum of **11** in D_2_O.

Fig. S25 DEPT 135 spectrum of **11** in D_2_O.

Fig. S26 HSQC spectrum of **11** in D_2_O.

Fig. S27 ^1^H, ^1^H-COSY spectrum of **11** in D_2_O.

Fig. S28 ^1^H spectrum of **12** in D_2_O.

Fig. S29 ^13^C spectrum of **12** in D_2_O.

Fig. S30 HSQC spectrum of **12** in D_2_O.

Fig. S31 DEPT 135 spectrum of **12** in D_2_O.

Fig. S32 HMBC spectrum of **12** in D_2_O.

Fig. S33 H,H-COSY spectrum of **12** in D_2_O.

Fig. S34 NOESY spectrum of **12** in D_2_O.

Fig. S35 ^1^H spectrum of **18** in D_2_O.

Fig. S36 ^13^C spectrum of **18** in D_2_O.

Fig. S37 HSQC spectrum of **18** in D_2_O.

Fig. S38 H,H-COSY spectrum of **18** in D_2_O.

Fig. S39 HMBC spectrum of **18** in D_2_O.

Fig. S40 Label-free pharmacological profiling of compound **8** and **12** on M3 receptor in HT-29 cells.


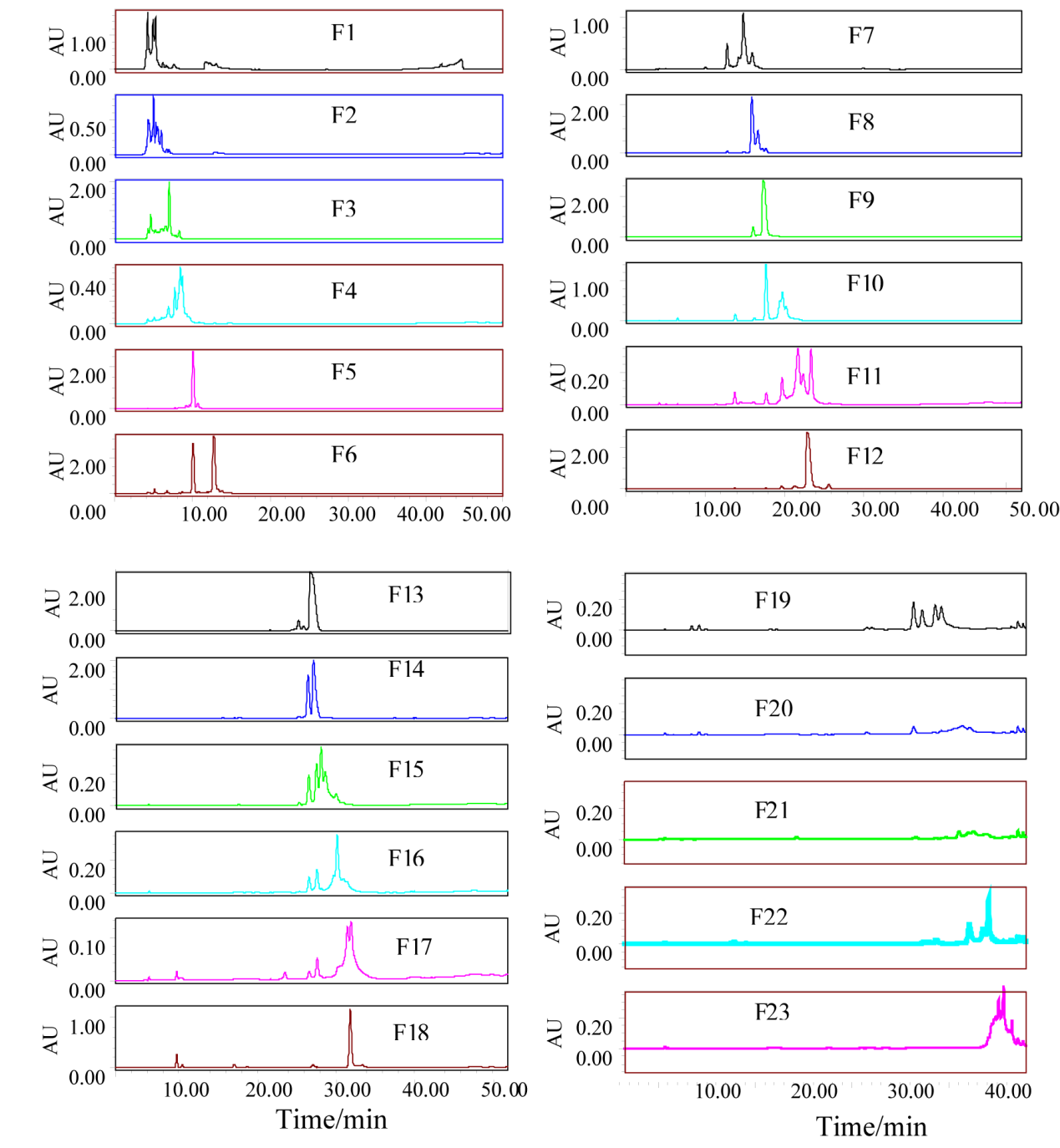


Fig. S1 The analysis of fractions F1 ~ F23 on XCharge C18. Twenty-three fractions were analyzed on the column XCharge C18 (4.6 mm × 250 mm, I.D.; 7 µm, dia.). The applied mobile phase was: A: ACN, B: 200 mmol/L Na_2_SO_4_, and C: H_2_O, keeping B at 20%, the gradient condition from 5% A to 15% A over 30 min at a flow rate of 1.0 mL/min. Peaks were recorded at 210 nm.


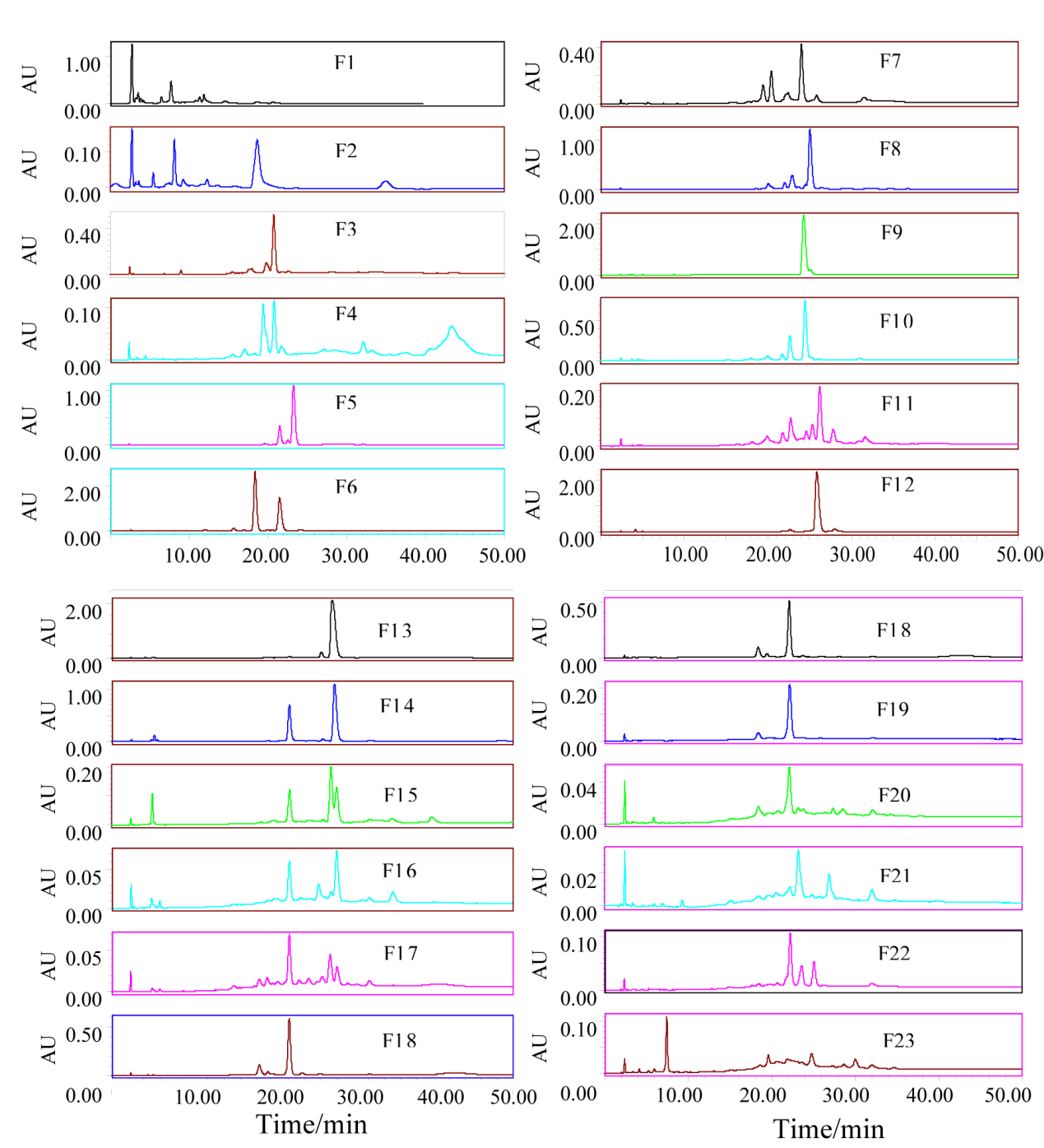


Fig. S2 The analysis of fractions F1 ~ F23 on XCharge SCX. Fractions F3 ~ F23 were analyzed on the column XCharge SCX (4.6 mm × 250 mm, I.D.; 7 µm, dia.), and the mobile phase was: A: ACN, B: 100 mmol/L NaH_2_PO_4_ (pH = 2.83), C: H_2_O. Keeping B at 30%, ACN shifted from 35% to 50% over 30 min at a flow rate of 1.0 mL/min, and the peaks were monitored at 210 nm. For fractions F1 and F2, isocratic condition 30% B, 50% A and 20% C was applied to get the full peaks.


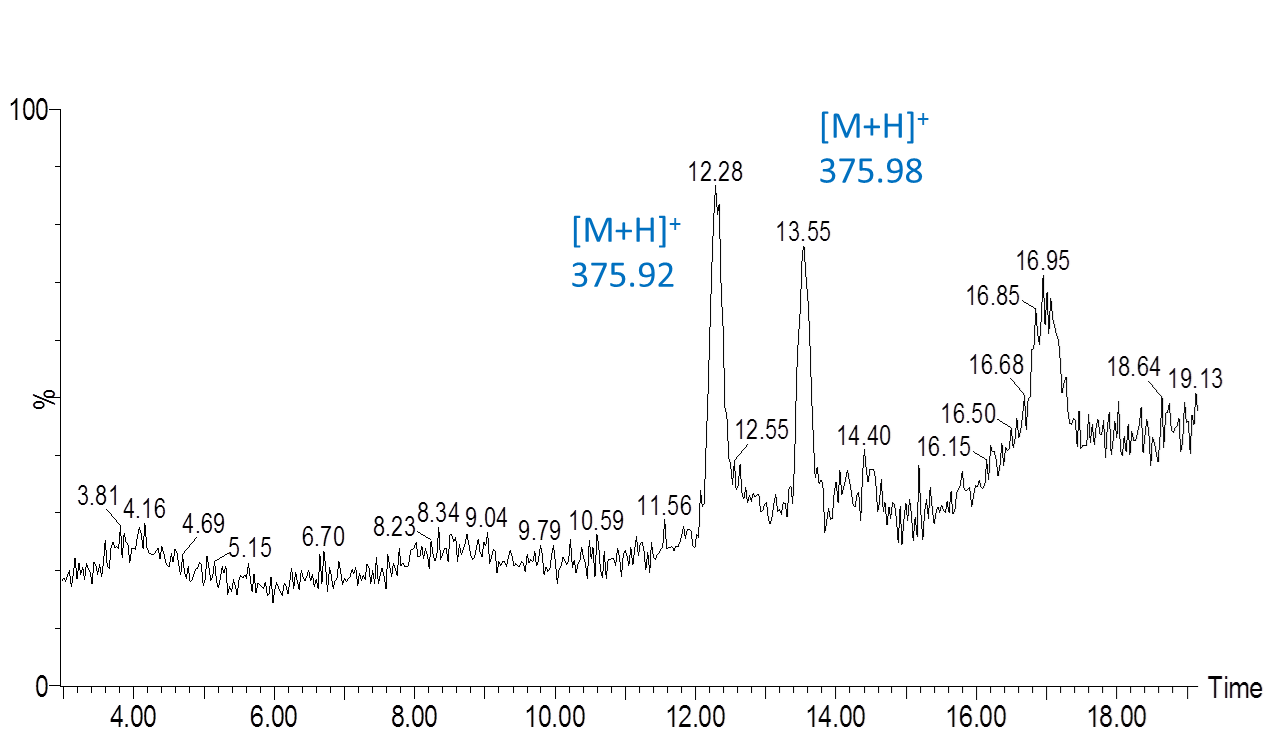


Fig. S3 TIC of F16-7-P6. The separation was performed on the column C18TDE (2.1 mm × 150 mm, I.D.; 7 µm, dia.). The mobile phase was: A: 0.1%FA/ACN, B: 0.1%FA/H_2_O, and A shifted from 5% to 50% over 25 min at a flow rate of 0.2 mL/min. The ESI-Q was applied at positive mode, with a caplliary voltage of 2.5 kV and cone voltage of 25V.


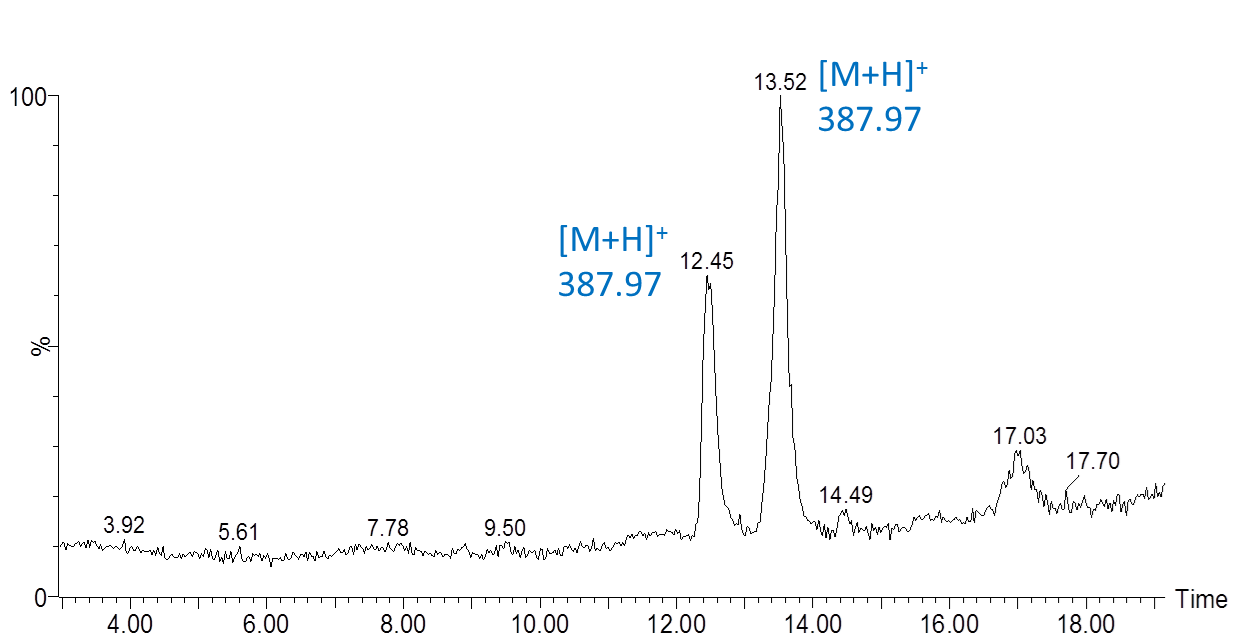


Fig. S4 TIC of F16-7-P7. The separation was performed on the column C18TDE (2.1 mm × 150 mm, I.D.; 7 µm, dia.). The mobile phase was: A: 0.1%FA/ACN, B: 0.1%FA/H_2_O, and A shifted from 5% to 50% over 25 min at a flow rate of 0.2 mL/min. The ESI-Q was applied at positive mode, with a caplliary voltage of 2.5 kV and cone voltage of 25V.


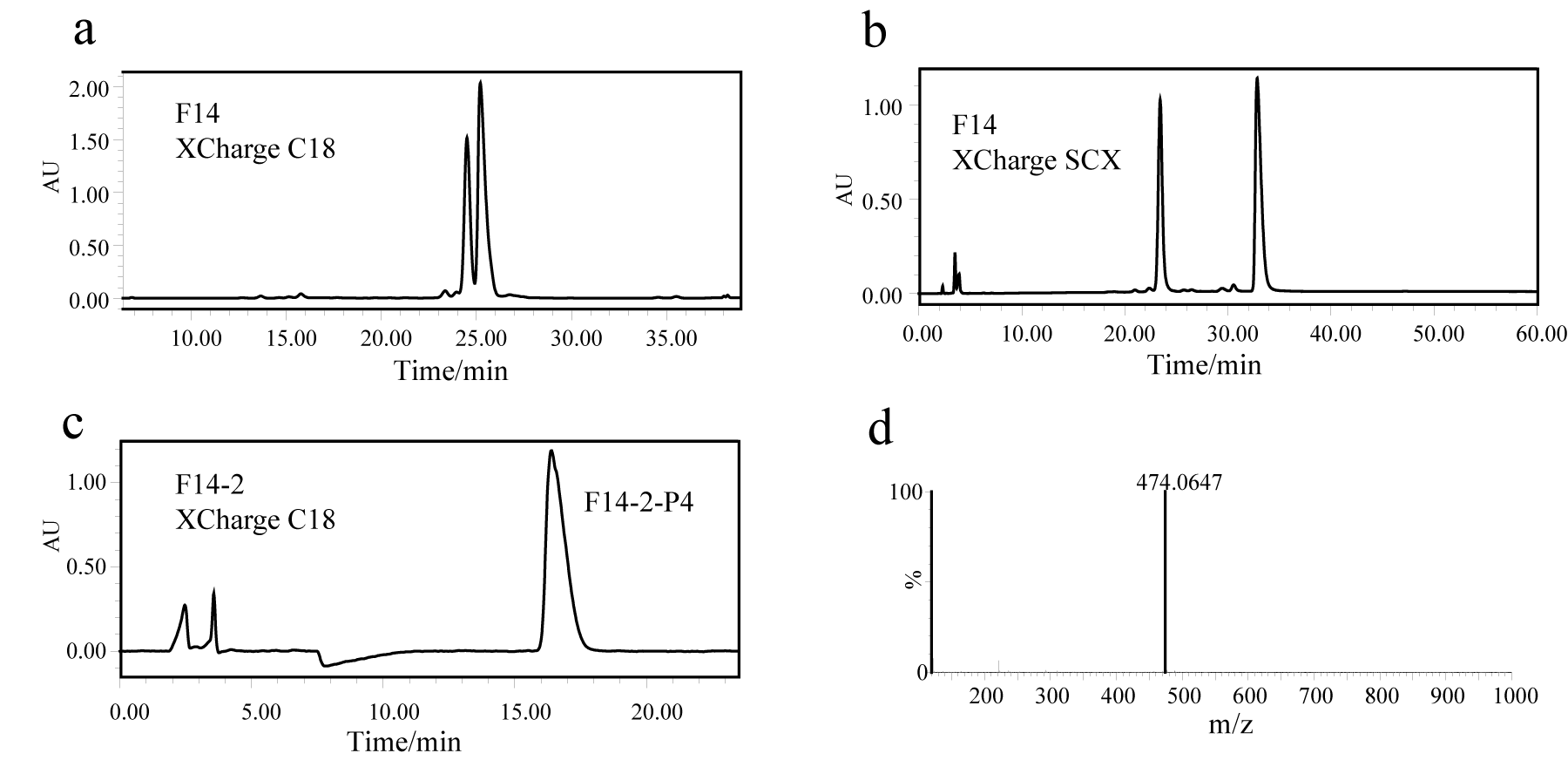


Fig. S5 Purificantion of compound **17**. (a) Chromatography of F14 on an XCharge C18 column in the first dimensional separation (1D) method. (b) Chromatography of F8 on XCharge SCX column in the second dimension. (c) Chromatography of F14-2 on an XCharge C18 column in the third dimension. (a-c) were all detected in a wavelength of 210 nm. (d) MS spectrum of F14-2-P4 (**17**) at a positive ESI mode.

**The information of isolated compounds**

Compound **1** (scopolamine)

Compound **1** was isolated as a white power. The purity and molecular formula were identified in HPLC-ESI-MS. The exact data of m/z in positive mode was 304.1613 (calculated for [M+H]^+^ = 304.1471 ), with a molecular formula C_17_H_21_NO_4_. The optical rotation of this compound, [α]25 D, was detected as -28.000. Assisted by MS and NMR information, it was identified as scopolamine. The NMR assignment was as follows.

1H NMR (400 MHz, D2O) δ 7.46 – 7.22 (m, 5H, benzene-H), 4.97 (t, J = 5.0 Hz, 1H, 3-H), 4.10 (dd, J = 13.2, 10.3 Hz, 1H, 7-H), 3.86 (dt, J = 6.9, 4.5 Hz, 3H, 5-H, 9-H, 9-H), 3.71 (d, J = 3.4 Hz, 2H, 15-H, 1-H), 2.96 (d, J = 3.3 Hz, 1H, 16-H), 2.72 (s, 3H, 17-H), 2.43 – 2.25 (m, 2H, 2-H, 4-H), 1.94 (d, J = 17.3 Hz, 1H, 4-H), 1.73 (d, J = 17.3 Hz, 1H, 2-H).

Compound **2** (anisodamine)

Compound **2** was isolated as a white power. The purity and molecular formula were identified in HPLC-ESI-MS. The m/z in positive mode was 306.1805 and molecular formula C_17_H_23_NO_4_. The optical rotation of this compound, [α]25 D, was detected as -17.808. Combining MS and NMR information, it was identified as anisodamine. The NMR assignment was as follows.

^1^H NMR (400 MHz, D_2_O) δ 7.47 – 7.18 (m, 5H, benzene-H), 4.93 (t, J = 4.4 Hz, 1H, 3-H), 4.42 – 4.31 (m, 1H, 16-H), 4.12 (dd, J = 13.5, 10.3 Hz, 1H, 9-H), 3.88 (dt, J = 23.5, 10.0 Hz, 3H, 9-H, 1-H, 5-H), 3.66 (s, 1H, 7-H), 2.84 (s, 3H, 17-H), 2.30 – 2.05 (m, 3H, 15-H, 15-H, 4-H), 1.84 (q, J = 16.2 Hz, 3H, 2-H, 2-H, 4-H).

Compound **3**

Compound **3** was isolated as a white power. The purity and molecular formula were identified in HPLC-ESI-MS. The m/z in positive mode was 290.1758 and molecular formula was C_17_H_23_NO_3_. The optical rotation of this compound, [α]25 D, was detected as -28.261. Assisted by MS and NMR information, it was identified as hyoscyamine. The NMR assignment was as follows.

^1^H NMR (400 MHz, D2O) δ 7.32 (dt, J = 12.4, 7.3 Hz, 5H, benzene-H), 4.96 (t, J = 4.8 Hz, 1H, 3-H), 4.10 (dd, J = 9.5, 6.7 Hz, 1H, 7-H), 3.89 (m, 2H, 5-H), 3.85 (dd, J = 9.6, 6.7 Hz, 1H, 9-H), 3.73 (s, 1H, 9-H), 3.66 – 3.56 (m, 1H, 1-H), 2.62 (s, 3H, 17-H), 2.30 – 2.14 (m, 2H, 4-H, 2-H), 2.03 (dd, J = 10.2, 5.8 Hz, 3H, 4-H, 15-H, 15-H), 1.85 (d, J = 16.5 Hz, 2H, 2-H, 16-H), 1.54 – 1.43 (m, 1H, 16-H).

Compound **4** (anisodine)

Compound **4** was isolated as a white power. The purity and molecular formula were identified in HPLC-ESI-MS. The m/z in positive mode was 319.1512 and molecular formula C_17_H_21_NO_5_. The optical rotation of this compound, [α]25 D, was detected as -23.786. Combining MS and NMR information, it was identified as anisodine. The NMR assignment was as follows.

^1^H NMR (400 MHz, D_2_O) δ 7.55 (d, J = 7.5 Hz, 2H, benzene-H), 7.37 (t, J = 7.4 Hz, 2H, benzene-H), 7.31 (t, J = 7.1 Hz, 1H, benzene-H), 4.91 (s, 1H, 3-H), 4.13 (d, J = 11.0 Hz, 2H, 7-H, 9-H), 3.97 (d, J = 2.8 Hz, 1H, 1-H), 3.90 (d, J = 13.8 Hz, 2H, 9-H, 15-H), 3.74 (d, J = 2.7 Hz, 1H, 16-H), 3.59 (d, J = 11.0 Hz, 1H, 5-H), 2.73 (d, J = 4.3 Hz, 3H, 17-H), 2.44 – 2.33 (m, 2H, 2-H, 4-H), 1.82 (d, J = 16.9 Hz, 1H, 4-H), 1.76 (d, J = 16.9 Hz, 1H, 2-H).

Compound **5** was isolated as a white power. UV (H_2_O) λmax (Abs.): 203 nm(0.1730); IR (KBr) νmax: 3421 (-OH), 1731 (-COO-),1634 cm^−1^(benzene); [α]25 D -24.675 (H_2_O). The purity and molecular formula were identified in HPLC-ESI-MS. The m/z in positive mode was 322.1653 (calculated for [M+H]^+^ = 322.1576) and molecular formula was C_17_H_23_NO_5_. The predominant fragment ions were 322.1652, 156.1018 and 138.0911, results of hydrolysis of ester group and loss of H_2_O successively, which was in accordance with dihydroanisodamine. The NMR spectrum was highly similar to that of anisodamine except the addtion of hydroxyl group at C-15. Therefore, it was confirmed as dihydroanisodamine. The stereo-configuration of chiral carbons was difficult to judge without referable data, though optical rotation was given. The NMR assignment was listed as follows.

^1^H NMR (400 MHz, D_2_O) δ 7.45 – 7.23 (m, 5H, benzene-H), 4.93 (s, 1H, 3-H), 4.44 – 4.25 (m, 1H, 9-H), 4.10 (ddd, J = 13.5, 10.2, 3.0 Hz, 1H, 9-H), 3.86 (ddd, J = 34.0, 19.5, 17.2 Hz, 3H, 16-H, 15-H, 1-H), 3.67 (d, J = 7.2 Hz, 1H, 5-H), 3.54 (dd, J = 14.0, 5.8 Hz, 1H, 7-H), 2.85 (s, 3H, 17-H), 2.45 – 2.09 (m, 3H, 4-H, 4-H, 2-H), 1.95 (t, J = 17.1 Hz, 1H, 2-H).

DEPT 135 (400 MHz, D_2_O) δ 129.22 (C-11, C-13), 128.31 (C-12), 128.25 (C-10), 128.23 (C-14), 70.44 (C-16), 70.16 (C-15), 64.84 (C-3), 61.75 (C-9), 53.29 (C-7), 41.38 (C-17), 32.70 (C-2), 32.53 (C-4).


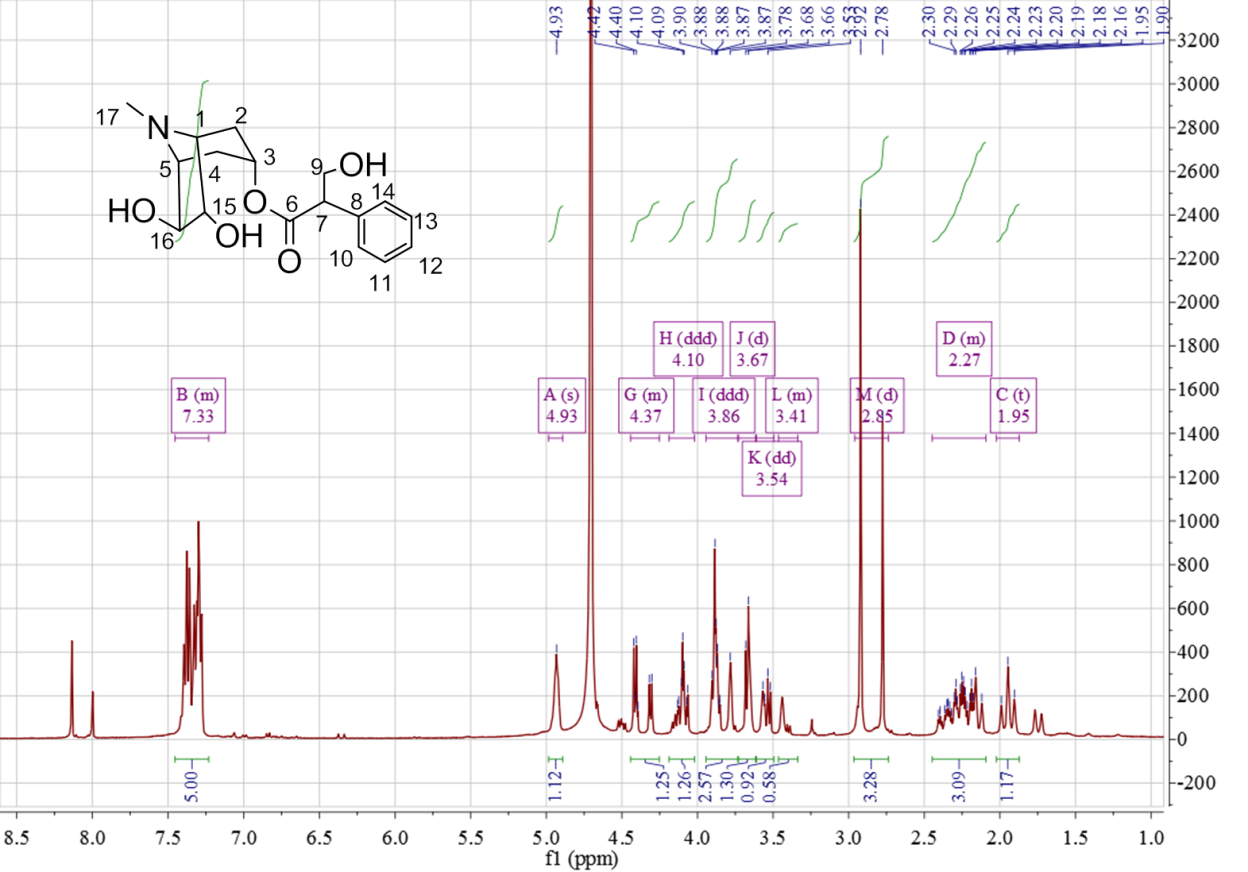


Fig. S6 ^1^H spectrum of **5** in D_2_O.

Compound **6** was isolated as a white power. UV (H_2_O) λmax (Abs.): 203 nm (0.2048); IR (KBr) νmax: 3403 (-OH), 1735 (-COO-), 1634 cm^−1^(benzene) ; [α]25 D -14.286 (H_2_O). The purity and molecular formula were identified in HPLC-ESI-MS. The m/z in positive mode was 322.1653 (calculated for [M+H]^+^ = 322.1576) and molecular formula C_17_H_23_NO_5_. The predominant fragment ions were 322.1657, 158.1176 and 140.1070, results of hydrolysis of ester group and loss of H_2_O successively, which was in accordance with dihydroanisodine. The NMR spectrum was highly similar to that of anisodamine except the lack of proton at C-7. Therefore, it was confirmed as dihydroanisodine. The chiral carbons (C-1, C-3, C-5, C-7, C-15) were the same as anisodamine (**2**) and the optical rotation value was also similar to **2**. Thus, the configuration of chiral carbons was concluded to be the same as **2**. The assignment of NMR information was listed as follows.

^1^H NMR (400 MHz, D_2_O) δ 7.52 – 7.32 (m, 5H, benzene-H), 4.92 (t, J = 4.6 Hz, 1H, 3--H), 4.32 (d, J = 11.8 Hz, 1H, 9-H), 4.20 (dd, J = 7.8, 3.4 Hz, 1H, 16-H), 3.96 (d, J = 11.7 Hz, 1H, 9-H), 3.84 (d, J = 6.8 Hz, 1H, 1-H), 3.63 (s, 1H, 5-H), 2.81 (d, J = 15.0 Hz, 3H, 17-H), 2.41 – 2.03 (m, 3H, 15-H, 15-H, 4-H), 1.94 – 1.63 (m, 3H, 2-H, 2-H, 4-H).

DEPT 135 (400 MHz, D_2_O) δ 129.05 (C-11, C-13), 128.98 (C-12), 125.35 (C-10, C-14), 71.42 (C-16), 69.77 (C-5), 66.33 (C-3), 65.82 (C-9), 62.90 (C-1), 40.14 (C-17), 34.55 (C-2), 33.51 (C-4), 32.33 (C-15).


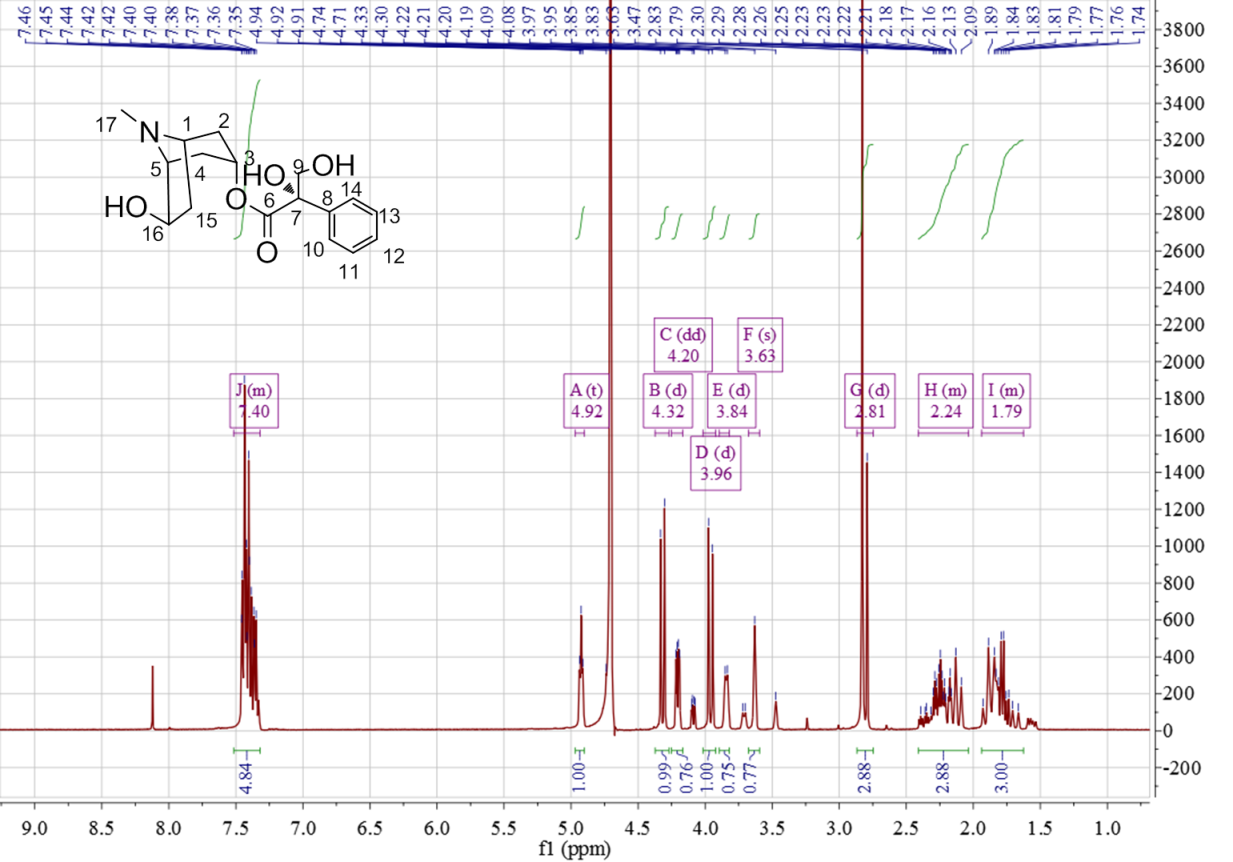


Fig. S7 ^1^H spectrum of **6** in D_2_O.


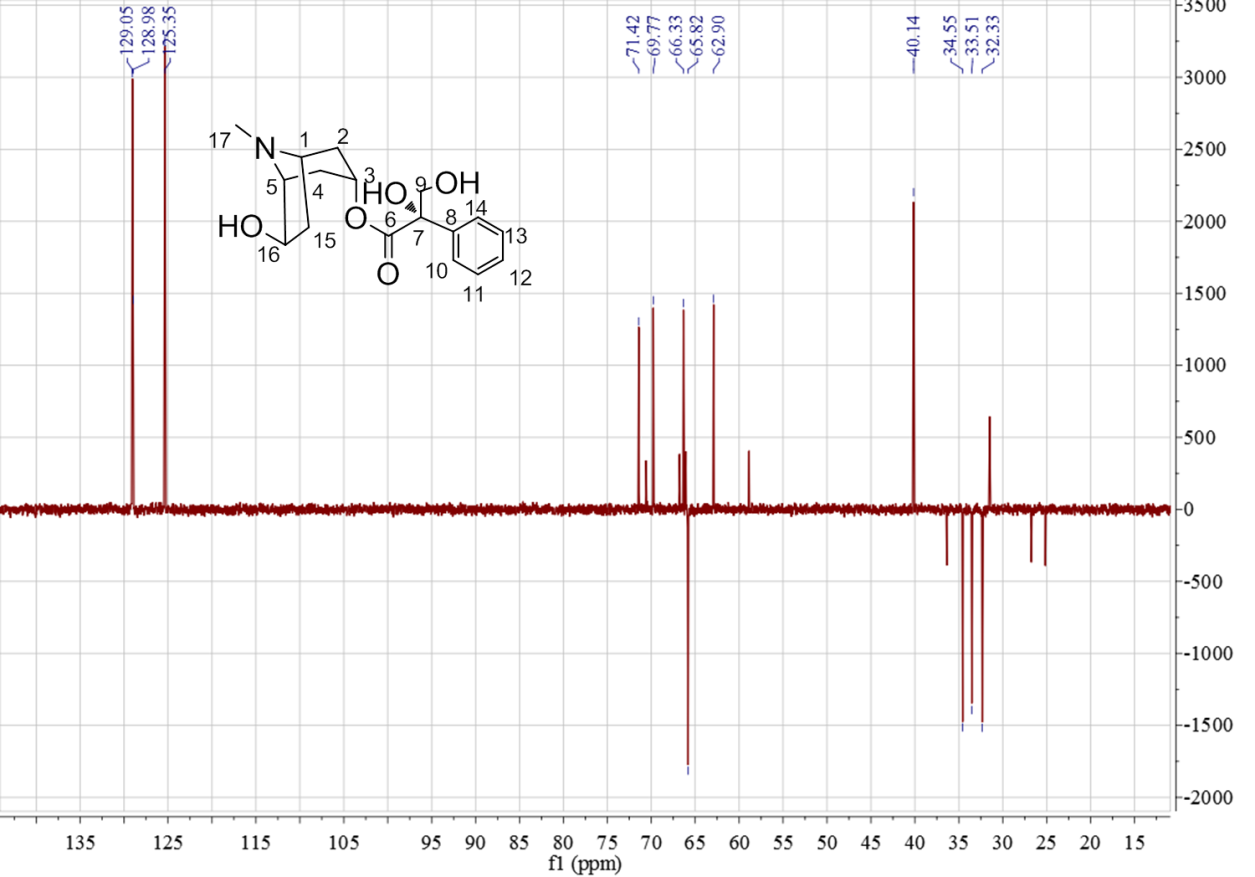


Fig. S8 DEPT 135 spectrum of **6** in D_2_O


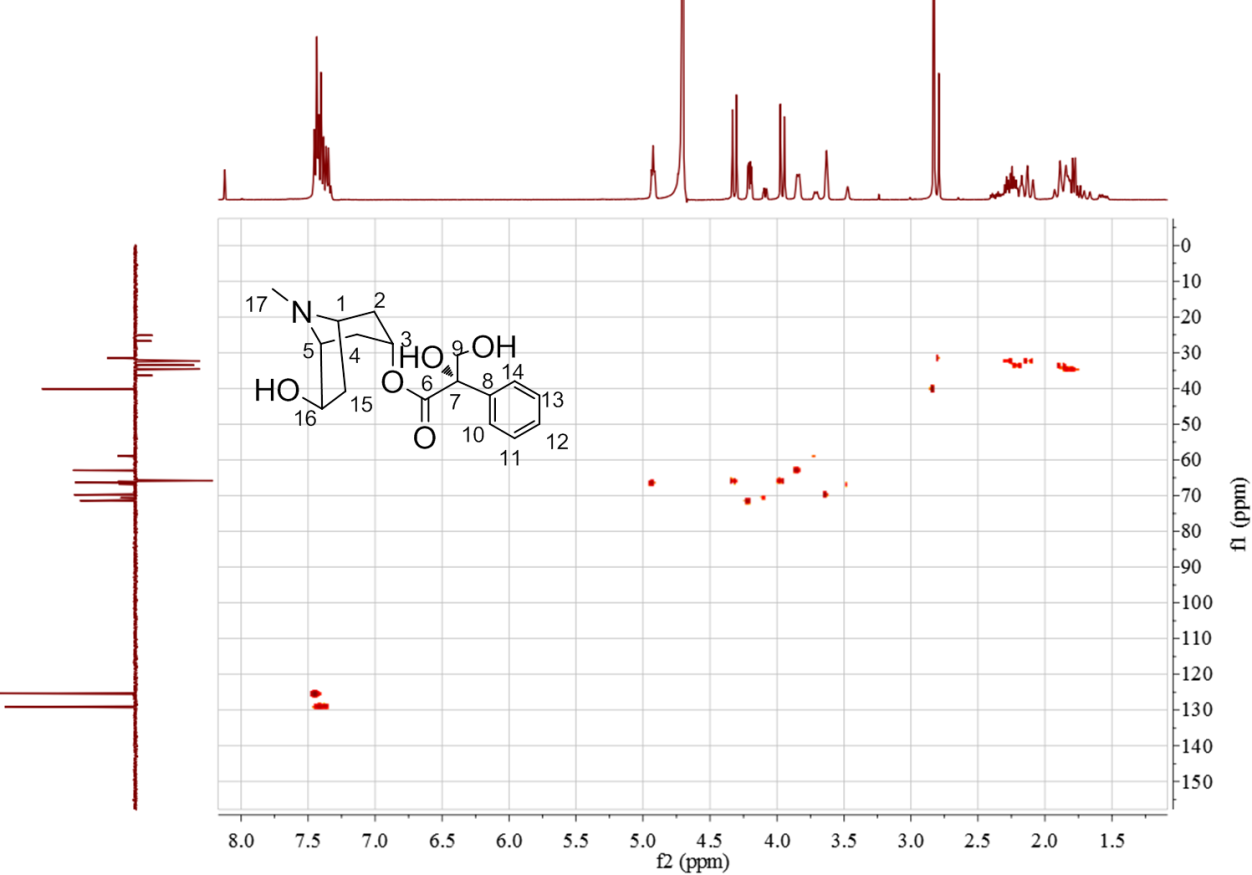


Fig. S9 HSQC spectrum of **6** in D_2_O

Compound **7** was isolated as a white power. UV (H_2_O) λmax (Abs.): 205 (0.1994) nm; [α]25 D-15.789 (H_2_O). The purity and molecular formula were identified in HPLC-ESI-MS. The protonated molecular ion was 306.1354 (calculated for [M+H]^+^ = 306.1263) and molecular formula C_16_H_19_NO_5_. The predominant fragment pattern (306.1387, 142.0846) was in accordance with noranisodine^1^. The NMR spectrum was highly similar to that of anisodine except the lack of methyl group of N-CH_3_. Five stereo-carbons were same as compound **4** (anisodine) and the optical rotation value was in accordance with that of anisodine.The structure was identified as noranisodine with five chiral carbons of the same configurations as anisodine and the assignment was listed below.

^1^H NMR (400 MHz, D_2_O) δ 7.49 – 7.31 (m, 5H, benzene-H), 5.01 (t, J = 4.6 Hz, 1H, 3-H), 4.33 (d, J = 11.7 Hz, 1H, 9-H), 4.00 (s, 1H, 5-H), 3.96 (d, J = 11.7 Hz, 1H, 9-H), 3.89 (s, 1H, 1-H), 3.59 (d, J = 3.2 Hz, 1H, 15-H), 2.85 (d, J = 3.1 Hz, 1H, 16-H), 2.36 – 2.18 (m, 2H, 2-H, 4-H), 2.04 (d, J = 16.8 Hz, 1H, 4-H), 1.87 (d, J = 16.9 Hz, 1H, 2-H).


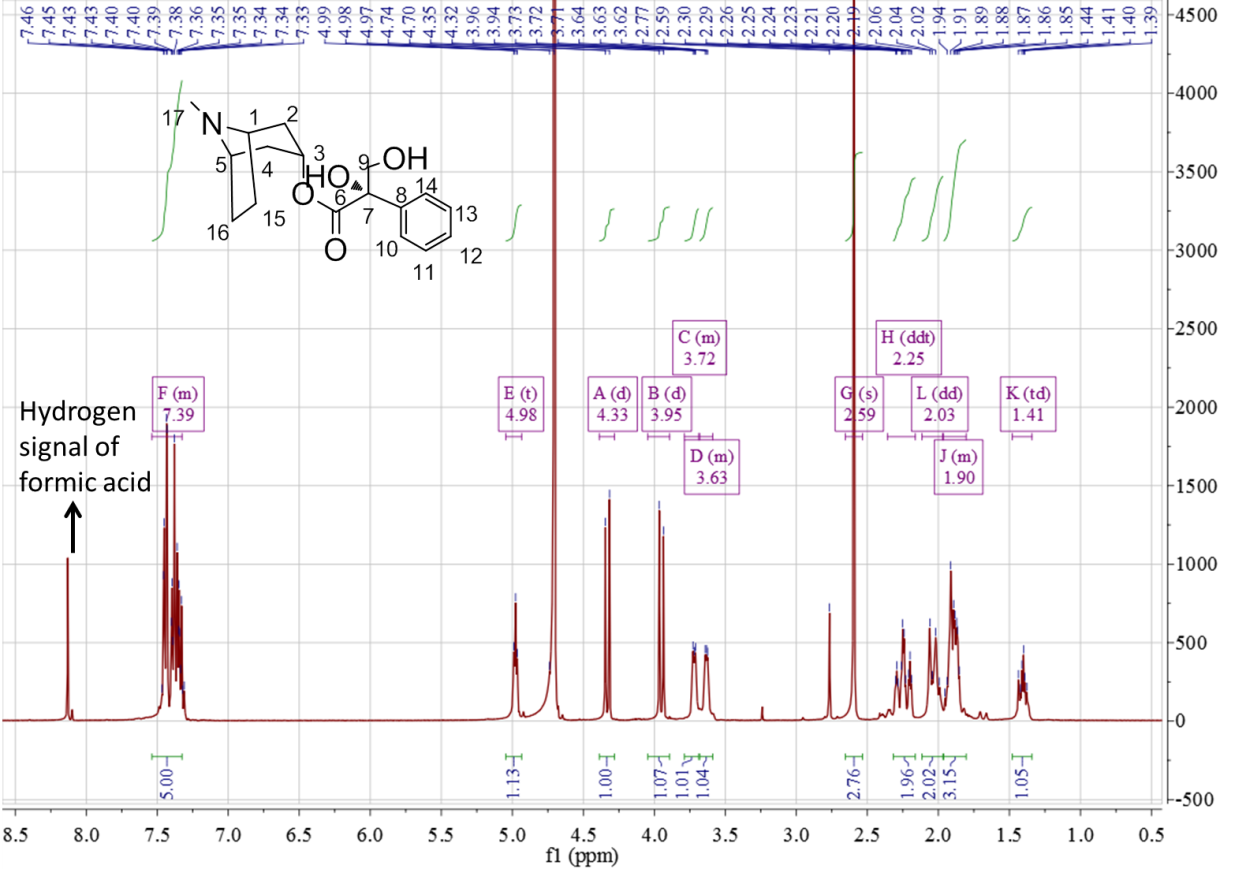


Fig. S10 ^1^H spectrum of **7** in D_2_O

Compound **8** was isolated as a white power. UV (H_2_O) λmax (Abs.): 203 nm (0.2534); IR (KBr) νmax: 3419 (-OH), 1728 (-COO-), 1630, 1599 cm^−1^(benzene) ; [α]25 D -46.640 (H_2_O). The purity and molecular formula were identified in HPLC-ESI-MS. The m/z in positive mode was 466.2107 (calculated for [M+H]^+^ = 466.1999) and molecular formula was C_23_H_31_NO_9_. The predominant fragment ions were 466.2079, 304.1550, 156.1019 and 138.0914, which showed the character of neutral loss of 162 Da (one glycocyl group). The carbon at the chemical shift of 102.93 and its corresponding proton at 4.43 in HSQC spectrum indicated the presence of glucose. Based on COSY and HSQC spectrums, hydrogens linked at glucose were distinguished as 4.43 (1’-H), 3.14 (2’-H), 3.35 (3’-H), 3.27 (4’-H), 3.39 (5’-H), 3.81 (6’-H) and 3.61 (6’-H). The coupling constant (*J* = 8.0 Hz) confirmed the configuration of glucose as β-D-glucose. The ultraviolet spectrum and protons in ^1^H NMR spectrum except β-D-glucose were both in consistent with that of scopolamine while the mass fragment 304.1550 for [M+H]^+^ assisted this point. Therefore, this compound was identified to be glycoscopolamine, a new compound which belonged to tropane alkaloid. Based on the similarity of NMR with scopolamine, the stereo-configurations of five chiral carbon (C-1, C-5, C-7, C-15, C-16) in tropane skeleton were the same as scopolamine (**1**). The detailed NMR assignment was listed as follows.

^1^H NMR (400 MHz, D_2_O) δ 7.36 (d, *J* = 2.7 Hz, 1H, 11-H), 7.34 (d, *J* = 2.2 Hz, 1H, 13-H), 7.32 (dd, *J* = 7.1, 1.7 Hz, 1H, 12-H), 7.31 (d, *J* = 1.9 Hz, 1H, 10-H), 7.29 (d, *J* = 1.4 Hz, 1H, 14-H), 4.98 (t, *J* = 4.9 Hz, 1H, 3-H), 4.43 (d, *J* = 8.0 Hz, 1H, 1’-H), 4.22 (dd, *J* = 10.4, 8.8 Hz, 1H, 9-H), 4.14 (dd, *J* = 10.4, 5.8 Hz, 1H, 9-H), 4.06 (dd, *J* = 8.6, 5.7 Hz, 1H, 7-H), 3.85 (m, 1H, 1-H), 3.81 (dd, *J* = 12.3, 2.2 Hz, 1H, 6’-H), 3.76 (d, *J* = 3.4 Hz, 1H, 15-H), 3.73 (m, 1H, 5-H), 3.61 (dd, *J* = 12.3, 5.8 Hz, 1H, 6’-H), 3.39 (dd, *J* = 8.4, 5.7 Hz, 1H, 5’-H), 3.35 (dd, *J* = 5.7, 3.4 Hz, 1H, 3’-H), 3.27 (dd, *J* = 12.6, 6.2 Hz, 1H, 4’-H), 3.14 (dd, *J* = 9.3, 8.0 Hz, 1H, 2’-H), 3.02 (d, *J* = 3.4 Hz, 1H, 16-H), 2.73 (s, 3H, 17-H), 2.43 – 2.26 (m, 2H, 2-H, 4-H), 2.04 – 1.90 (m, 1H, 2-H), 1.75 (d, *J* = 17.5 Hz, 1H, 4-H).

13C-NMR (500 MHz, D_2_O) δ: 169.25 (C-6), 135.19 (C-8), 129.33 (C-11, C-13), 128.51 (C-12), 128.41 (C-10, C-14), 102.93 (C-1’), 76.06 (C-3’), 75.89 (C-5’), 73.14 (C-2’), 70.20 (C-9), 69.75 (C-4’), 63.97 (C-3), 60.89 (C-6’), 57.01 (C-5), 56.96 (C-1), 53.00 (C-15), 52.70 (C-16), 51.64 (C-7), 30.13 (C-17), 23.67 (C-4), 23.61 (C-6)


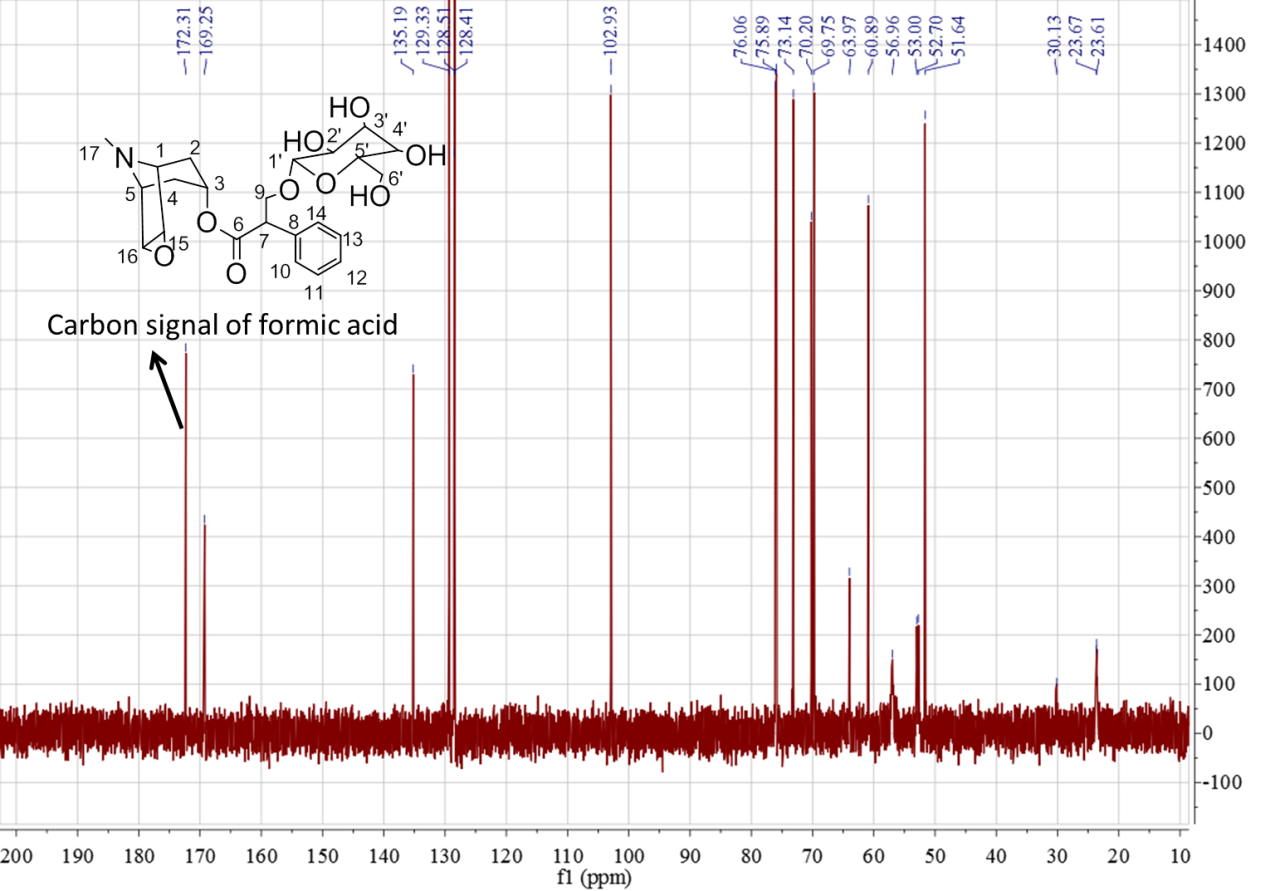


Fig. S11 ^13^C spectrum of **8** in D_2_O.


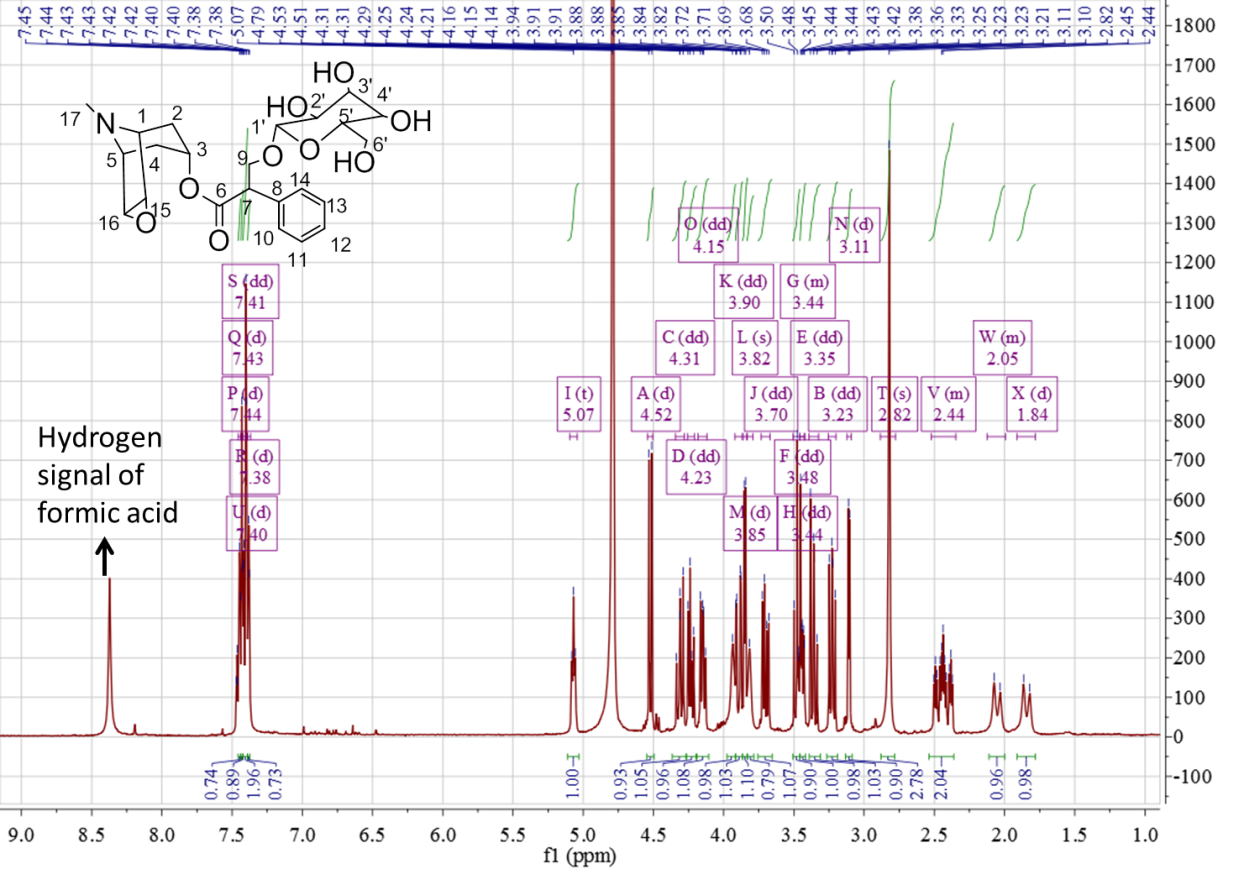


Fig. S12 ^1^H spectrum of **8** in D_2_O.


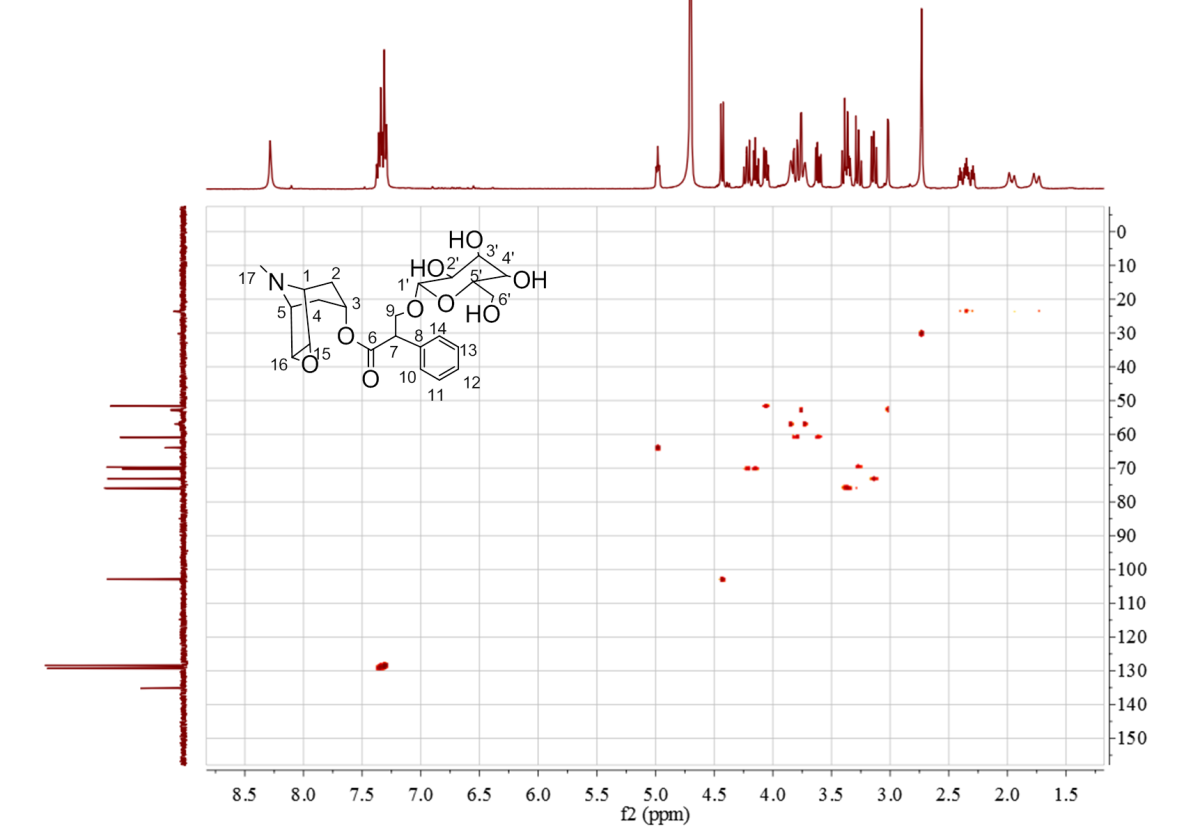


Fig. S13 HSQC spectrum of **8** in D_2_O.


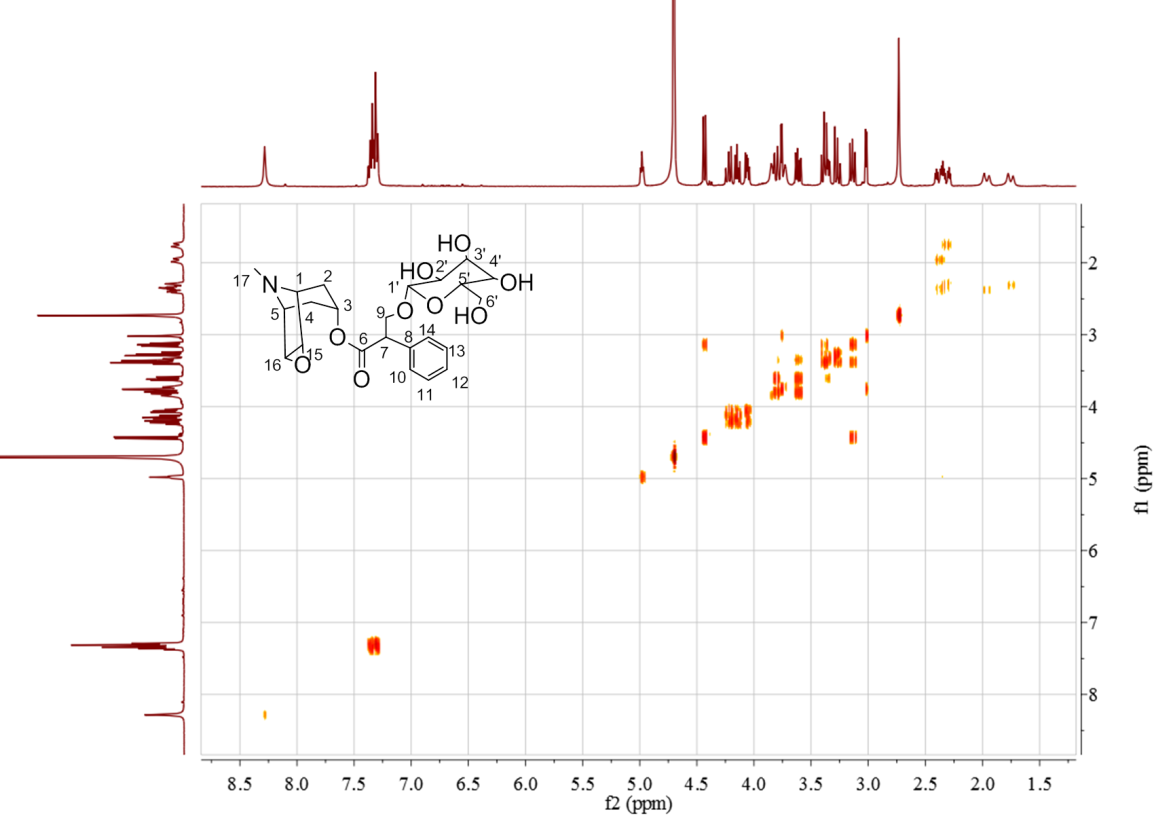


Fig. S14 ^1^H, ^1^H-COSY spectrum of **8** in D_2_O.


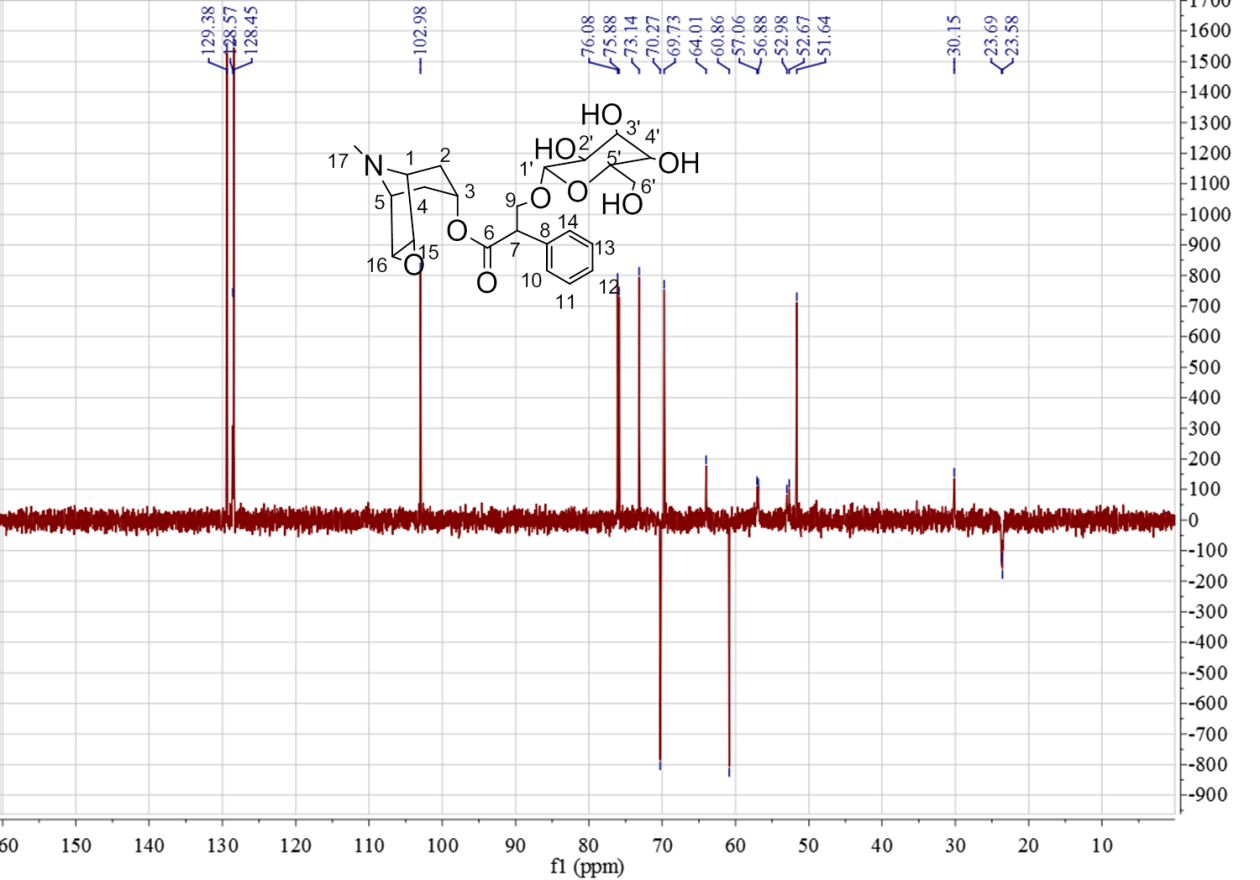


Fig. S15 DEPT 135 spectrum of **8** in D_2_O.


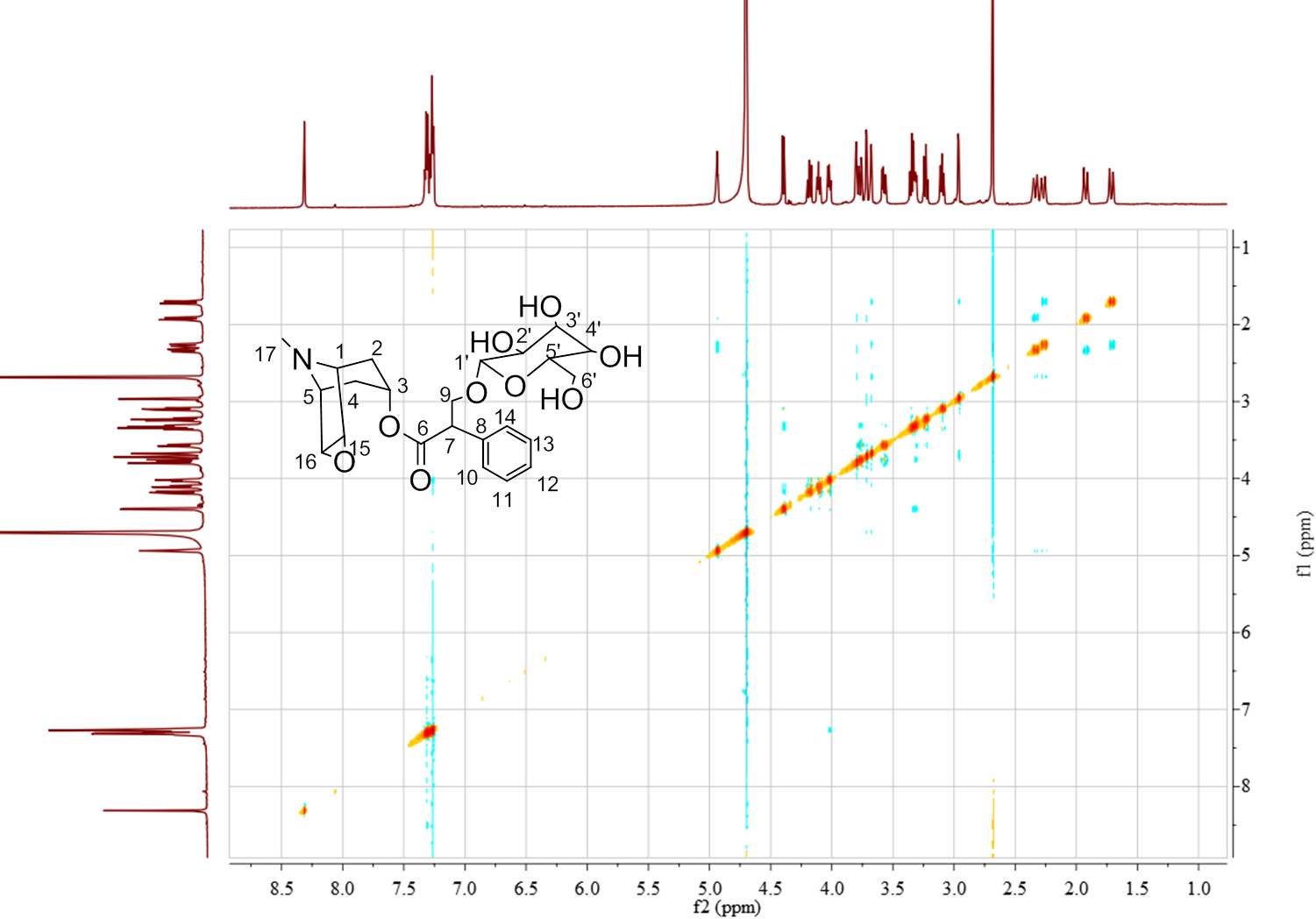


Fig. S16 NOESY spectrum of **8** in D_2_O.


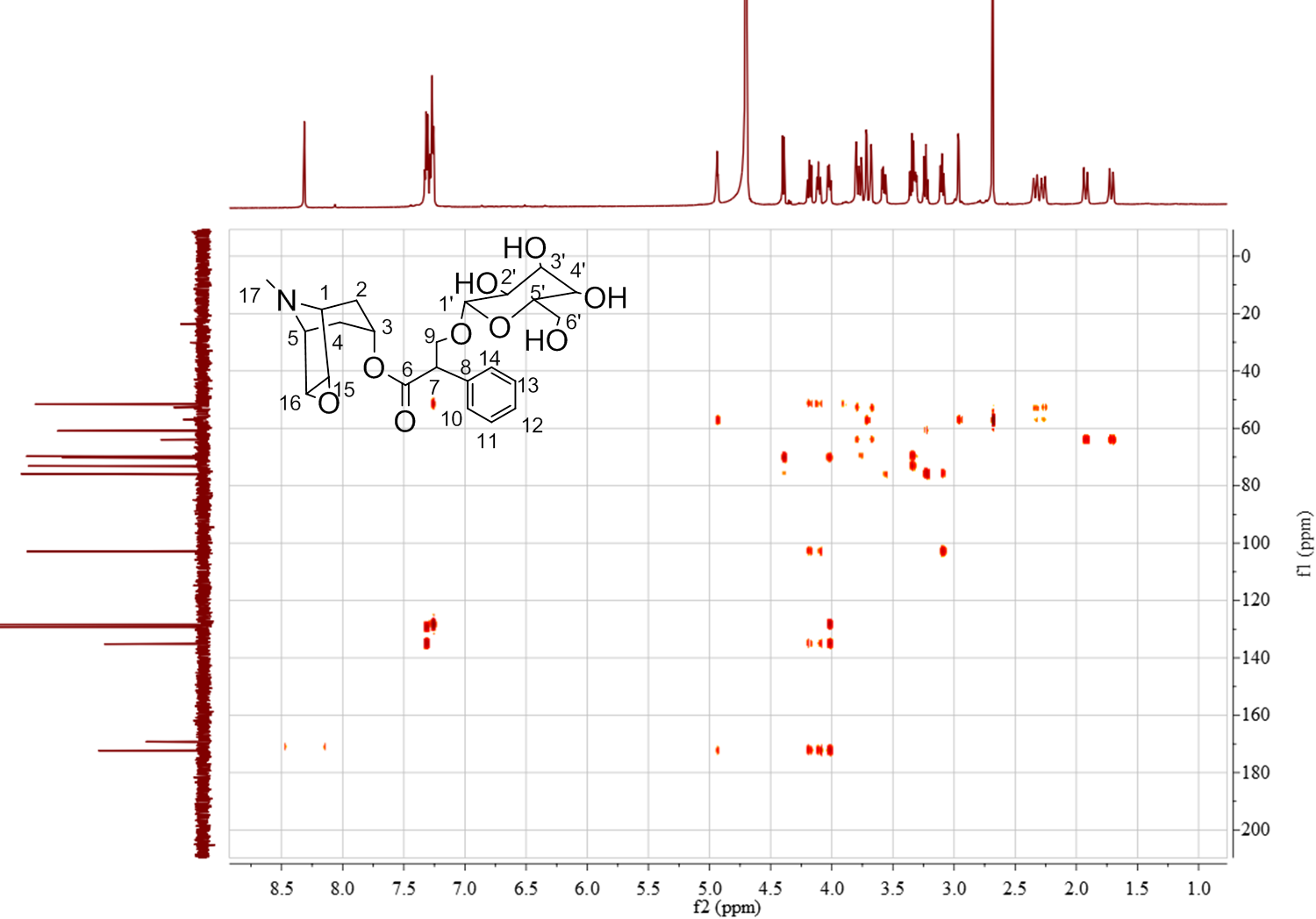


Fig. S17 HMBC spectrum of **8** in D_2_O.

Compound **9** was isolated as a white power. UV (H_2_O) λmax (Abs.): 203 nm (0.2290); IR (KBr) νmax: 3423 (-OH), 2956 (benzene C-H), 1730 (-COO-), 1632 cm^−1^(benzene) ; [α]25 D -27.778 (H_2_O). The purity and molecular formula were identified in HPLC-ESI-MS. The m/z in positive mode was 292.1599 (calculated for [M+H]^+^ = 292.1471) and molecular formula was C_16_H_21_NO_4_. The predominant fragment ions were 292.1540 and 126.0910, which was in accordance with noranisodamine^2^. The NMR information was of high similarity to anisodamine except lack of three protons for N-CH_3_. Five stereo-carbons were same as compound **2** (anisodamine) while the optical rotation value was in accordance with that of anisodamine.Therefore, it was identified as noranisodamine with five chiral carbons of the same configurations with anisodamine and the assignment was as follows.

^1^H NMR (400 MHz, D_2_O) δ 7.33 (ddd, J = 10.3, 5.8, 2.6 Hz, 5H, benzene-H), 4.94 (d, J = 4.1 Hz, 1H, 3-H), 4.28 (dd, J = 4.7, 2.1 Hz, 1H, 16-H), 4.11 (ddd, J = 14.8, 10.4, 4.7 Hz, 1H, 9-H), 3.89 (dt, J = 11.6, 6.9 Hz, 3H, 9-H, 1-H, 5-H), 3.69 (s, 1H, 7-H), 2.28 – 1.95 (m, 3H, 15-H, 15-H, 4-H), 1.82 (ddd, J = 44.8, 27.4, 11.6 Hz, 2H, 2-H, 4-H), 1.52 (dd, J = 12.7, 5.7 Hz, 1H, 2-H).

DEPT 135 (400 MHz, D_2_O) δ 129.19 (C-11, C-13), 128.23 (C-10, C-12, C-14), 70.97 (C-16), 65.86 (C-3), 62.15 (C-7), 61.85 (C-9), 54.08 (C-5), 53.46 (C-1), 36.50 (C-2), 31.80 (C-4), 30.48 (C-15).

**
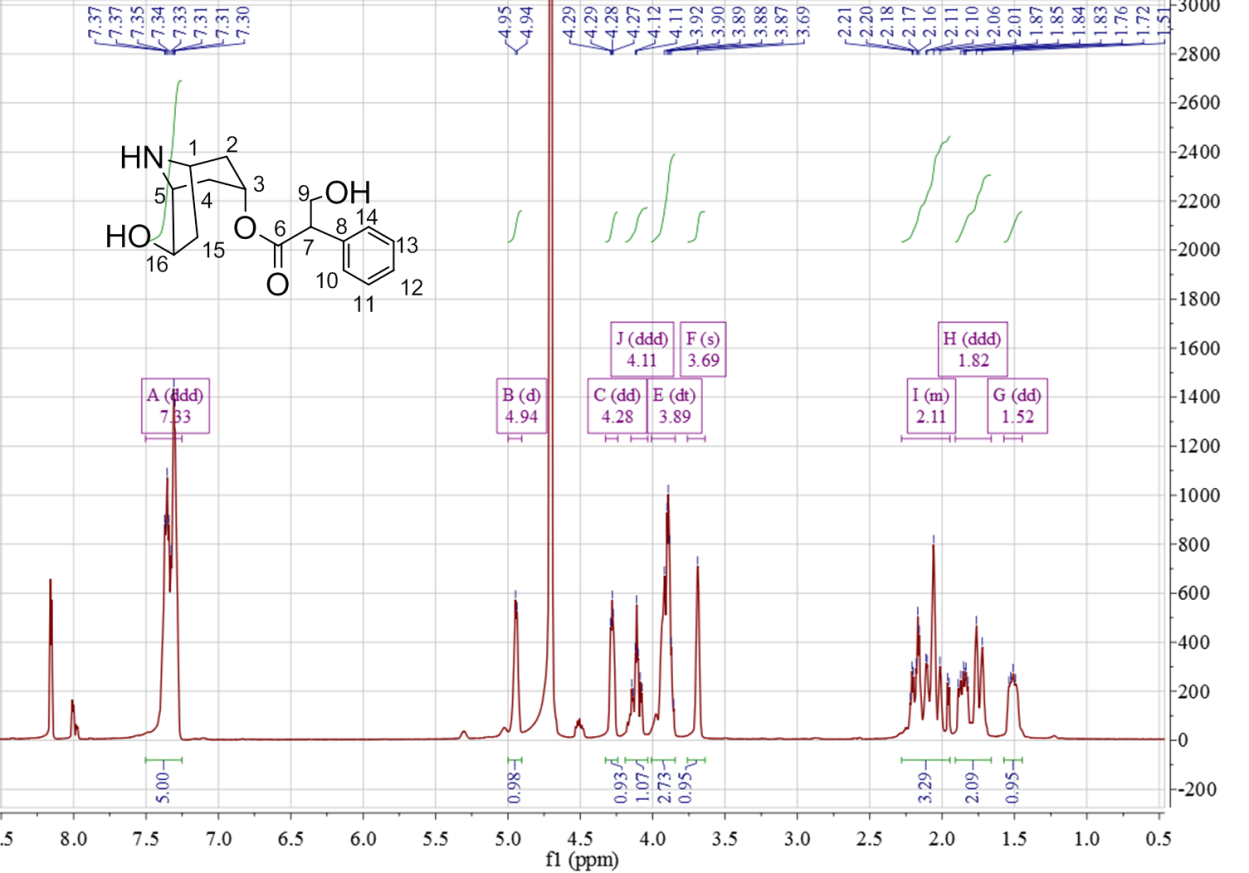
**

Fig. S18 ^1^H spectrum of **9** in D_2_O.


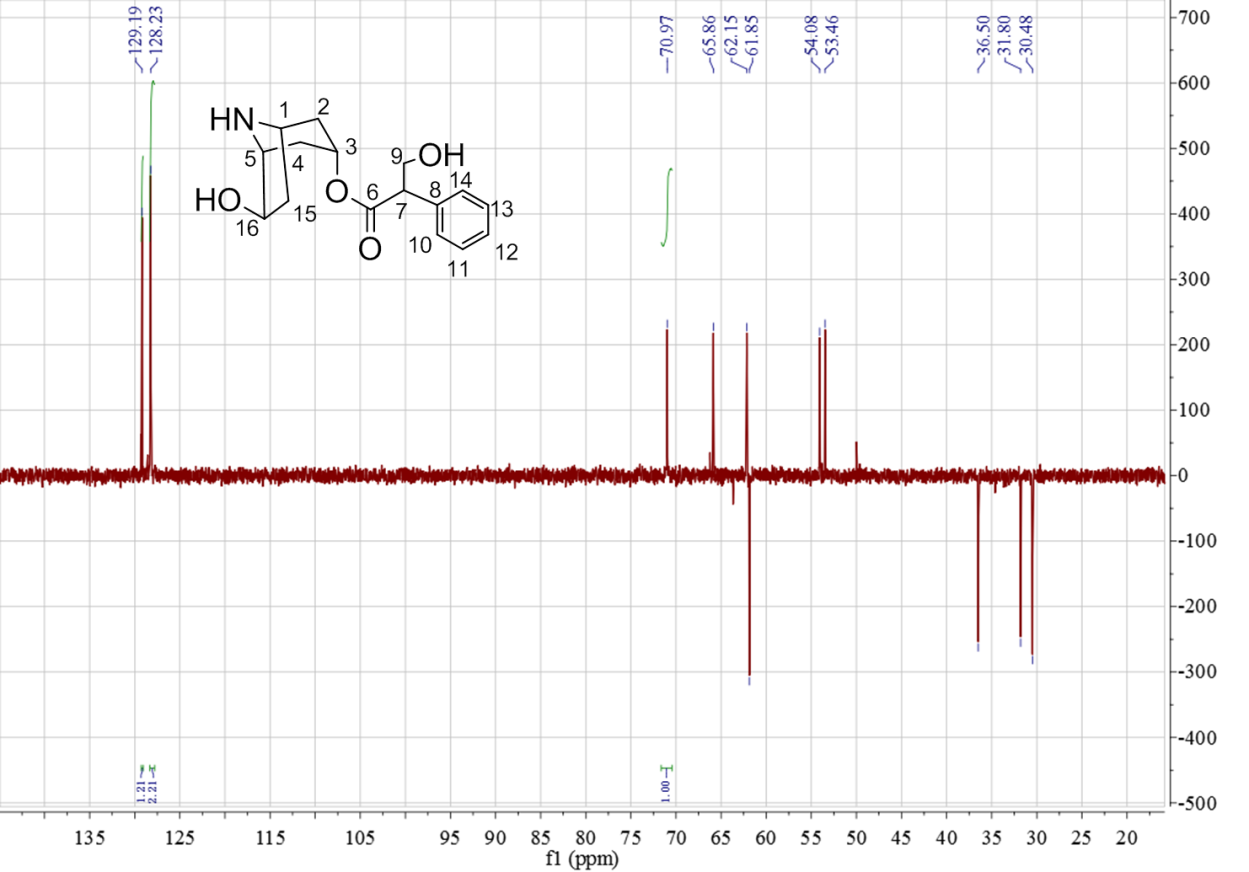


Fig. S19 DEPT 135 spectrum of **9** in D_2_O.


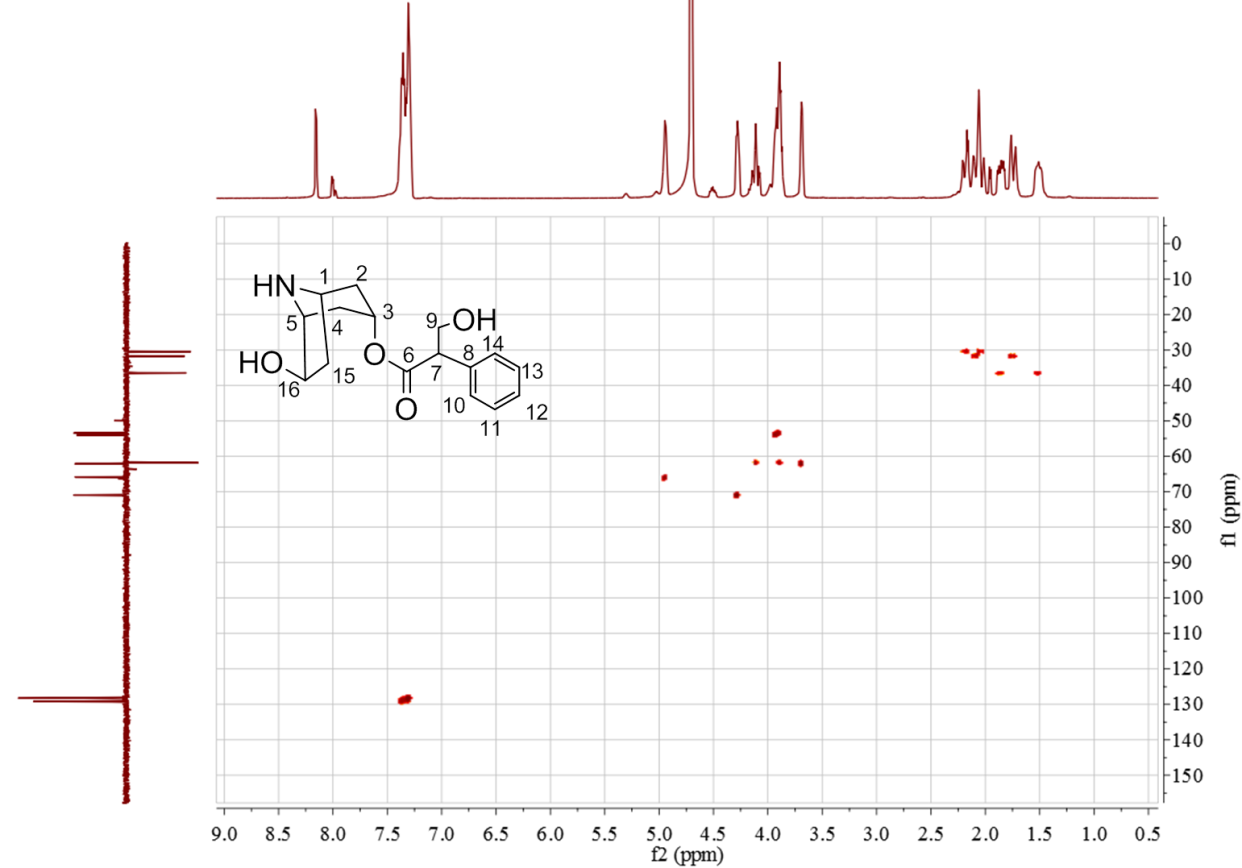


Fig. S20 HSQC spectrum of **9** in D_2_O.

Compound **10** was isolated as a white power. UV (H_2_O) λmax (Abs.): 203.6 nm (0.2136); IR (KBr) νmax: 3427 (-OH), 1734 (-COO-), 1638 cm^−1^(benzene) ; [α]25 D -26.23 (H_2_O). The purity and molecular formula were identified in HPLC-ESI-MS. The m/z in positive mode was 306.1787 (calculated for [M+H]^+^ = 306.1627) and molecular formula was C_17_H_23_NO_4_. The predominant fragment ions were 306.1706, 142.1226 and 124.1120, results of hydrolysis of ester group and loss of H_2_O successively, which was in accordance with Deepoxyanisodine^3^. The NMR spectrum was in the pattern of hyoscyamine and at the same time the lack of proton signal at C-7 and presence of methyl group at C-17 confirmed this compounds as Deepoxyanisodine. Three stereo-carbons (C-1, C-5, C-7) were similar to hyoscyamine and anisodine and the optical rotation value was also in accordance. Thus, the stereo-configurations were R,S,S for C-1, C-5, C-7 respectively. The specific assignment of NMR was as follows.

^1^H NMR (400 MHz, D_2_O) δ 7.54 – 7.33 (m, 5H, benzene-H), 4.98 (t, *J* = 4.8 Hz, 1H, 3-H), 4.33 (d, *J* = 11.7 Hz, 1H, 9-H), 3.95 (d, *J* = 11.7 Hz, 1H, 9-H), 3.79 – 3.69 (m, 1H, 5-H), 3.68 – 3.59 (m, 1H, 1-H), 2.59 (s, 3H, 17-H), 2.25 (ddt, *J* = 20.8, 16.8, 3.8 Hz, 2H, 2-H, 4-H), 2.03 (dd, *J* = 18.5, 8.7 Hz, 2H, 2-H, 15-H), 1.97 – 1.80 (m, 3H, 15-H, 4-H, 16-H), 1.41 (td, *J* = 9.4, 5.5 Hz, 1H, 16-H).

DEPT 135 (400 MHz, D_2_O) δ 128.98 (C-11, C-13), 128.90 (C-12), 125.43 (C-10, C-14), 66.68 (C-3), 65.99 (C-9), 62.04 (C-5), 61.95 (C-1), 38.42 (C-17), 34.25 (C-4), 34.18 (C-2), 23.11 (C-15), 22.84 (C-16).


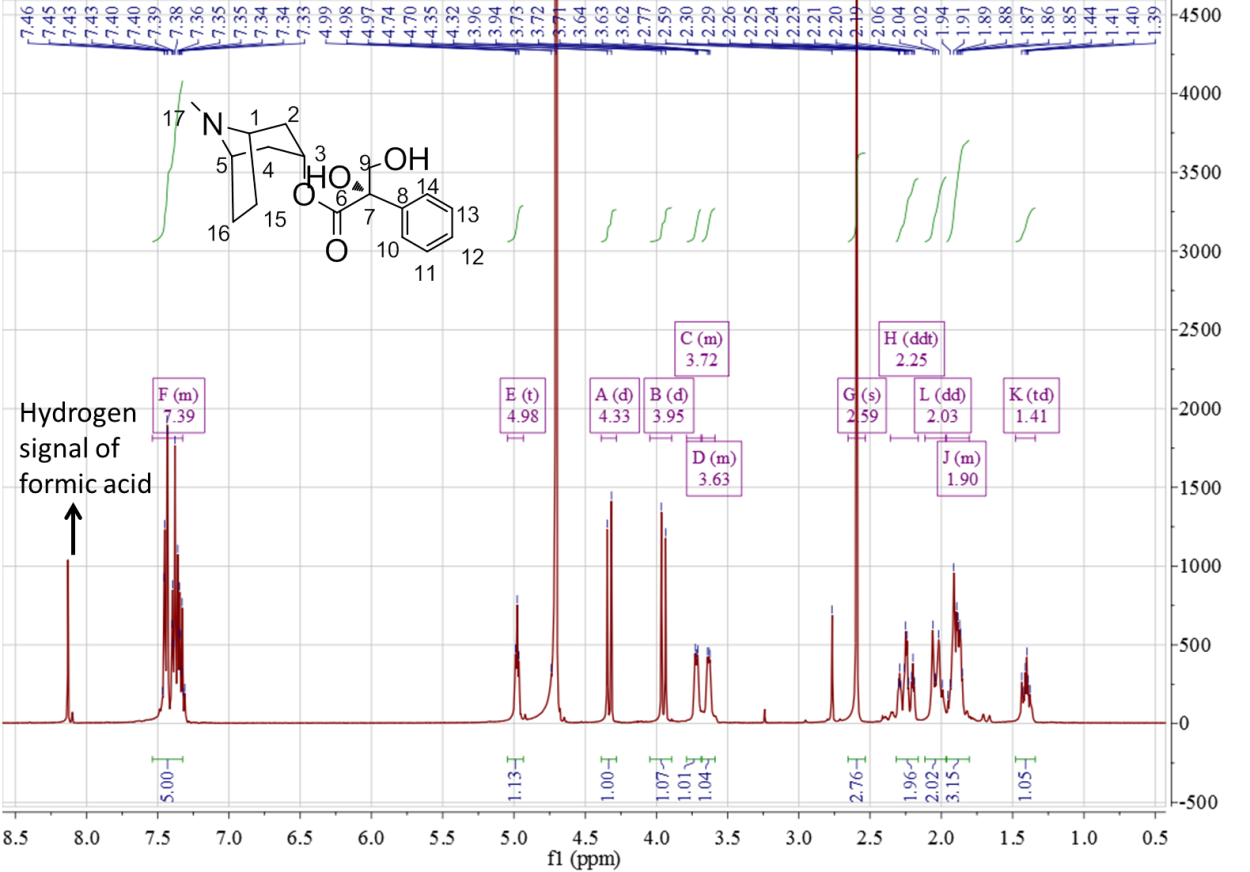


Fig. S21 ^1^H spectrum of **10** in D_2_O.


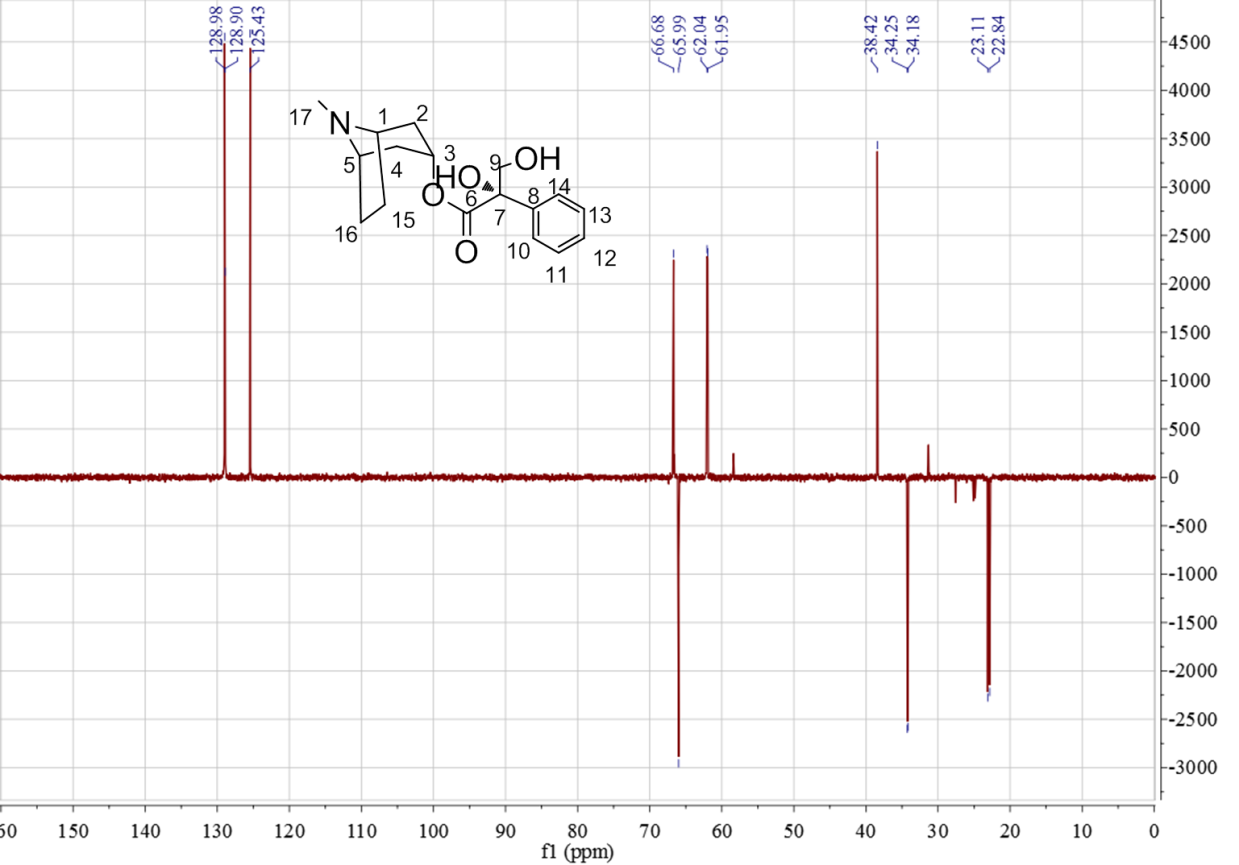


Fig. S22 DEPT 135 spectrum of **10** in D_2_O.


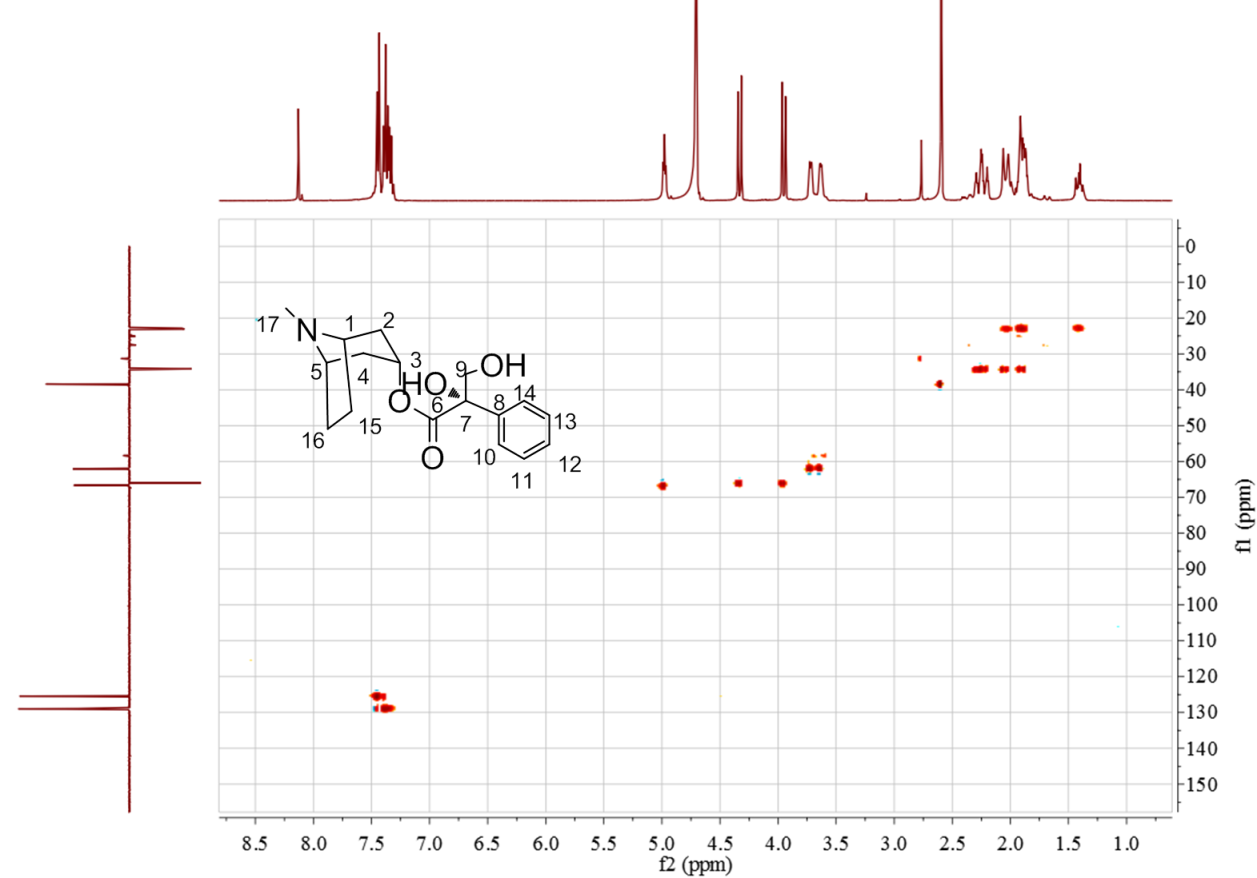


Fig. S23 HSQC spectrum of **10** in D_2_O.

Compound **11** was isolated as a white power. UV (H_2_O) λmax (Abs.): 203.6 nm (0.2136); IR (KBr) νmax: 3427 (-OH), 1734 (-COO-), 1638 cm^−1^(benzene) ; [α]25 D -26.23 (H_2_O). The purity and molecular formula were identified in HPLC-ESI-MS. The m/z in positive mode was 276.1624 (calculated for [M+H]^+^ = 276.1521) and molecular formula was C_16_H_21_NO_3_. The predominant fragment ions were 276.1599, 121.0646, 110.0964 and 93.0700, which was in consistent with norhyoscyamine^3^. The NMR spectrum was highly similar to that of hyoscyamine except the lack of methyl group at N-CH_3_. The optical rotation of this compound, [α]25 D, was detected as -29.651. Assisted by MS and NMR information, it was identified as norhyoscyamine with three chiral carbons of the same configurations as hyoscyamine (**3**). The NMR assignment was as follows.

DEPT 135 (400 MHz, D_2_O) δ 129.30 (C-11, C-13), 128.42 (C-10, C-14), 128.32 (C-12), 66.57 (C-3), 62.29 (C-9), 53.84 (C-5), 53.75 (C-1), 53.74 (C-7), 33.00 (C-4), 32.81 (C-2), 25.31 (C-15), 25.10 (C-16).

^1^H NMR (400 MHz, D_2_O) δ 7.38 – 7.24 (m, 5H, benzene-H), 4.98 (t, J = 4.6 Hz, 1H, 3-H), 4.13 – 4.03 (m, 1H, 9-H), 3.94 – 3.83 (m, 3H, 9-H, 5-H, 7-H), 3.81 (dd, J = 8.4, 4.7 Hz, 1H, 1-H), 2.23 – 2.14 (m, 1H, 4-H), 2.11 (dd, J = 17.1, 4.4 Hz, 1H, 2-H), 2.03 – 1.97 (m, 1H, 15-H), 1.95 (d, J = 9.5 Hz, 1H, 4-H), 1.92 – 1.80 (m, 1H, 15-H), 1.77 (d, J = 16.6 Hz, 1H, 2-H), 1.71 (dd, J = 12.7, 6.5 Hz, 1H,16-H), 1.45 (ddd, J = 14.0, 10.0, 4.4 Hz, 1H, 16-H).


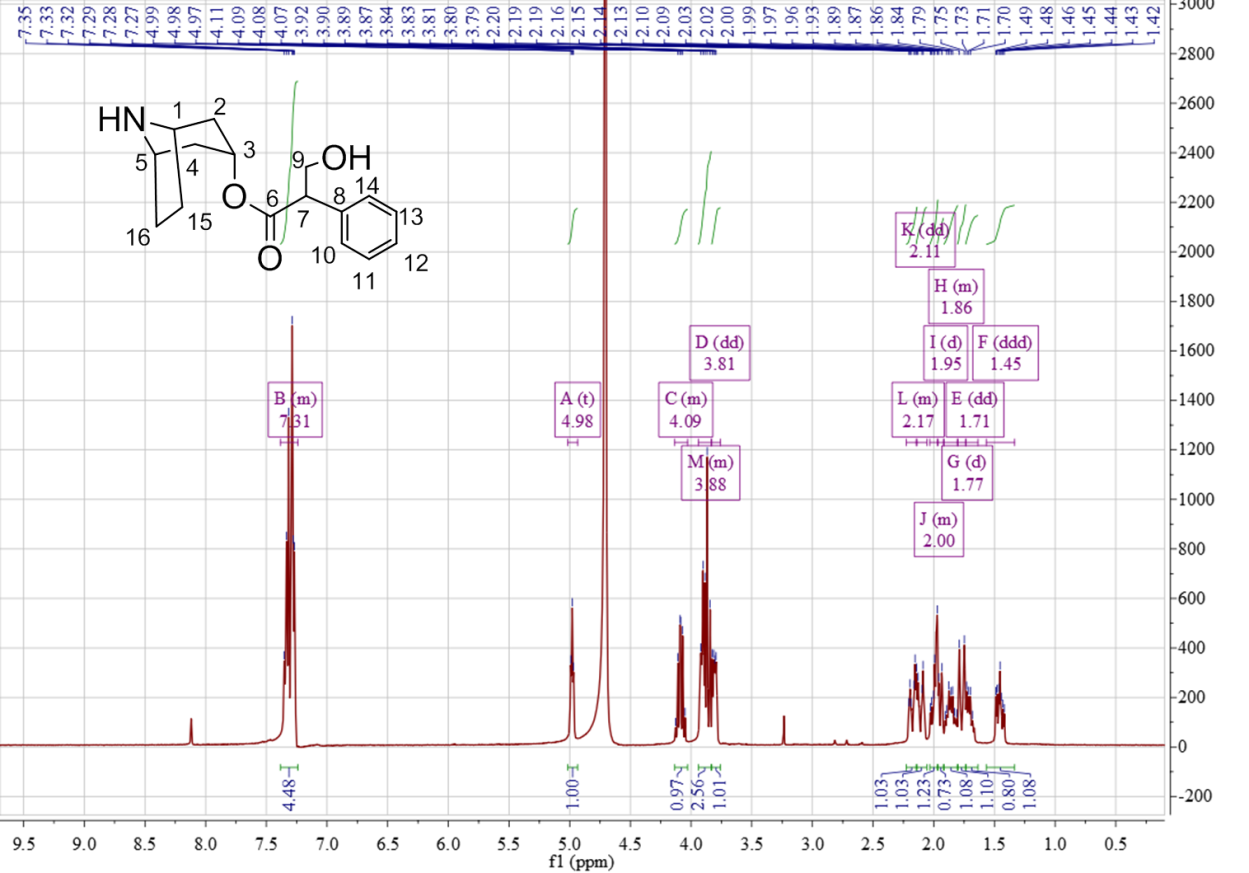


Fig. S24 ^1^H spectrum of **11** in D_2_O.

**
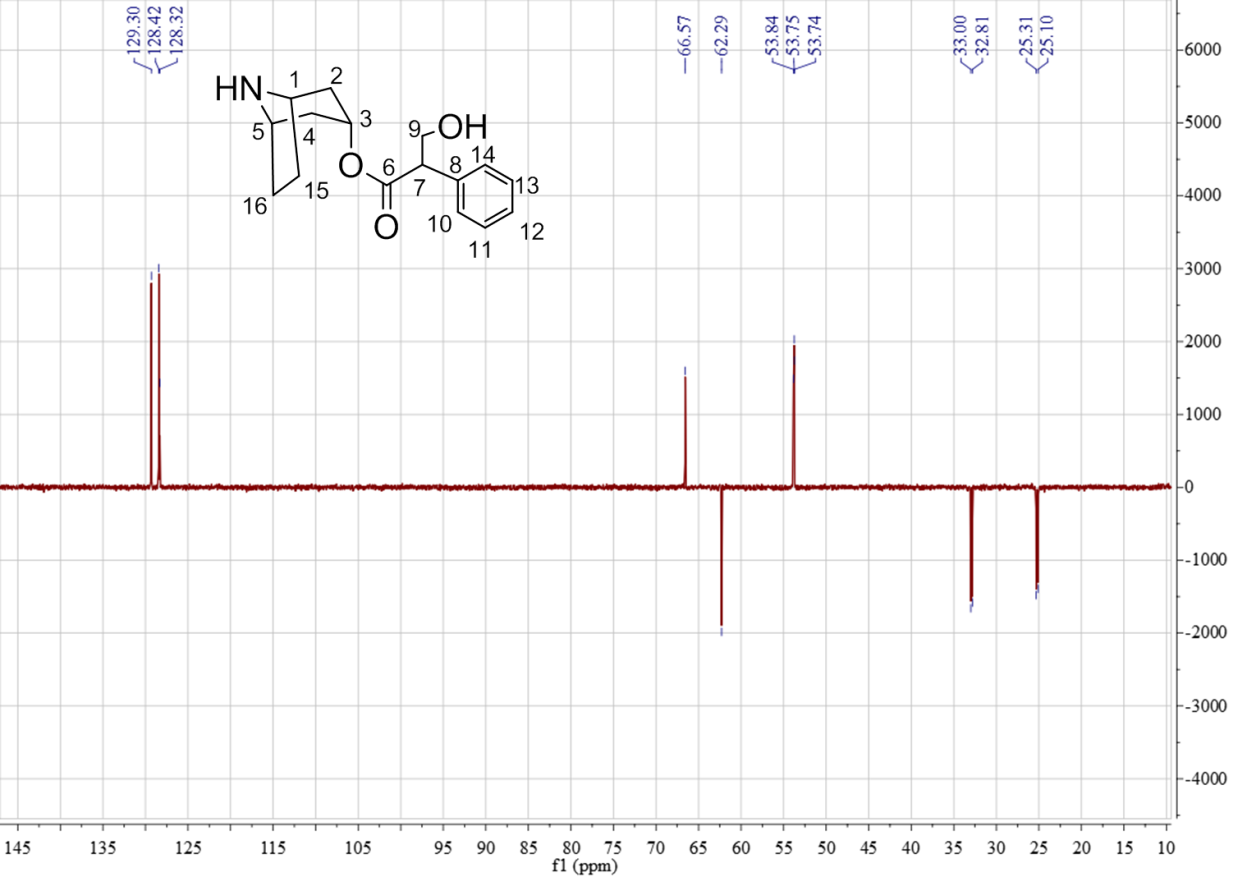
**

Fig. S25 DEPT 135 spectrum of **11** in D_2_O.

**
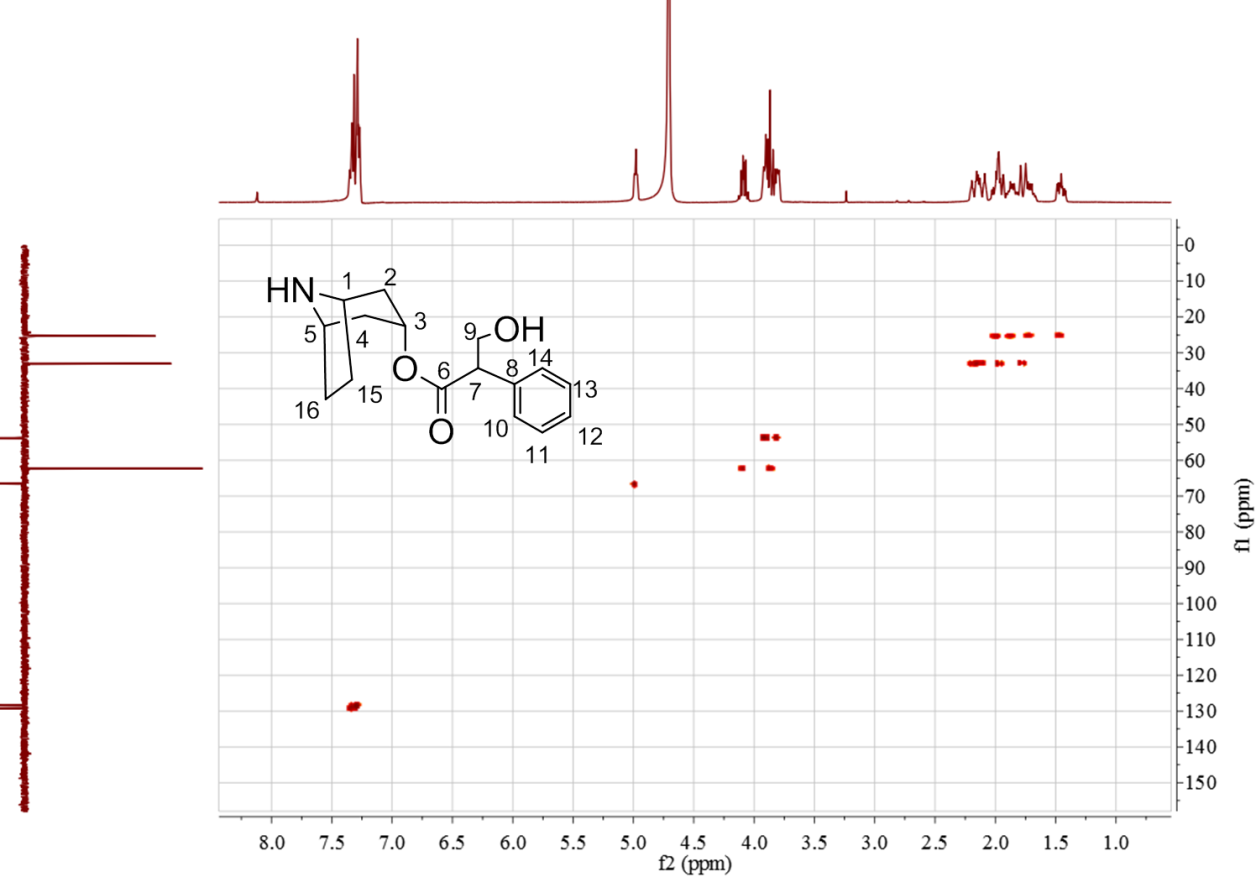
**

Fig. S26 HSQC spectrum of **11** in D_2_O.

**
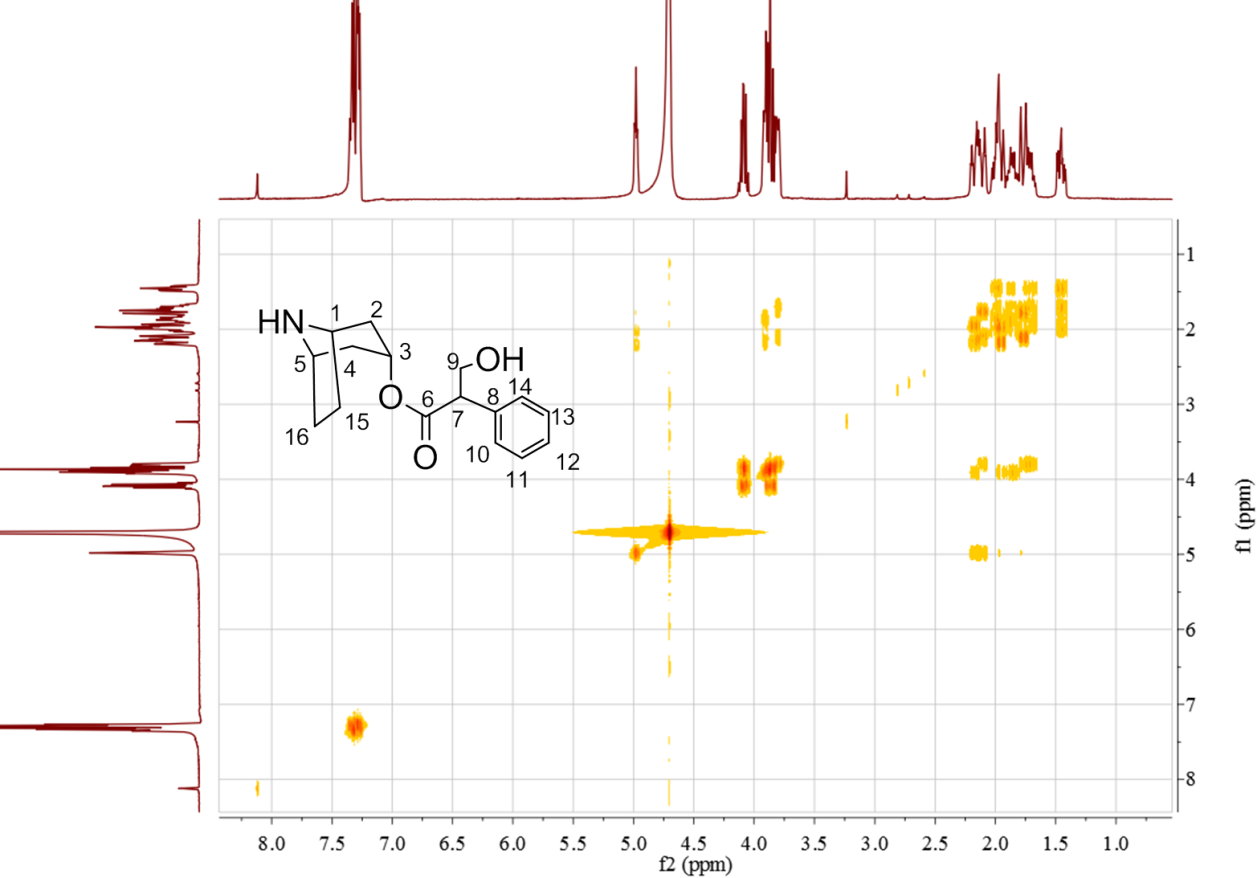
**

Fig. S27 ^1^H, ^1^H-COSY spectrum of **11** in D_2_O.

Compound **12** was isolated as a white power. UV (H_2_O) λmax (Abs.): 203 nm (0.2423); IR (KBr) νmax: 3423 (-OH), 2927 (benzene C-H), 1730 (-COO-), 1631, 1596 cm^−1^(benzene C=C) ; [α]25 D -46.774 (H_2_O). The purity and molecular formula were identified in HPLC-ESI-MS. The m/z in positive mode was 438.2395 (calculated for [M+H]^+^ = 438.2050) and molecular formula was C_22_H_31_NO_8_. The predominant fragment ions were 438.2126 and 276.1591, result by neutral loss of 162 Da (one glycocyl group). The carbon at the chemical shift of 102.94 and its corresponding proton at 4.44 in HSQC spectrum indicated the presence of glucose. Based on COSY and HSQC spectrums, hydrogens linked at glucose were distinguished as 4.44 (1’-H), 3.15 (2’-H), 3.37 (3’-H), 3.27 (4’-H), 3.37 (5’-H), 3.81 (6’-H) and 3.62 (6’-H). The coupling constant (*J* = 8.0 Hz) confirmed the configuration of glucose as β-D-glucose. The ultraviolet spectrum and protons in ^1^H NMR spectrum except β-D-glucose were both in consistent with that of norhyoscyamine while the mass fragment 276.1591 for [M+H]^+^ assisted this point. Therefore, this compound was identified to be glyconorhyoscyamine, a new compound which belonged to tropane alkaloid. The stereo-configuration of three chiral carbon (C-1, C-5, C-7) in tropane skeleton were the same as norhyoscyamine (**11**).The NMR information was assigned as follows.

^1^H NMR (400 MHz, D_2_O) δ 7.33 (pd, *J* = 6.9, 2.0 Hz, 5H, benzene-H), 5.00 (t, *J* = 4.5 Hz, 1H, 3-H), 4.44 (d, *J* = 8.0 Hz, 1H, 1’-H), 4.23 (m, 1H, 9-H), 4.13 (dd, *J* = 10.0, 5.9 Hz, 1H, 9-H), 4.09 (dd, *J* = 8.6, 5.8 Hz, 1H, 7-H), 3.92 (dd, *J* = 11.2, 8.2 Hz, 1H, 5-H), 3.84 – 3.77 (m, 2H, 1-H, 6’-H), 3.62 (dd, *J* = 12.3, 5.8 Hz, 1H, 6’-H), 3.37 (dd, *J* = 8.4, 3.1 Hz, 1H, 3’-H), 3.37 (ddd, *J* = 9.4, 8.1, 5.6 Hz, 1H, 5’-H), 3.27 (dd, *J* = 12.5, 6.3 Hz, 1H, 4’-H), 3.15 (dd, *J* = 15.0, 6.9 Hz, 1H, 2’-H), 2.23 – 2.15 (m, 1H, 4-H), 2.13 (dd, *J* = 12.8, 3.6 Hz, 1H, 2-H), 2.08 – 2.02 (m, 1H, 15-H), 1.98 (d, *J* = 15.1 Hz, 1H, 4-H), 1.89 (dt, *J* = 12.0, 5.5 Hz, 1H, 15-H), 1.84 – 1.75 (m, 1H, 2-H), 1.75 – 1.67 (m, 1H, 16-H), 1.51 – 1.42 (m, 1H, 16-H).

^13^C NMR (500 MHz, D_2_O) δ 169.53 (C-6), 134.94 (C-8), 129.32 (C-11), 128.44 (C-12), 128.38 (C-10, C-14), 102.94 (C-1’), 76.04 (C-3’), 75.89 (C-5’), 73.14 (C-2’), 70.42 (C-9), 69.76 (C-4’), 66.73 (C-3), 60.90 (C-6’), 53.86 (C-5), 53.75 (C-1), 51.84 (C-7), 32.96 (C-4), 32.81 (C-2), 25.29 (C-15), 25.10 (C-16)


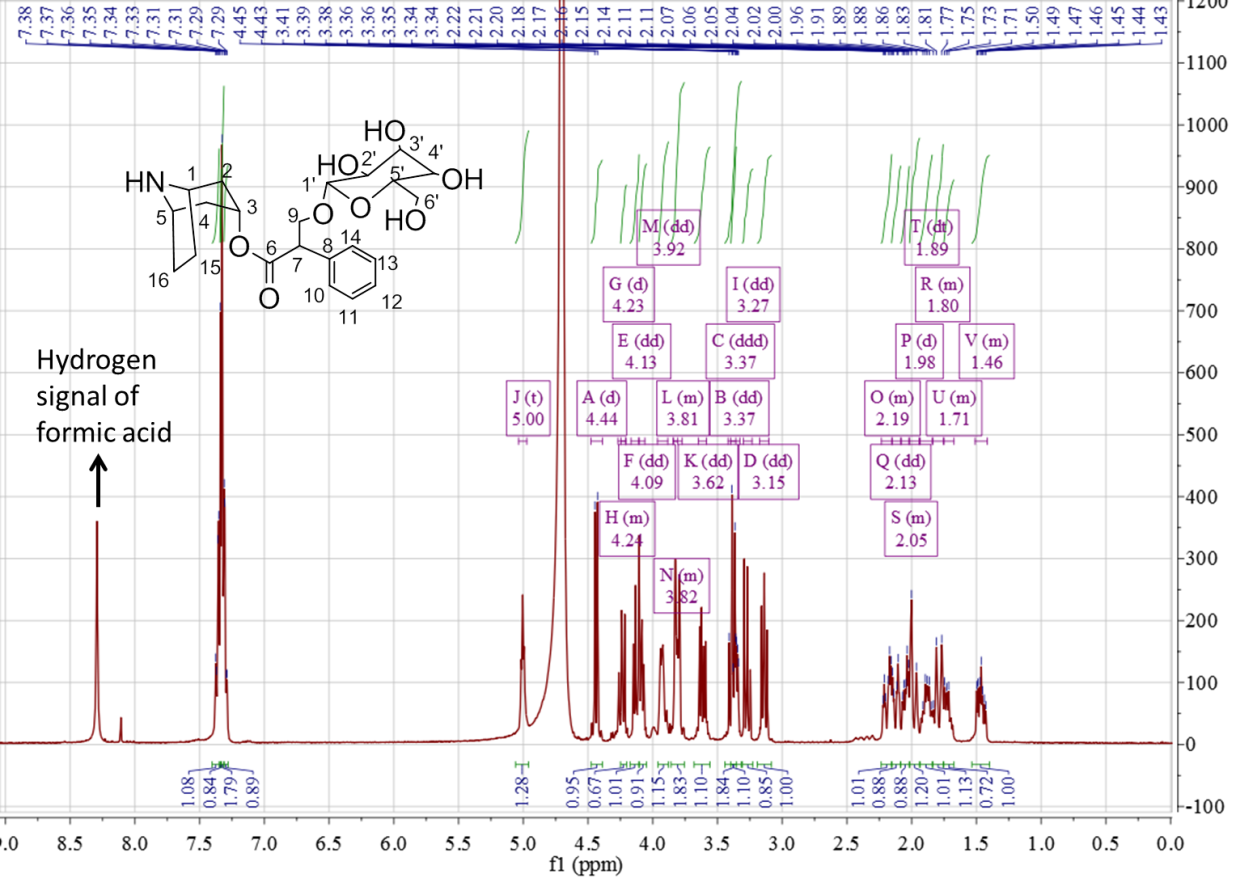


Fig. S28 ^1^H spectrum of **12** in D_2_O.


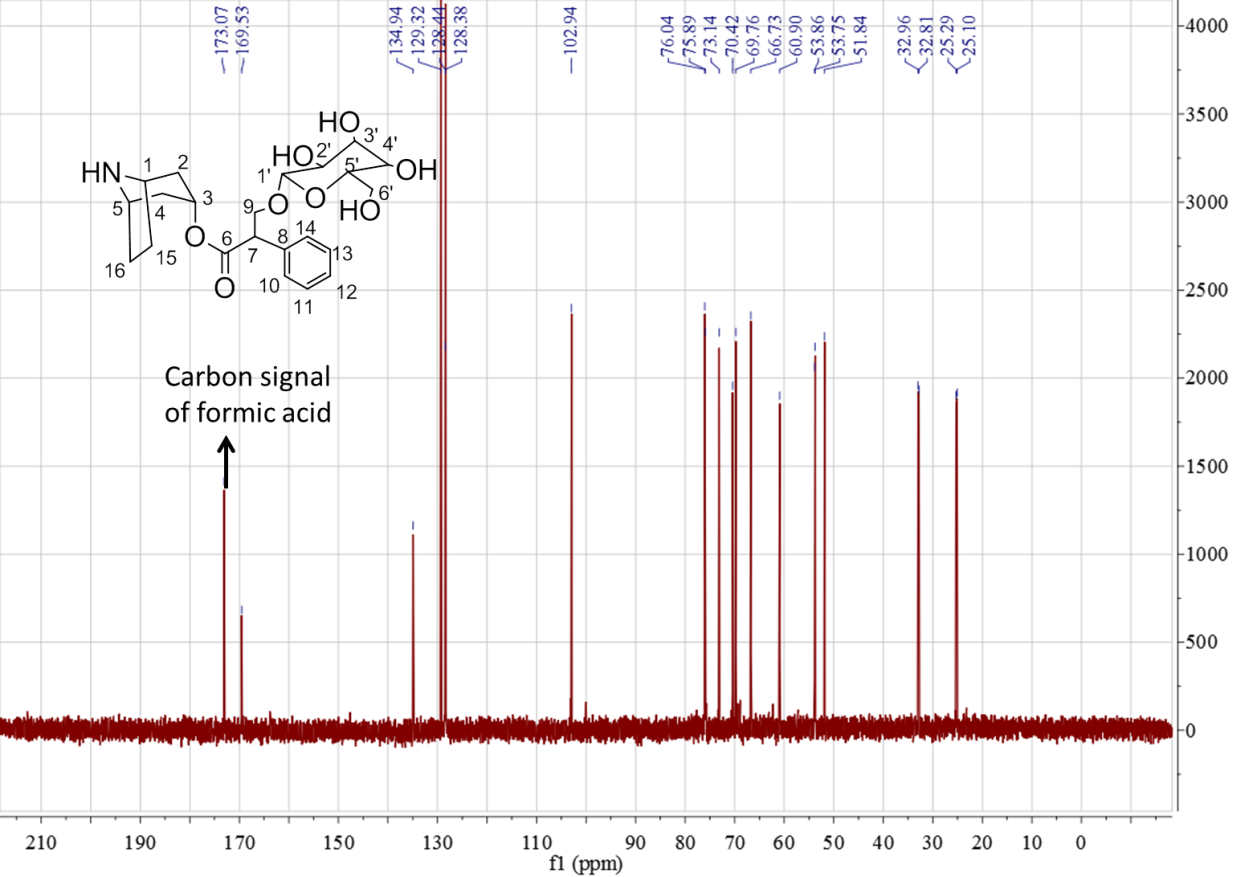


Fig. S29 ^13^C spectrum of **12** in D_2_O.


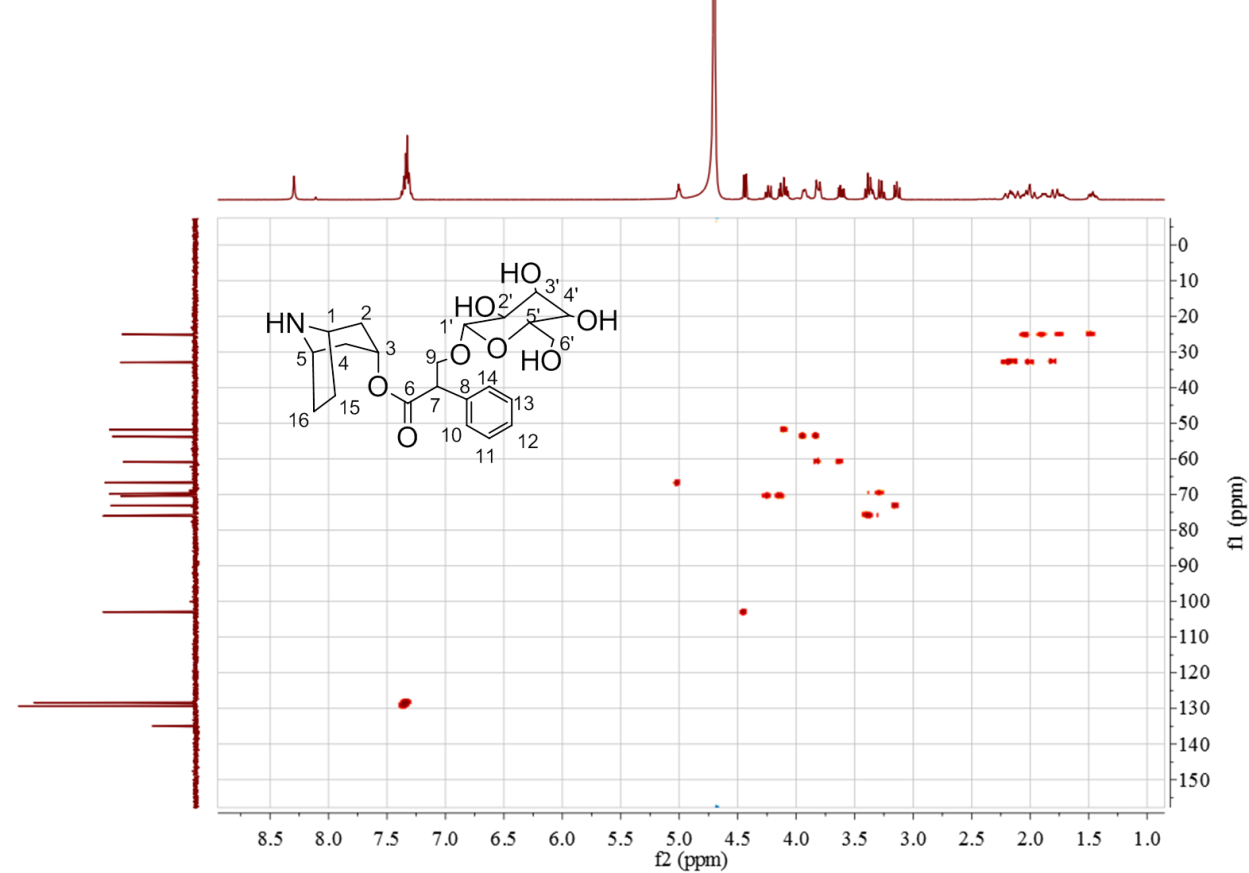


Fig. S30 HSQC spectrum of **12** in D_2_O.


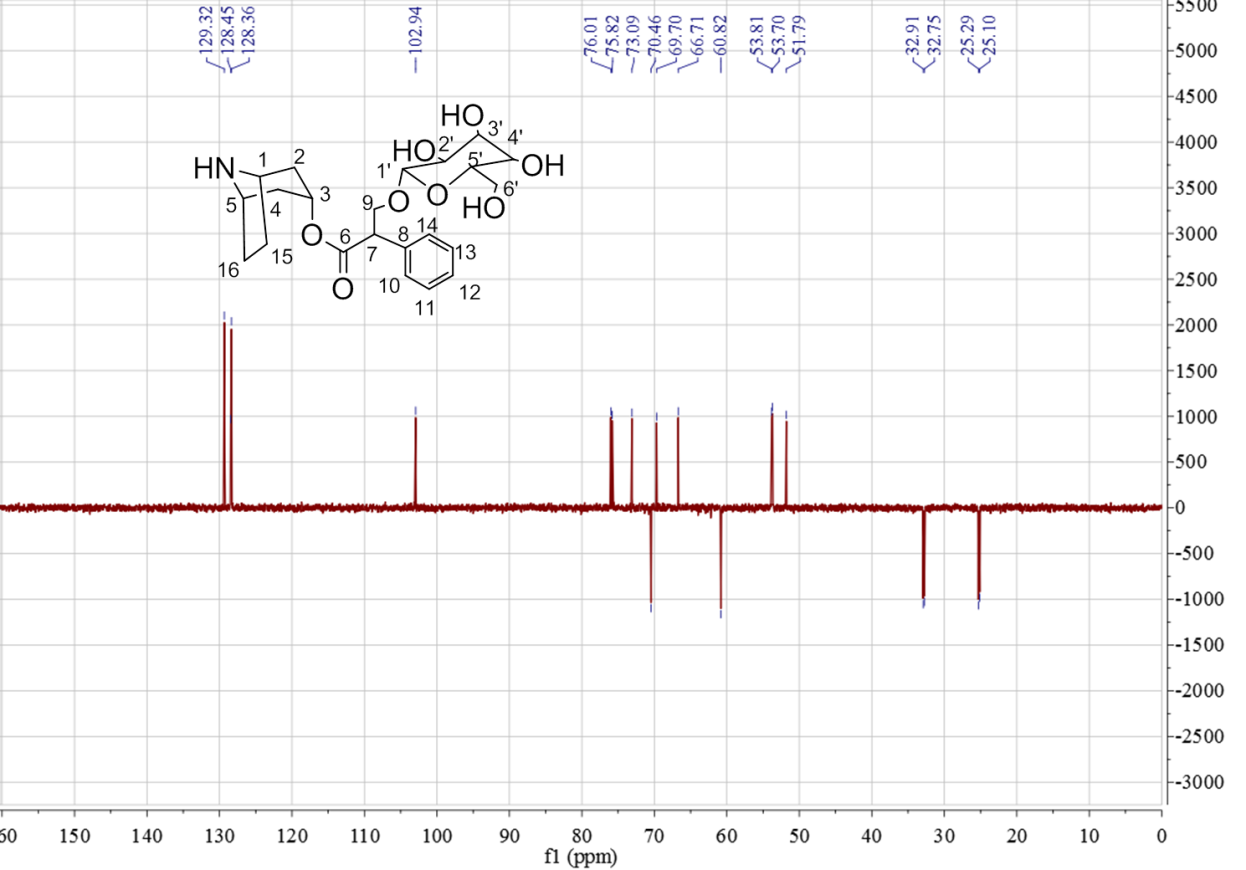


Fig. S31 DEPT 135 spectrum of **12** in D_2_O.


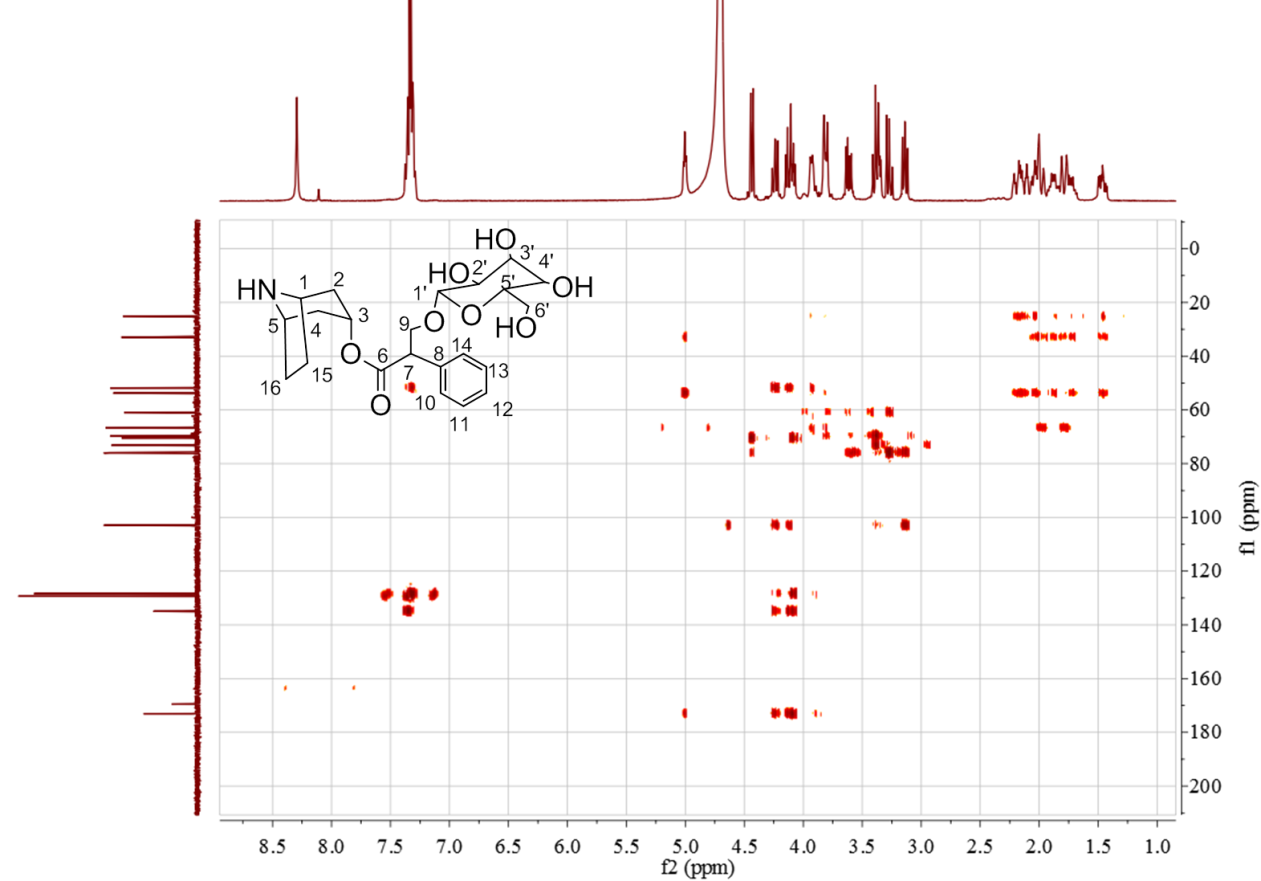


Fig. S32 HMBC spectrum of **12** in D_2_O.


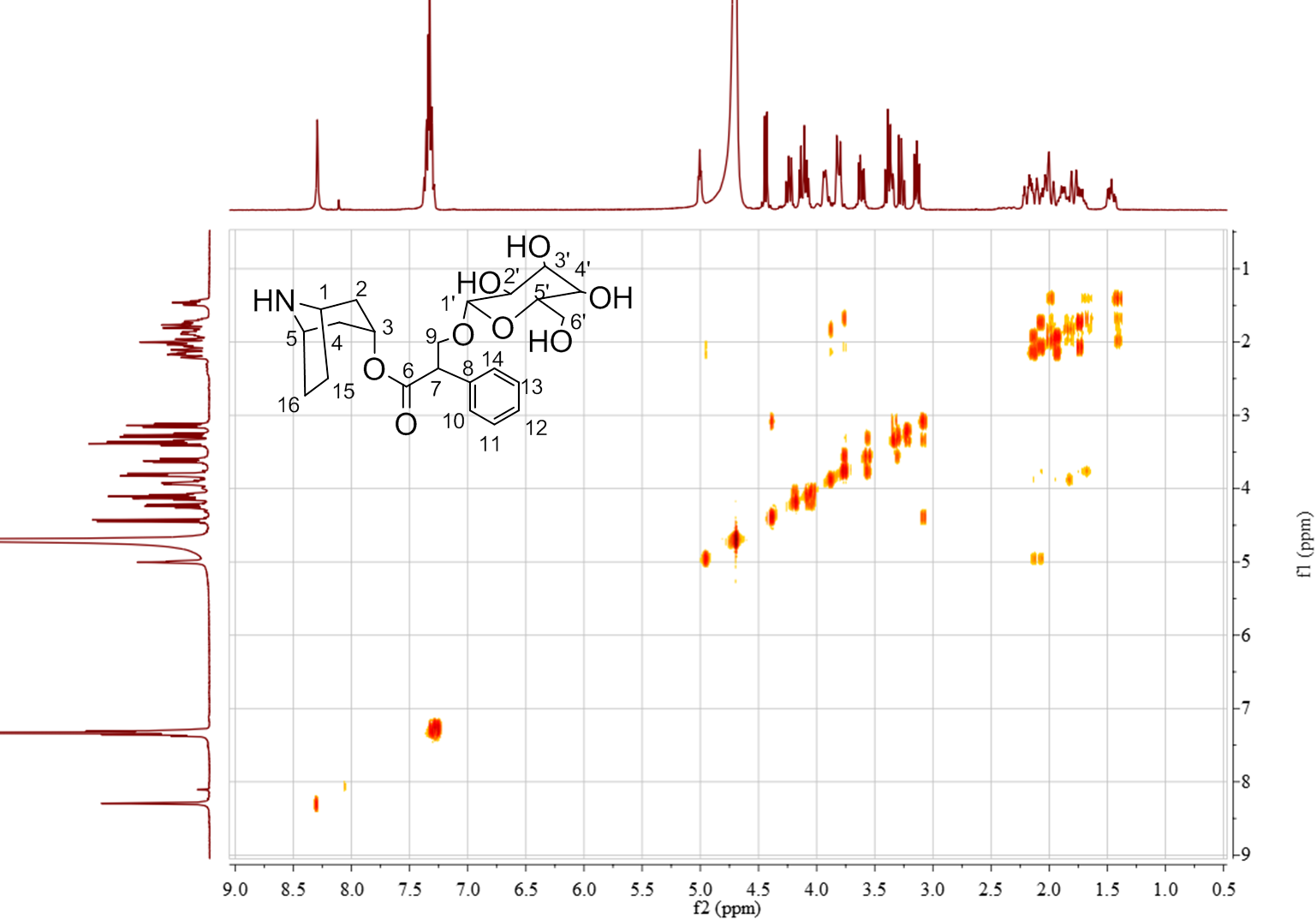


Fig. S33 H,H-COSY spectrum of **12** in D_2_O.


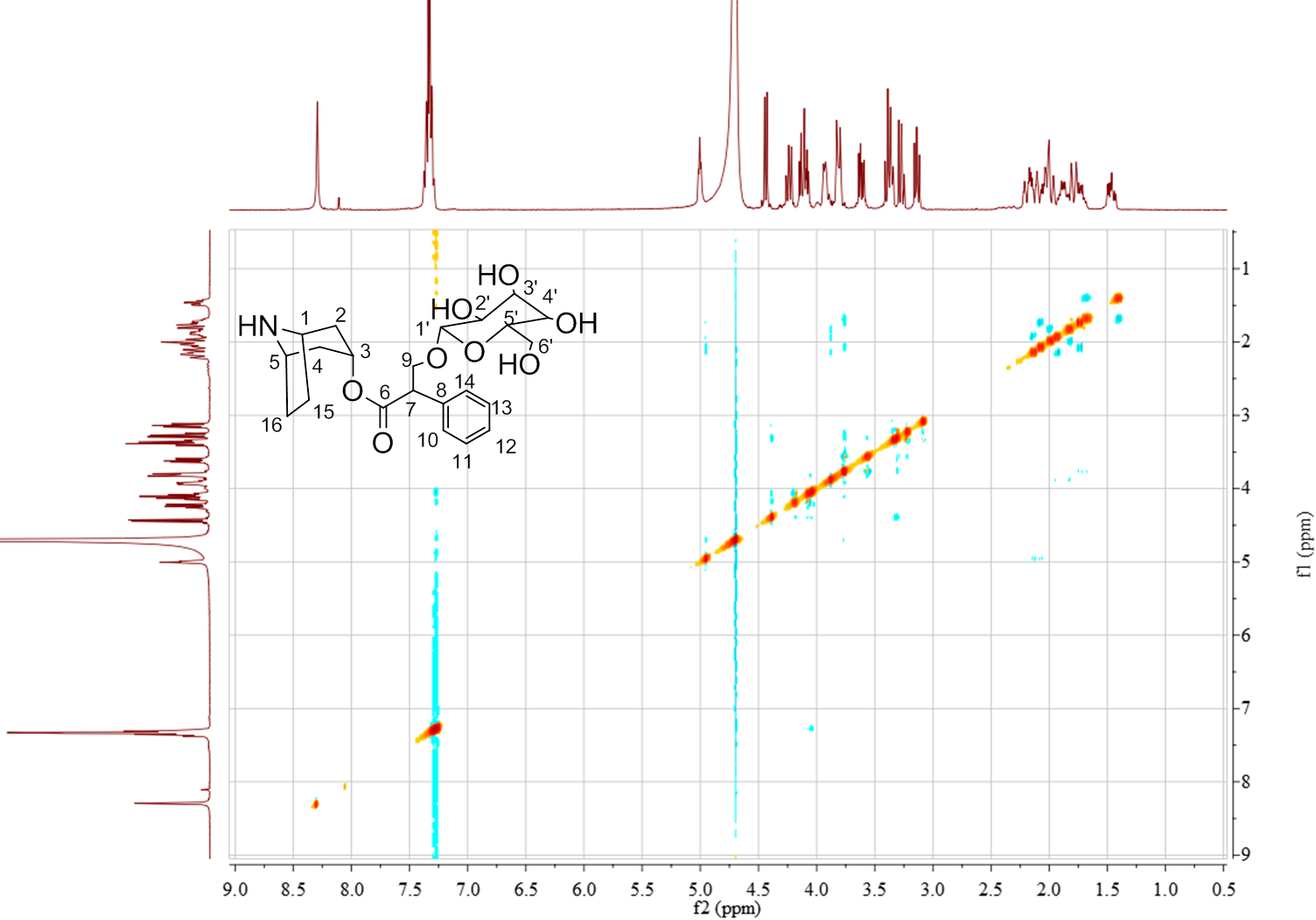


Fig. S34 NOESY spectrum of **12** in D_2_O.

Compound **13** was isolated as a white power. The purity and molecular formula were identified in HPLC-ESI-MS. The m/z in positive mode was 235.1445 and molecular formula was C_13_H_18_N_2_O_2_. The predominant fragment ions were 235.1434, 218.1145 and 147.0411, which was in consistent with N-trans-p-Coumaroylputrescine^4^. In the ^1^H NMR spectrum, protons of 7.32 and 6.42 and coupling constant between them (*J* = 15.7 Hz) characterized the presence of C=C in transform. Symmetric hydrogens including 7.39 (2H) and 6.80 (2H) confirmed the 1,4- disubstituted benzene. Thus, this compound was identified as N-trans-p-Coumaroylputrescine.

^1^H NMR (400 MHz, DMSO) δ 7.39 (d, 2H, 3-H, 5-H), 7.32 (d, *J* = 15.7 Hz, 1H, 7-H), 6.80 (d, *J* = 8.5 Hz, 2H, 2-H, 6-H), 6.42 (d, *J* = 15.7 Hz, 1H, 8-H), 3.18 (t, *J* = 5.5 Hz, 2H, 11-H, 11-H), 2.79 (t, *J* = 7.1 Hz, 2H, 14-H, 14-H), 1.66 – 1.38 (m, 4H, 12-H, 12-H, 13-H, 13-H).

Compound **14** was isolated as a white power. The purity and molecular formula were identified in HPLC-ESI-MS. The m/z in positive mode was 251.1 and molecular formula was C_13_H_18_N_2_O_3_. The predominant fragment ions were 251.1395 and 163.0389, which was in consistent with N-caffeoylputrescine^5^. In the ^1^H NMR spectrum, protons of 7.21 and 6.37 and coupling constant between them (*J* = 15.5 Hz) characterized the presence of C=C in transform. Three hydrogens including 7.08 (s), 6.79 and 6.70 confirmed the 1,2,4- trisubstituted benzene. Thus, this compound was identified as N-caffeoylputrescine.

^1^H NMR (400 MHz, DMSO) δ 7.21 (d, *J* = 15.7 Hz, 1H, 7-H), 7.08 (s, 1H, 3-H), 6.79 (d, *J* = 8.1 Hz, 1H, 5-H), 6.74 – 6.67 (d, *J* = 8.1 Hz, 1H, 6-H), 6.37 (d, *J* = 15.5 Hz, 1H, 8-H), 3.17 (m, 2H, 11-H, 11-H), 2.77 (m, 2H, 14-H, 14-H), 1.53 (m, 4H, 12-H, 12-H, 13-H, 13-H).

Compound **15**

Compound **15** was isolated as a white power. The purity and molecular formula were identified in HPLC-ESI-MS. The m/z in positive mode was 265.1669 and molecular formula was C_14_H_20_N_2_O_3_. The main fragment ions were 265.1669, 248.1280 and 177.0575, resulted by neutral loss of NH_3_ and putrescine molecule respectively. The ^1^H NMR spectrum are of high similarity to that of **14**, except the addition of methyl at OCH_3_. Thus, this compound was identified as N-trans-isoferuloylputrescine^6,7^.

^1^H NMR (400 MHz, DMSO) δ 7.29 (d, *J* = 15.7 Hz, 1H, 7-H), 7.14 (d, *J* = 1.5 Hz, 1H, 3-H), 6.98 (dd, *J* = 8.2, 1.6 Hz, 1H, 5-H), 6.79 (d, *J* = 8.1 Hz, 1H, 6-H), 6.54 (d, *J* = 15.7 Hz, 1H, 8-H), 3.79 (s, 3H, 15-H), 3.17 (dd, *J* = 11.8, 6.0 Hz, 2H, 11-H, 11-H), 2.75 (d, *J* = 6.8 Hz, 2H, 14-H, 14-H), 1.60 (m, 2H, 13-H, 13-H), 1.56 – 1.44 (m, 2H, 12-H, 12-H).

^13^C NMR (400 MHz, DMSO) δ 165.46 (C-9), 148.24 (C-1), 147.80 (C-2), 138.57 (C-7), 126.45 (C-4), 121.57 (C-5), 119.34 (C-8), 115.59 (C-6), 110.77 (C-3), 55.59 (C-15), 38.39 (C-14), 37.89 (C-11), 25.96 (C-12), 24.71 (C-13).

Compound **16**

Compound **16** was isolated as a white power. The purity and molecular formula were identified in HPLC-ESI-MS. The m/z in positive mode was 265.1757 and molecular formula was C_14_H_20_N_2_O_3_. The main fragment ions were 265.1757 and 177.0621, resulted by cleavage of amide bond. The ^1^H NMR spectrum ar.e of high similarity to that of **15**, except the C=C in cis-form (*J* = 12.9 Hz). Thus, this compound was identified as N-cis-isoferuloylputrescine^7^.

^1^H NMR (400 MHz, DMSO) δ 7.71 (d, J = 1.7 Hz, 1H, 3-H), 7.10 (dd, J = 8.3, 1.7 Hz, 1H, 5-H), 6.75 (d, J = 8.2 Hz, 1H, 6-H), 6.49 (d, J = 13.0 Hz, 1H, 7-H), 5.78 (d, J = 12.9 Hz, 1H, 8-H), 3.74 (s, 3H, 15-H), 3.12 (dd, J = 12.3, 6.3 Hz, 2H, 11-H, 11-H), 2.75 (t, J = 6.8 Hz, 2H, 14-H, 14-H), 1.62 – 1.39 (m, 4H, 12-H, 12-H, 13-H, 13-H).

^13^C NMR (400 MHz, DMSO) δ 166.34 (C-9), 148.17 (C-1), 147.35 (C-2), 137.33 (C-7), 127.06 (C-4), 124.80 (C-5), 121.31 (C-8), 115.38 (C-6), 114.85 (C-3), 38.90 (C-15), 38.40 (C-11), 26.50 (C-12), 25.41 (C-13).

Compound **17**

Compound **17** was isolated as a white power. The purity and molecular formula were identified in HPLC-ESI-MS. The m/z in positive mode was 474.2608 and molecular formula was C_25_H_35_N_3_O_6_. Based on the consistence of formula and 1H NMR spectrum, it was identified as N^1^,N^10^-di-dihydrocaffeoylspermidine^8^. The detailed NMR information was below.

^1^H NMR (400 MHz, D_2_O) δ 6.74 (d, *J* = 8.0 Hz, 2H, 3-H, 24-H), 6.66 (d, *J* = 7.2 Hz, 2H, 5-H, 28-H), 6.59 (d, *J* = 7.9 Hz, 2H, 6-H, 27-H), 3.08 (t, *J* = 6.1 Hz, 2H, 11-H, 11-H), 2.98 (s, 2H, 18-H, 18-H), 2.71 (dd, *J* = 12.9, 6.3 Hz, 4H, 13-H, 13-H, 15-H, 15-H), 2.65 (s, 2H, 7-H, 7-H), 2.42 (m, 6H, 8-H, 8-H, 21-H, 21-H, 22-H, 22-H), 1.64 – 1.51 (m, 2H, 12-H, 12-H), 1.23 (m, 4H, 16-H, 16-H, 17-H, 17-H).

Compound **18**

Compound **18** was isolated as a white power. UV (H_2_O) λmax (Abs.): 192.5 (0.6851), 217.5 (0.1485), 278.5 (0.0418) nm; IR (KBr) νmax: 3425 (-OH), 1633 (-CONH-). The purity and molecular formula were identified in HPLC-ESI-MS. The m/z in positive mode was 458.2647 and molecular formula was C_25_H_35_N_3_O_5_. It is a new compound named N1-p-dihydrocoumaroyl -N10-dihydrocaffeoyl spermidine.

^1^H NMR (400 MHz, D_2_O) δ 7.04 (d, *J* = 8.4 Hz, 2H, 3-H, 5-H), 6.74 (dd, *J* = 8.3, 2.4 Hz, 3H, 27-H, 2-H, 6-H), 6.65 (d, *J* = 1.8 Hz, 1H, 24-H), 6.58 (dd, *J* = 8.1, 1.8 Hz, 1H, 28-H), 3.07 (t, *J* = 6.3 Hz, 2H, 11-H, 11-H), 2.98 (s, 2H, 18-H, 18-H), 2.76 (t, *J* = 6.9 Hz, 2H, 7-H, 7-H), 2.70 (t, *J* = 6.9 Hz, 2H, 22-H, 22-H), 2.65 (s, 2H, 15-H, 15-H), 2.50 – 2.35 (m, 6H, 8-H, 8-H, 13-H, 13-H, 21-H, 21-H), 1.62 – 1.53 (m, 2H, 12-H, 12-H), 1.23 (d, *J* = 3.1 Hz, 4H, 16-H, 16-H, 17-H, 17-H).

^13^C NMR (500 MHz, D_2_O) δ 176.33 (C-9), 175.73 (C-20), 153.93 (C-1), 143.84 (C-25), 142.24 (C-26), 133.32 (C-23), 132.25 (C-4), 129.88 (C-3,C-5), 120.92 (C-28), 116.42 (C-24), 116.32 (C-27), 115.42 (C-2, C-6), 47.25 (C-15), 44.69 (C-8), 38.31 (C-18), 37.69 (C-21), 37.13 (C-13), 35.74 (C-11), 30.74 (C-22), 30.32 (C-7), 25.56 (C-17), 25.46 (C-12), 22.80 (C-16).


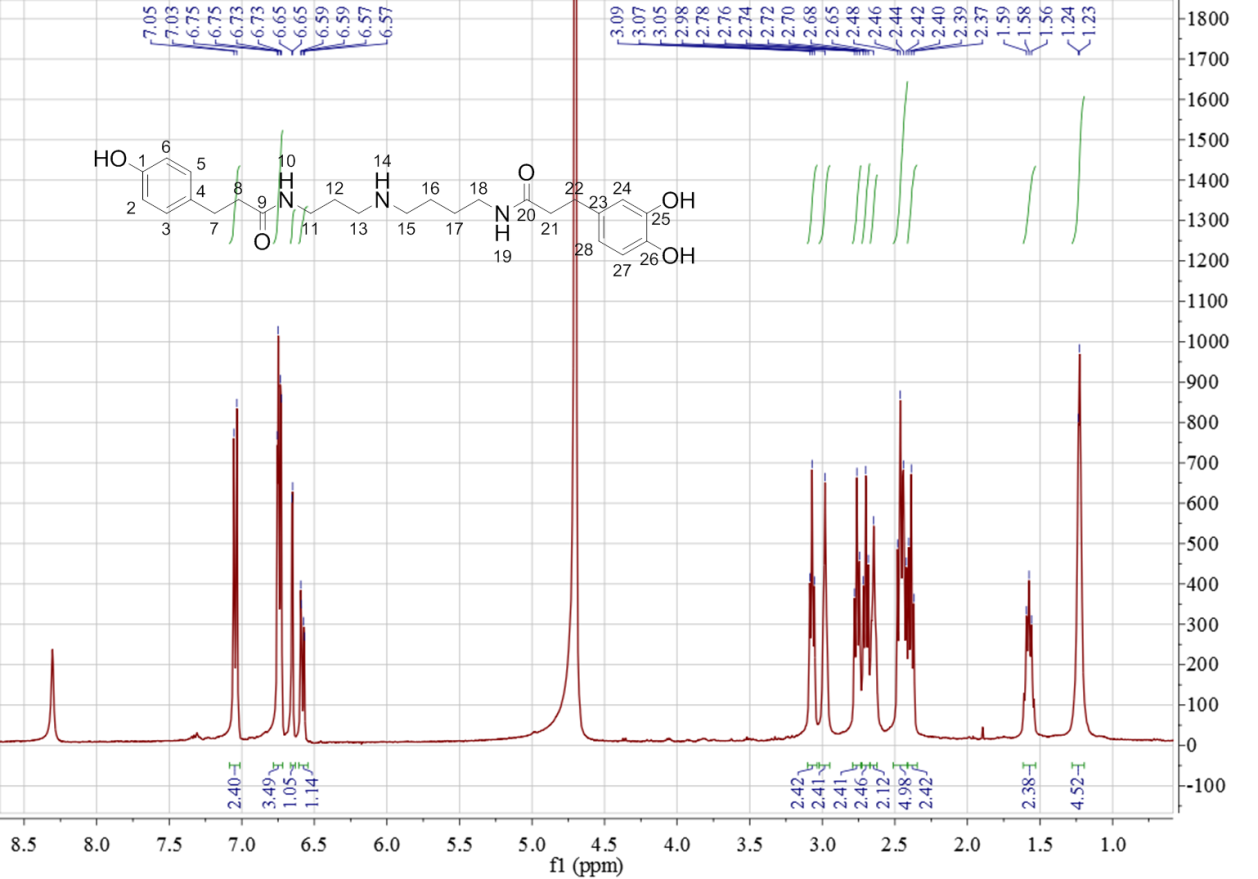


Fig. S35 ^1^H spectrum of **18** in D_2_O.


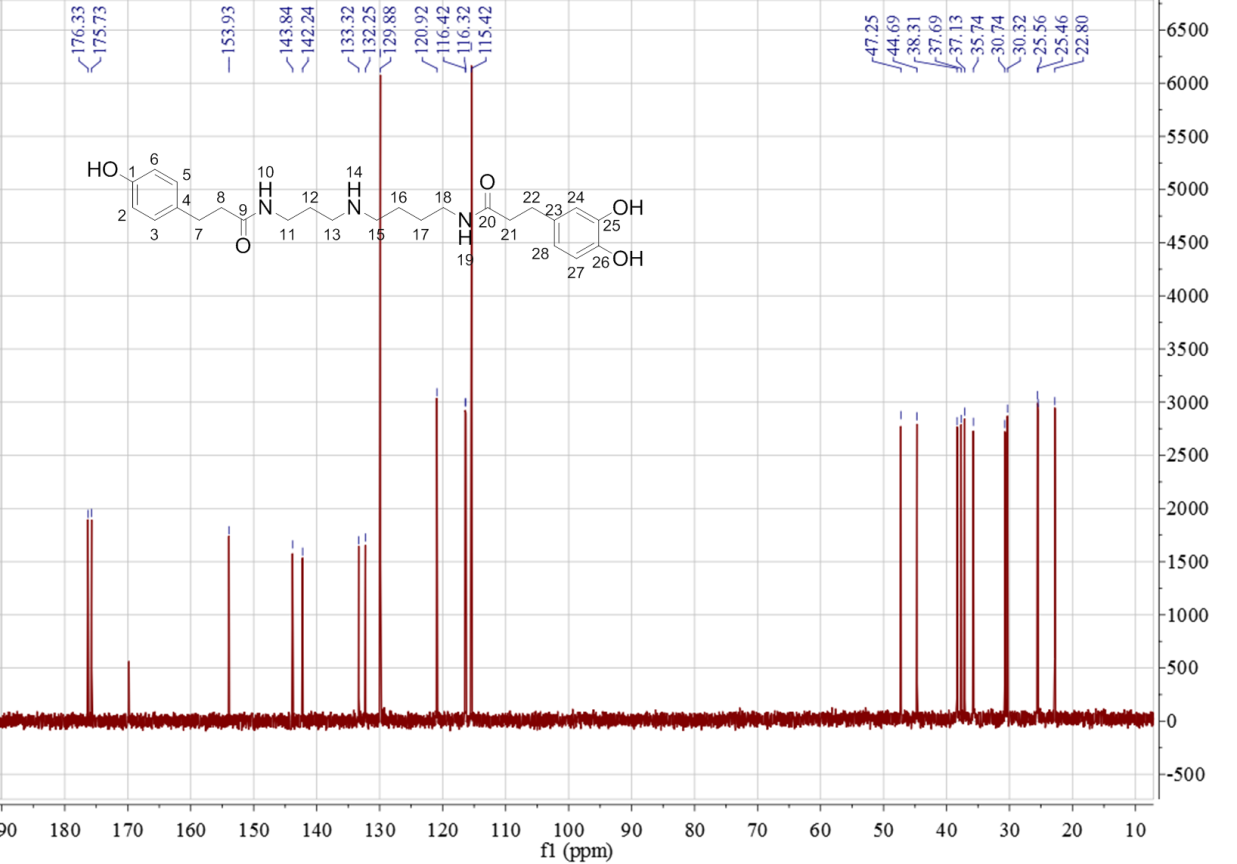


Fig. S36 ^13^C spectrum of **18** in D_2_O.


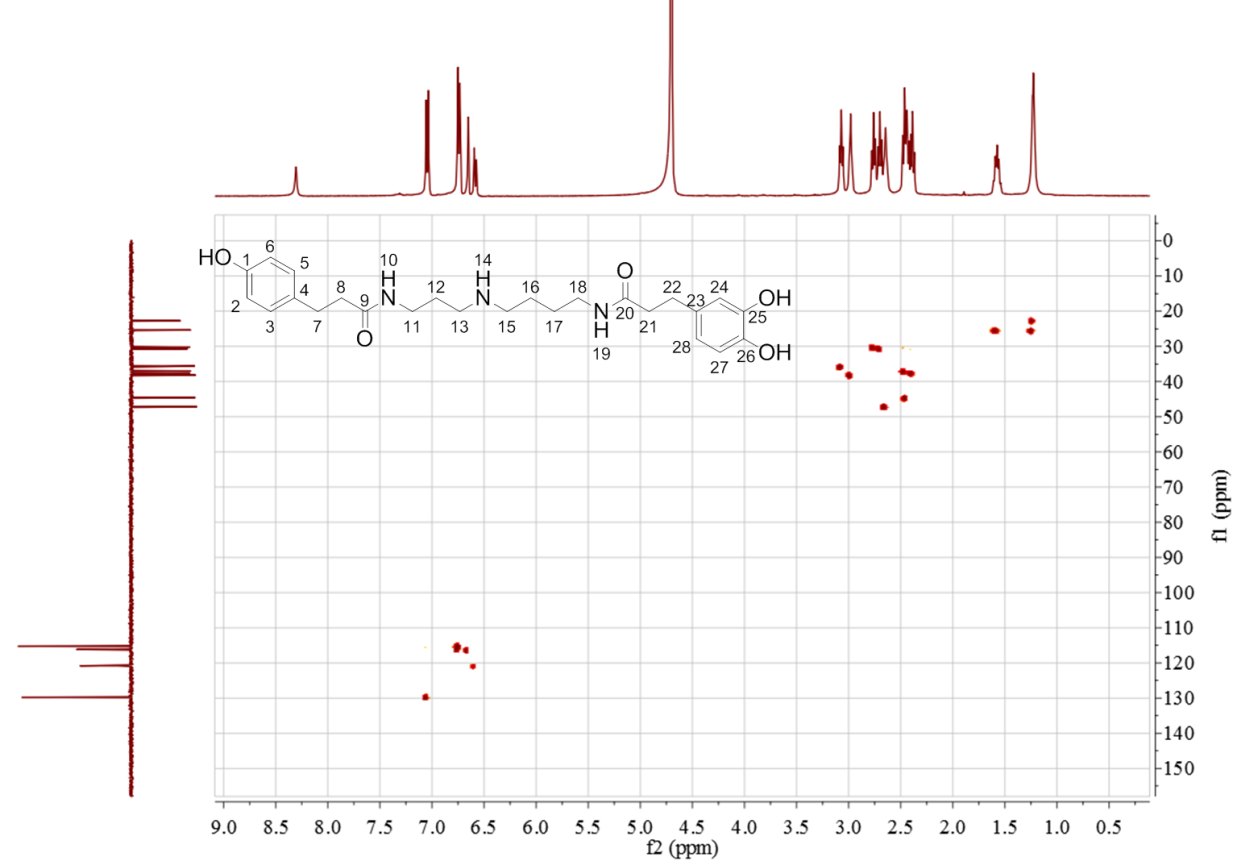


Fig. S37 HSQC spectrum of **18** in D_2_O.


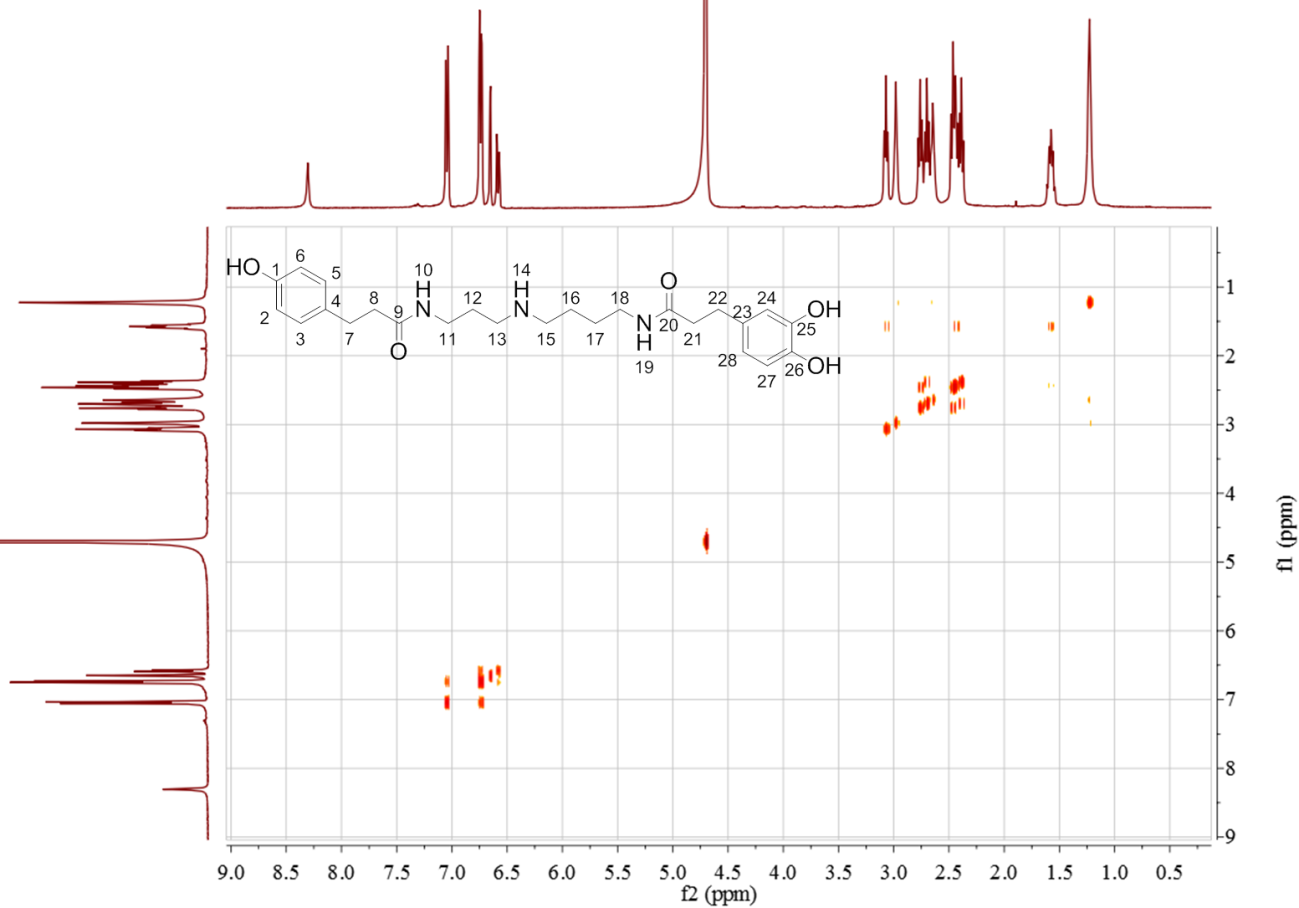


Fig. S38H,H-COSY spectrum of **18** in D_2_O.


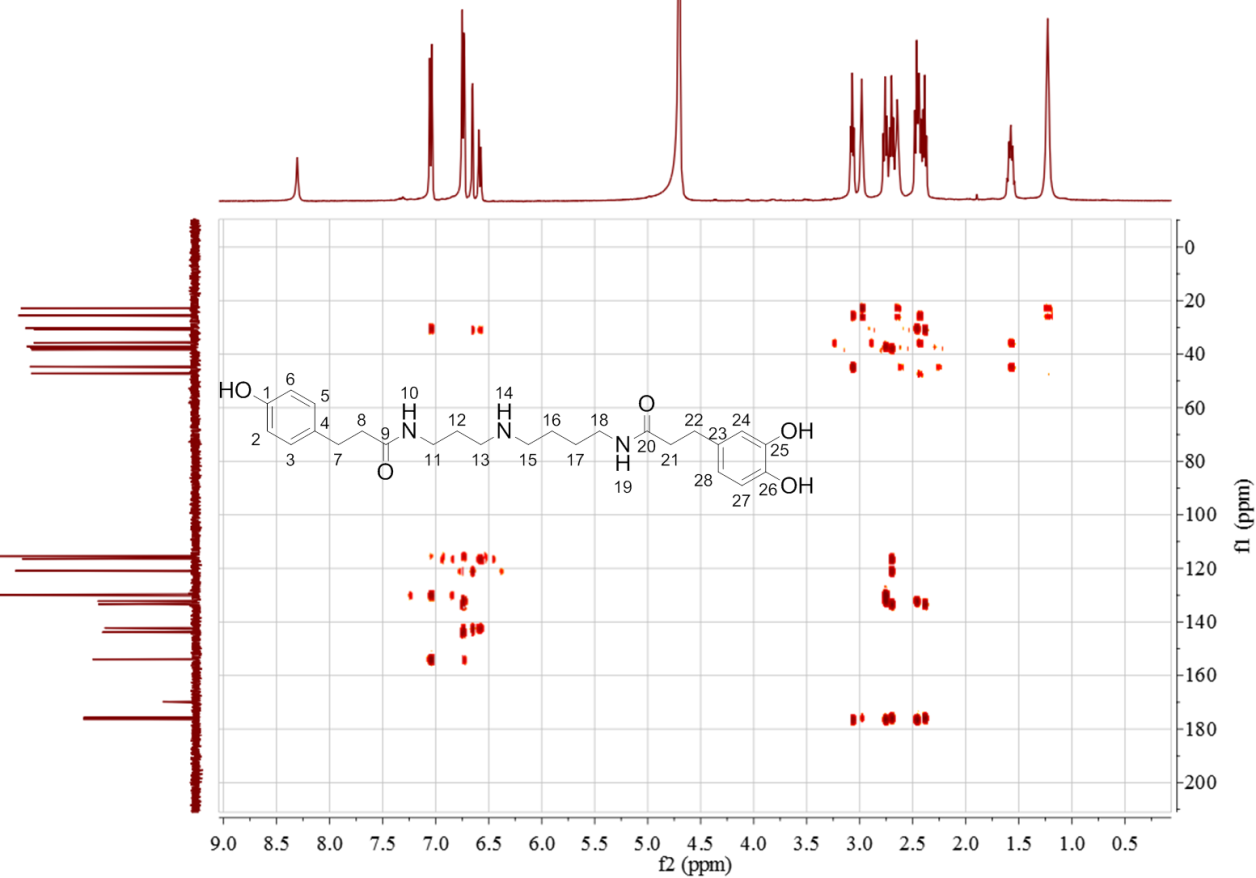


Fig. S39 HMBC spectrum of **18** in D_2_O.

Compound **19** was isolated as a white power. The purity and molecular formula were identified in HPLC-ESI-MS. The m/z in positive mode was 472.2447 and molecular formula was C_25_H_33_N_3_O_6_. Based on the consistence of formula and 1H NMR spectrum, this compound was confirmed to be Scotanamine D^9^. The detailed NMR information was as follows.

^1^H NMR (400 MHz, D_2_O) δ 7.24 (d, *J* = 15.8 Hz, 1H, 7-H), 6.99 (s, 1H, 3-H), 6.92 (d, *J* = 8.3 Hz, 1H, 6-H), 6.79 (d, *J* = 8.2 Hz, 1H, 5-H), 6.70 (d, *J* = 8.1 Hz, 1H, 27-H), 6.60 (d, *J* = 1.9 Hz, 1H, 24-H), 6.51 (dd, *J* = 8.1, 1.8 Hz, 1H, 28-H), 6.26 (d, *J* = 15.8 Hz, 1H, 8-H), 3.27 (t, *J* = 6.5 Hz, 2H, 11-H, 11-H), 2.96 (t, *J* = 5.7 Hz, 2H, 18-H, 18-H), 2.93 – 2.85 (m, 2H, 13-H, 13-H), 2.80 (t, *J* = 6.9 Hz, 2H, 15-H, 15-H), 2.64 (t, *J* = 6.9 Hz, 2H, 22-H, 22-H), 2.32 (t, *J* = 7.0 Hz, 2H, 21-H, 21-H), 1.88 – 1.76 (m, 2H, 12-H, 12-H), 1.33 – 1.18 (m, 4H, 16-H, 16-H, 17-H, 17-H).

Compound **20** was isolated as a white power. The purity and molecular formula were identified in HPLC-ESI-MS. The m/z in positive mode was 472.2435 and molecular formula was C_25_H_33_N_3_O_6_. Based on the consistence of formula and 1H NMR spectrum, this compound was confirmed to be N1-caffeoyl-N3-dihydrocaffeoyl spermidine^10^. The detailed NMR information was as follows.

^1^H NMR (600 MHz, DMSO) δ 7.24 (d, *J* = 15.6 Hz, 1H, 22-H), 6.98 (s, 1H, 24-H), 6.82 (d, *J* = 7.8 Hz, 1H, 27-H), 6.75 (d, *J* = 8.0 Hz, 1H, 6-H), 6.62 (d, *J* = 7.9 Hz, 1H, 5-H), 6.59 (s, 1H, 3-H), 6.42 (d, *J* = 8.4 Hz, 1H, 28-H), 6.35 (d, *J* = 15.4 Hz, 1H, 21-H), 3.19 (d, *J* = 7.2 Hz, 2H, 18-H, 18-H), 3.09 (s, 2H, 11-H, 11-H), 2.82 (s, 2H, 15-H, 15-H), 2.72 (s, 2H, 13-H, 13-H), 2.64 (t, *J* = 7.4 Hz, 2H, 7-H, 7-H), 2.30 (t, *J* = 7.4 Hz, 2H, 8-H, 8-H), 1.68 (m, 2H, 17-H, 17-H), 1.60 (s, 2H, 16-H, 16-H), 1.50 (m, 2H, 12-H, 12-H).


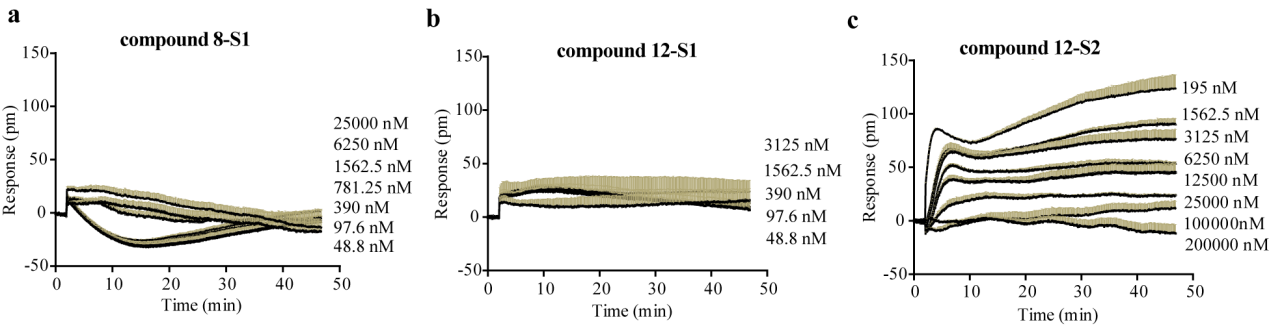


Fig. S40 Label-free pharmacological profiling of compound **8** and **12** on M3 receptor in HT-29 cells. (a) Real-time DMR response of **8** at different concentrations; (b) Real-time DMR response of compound **12** at different doses; (c) Real-time DMR response of 16µM acetylcholine after pretreatment with compound **12** at different doses.

**References**

1 Chen, Y., Chen, H. X., Du, P., Han, F. M. & Zhang, H. S. Analysis of anisodine and identification of twenty of its metabolites in rat urine by liquid chromatography-tandem mass spectrometry. *Chromatographia* **62**, 563-569 (2005).

2 Chen, H.-X., Du, P., Han, F.-M. & Chen, Y. Detection of anisodamine and its metabolites in rat feces by tandem mass spectrometry. *Acta pharmaceutica Sinica* **41**, 1166-1169 (2006).

3 Jousse, C. *et al.* Tropane alkaloid profiling of hydroponic Datura innoxia Mill. Plants inoculated with Agrobacterium rhizogenes. *Phytochem. Anal.* **21**, 118-127 (2010).

4 Sun, J. *et al.* Characterization and quantitative analysis of phenylpropanoid amides in eggplant (Solanum melongena L.) by high performance liquid chromatography coupled with diode array detection and hybrid ion trap time-of-flight mass spectrometry. *J. Agric. Food Chem.* **63**, 3426-3436 (2015).

5 Voynikov, Y. *et al.* Hydroxycinnamic acid amide profile of Solanum schimperianum Hochst by UPLC-HRMS. *Int. J. Mass Spectrom.* **408**, 42-50 (2016).

6 Dastmalchi, K. *et al.* Solving the jigsaw puzzle of wound-healing potato cultivars: metabolite profiling and antioxidant activity of polar extracts. *J. Agric. Food Chem.* **62**, 7963-7975 (2014).

7 Xu, S., Zhao, X., Li, R. & Liu, H. Theoretical study on isomerization of (E) -N-(4-aminobutyl) -3-(3-hydroxy-4- methoxyphenyl) acrylamide. *Computers and Applied Chemistry* **24**, 899-902 (2007).

8 Yinglyongnarongkul, B.-e., Apiratikul, N., Aroonrerk, N. & Suksamrarn, A. Synthesis of bis, tris and tetra(dihydrocaffeoyl)polyamine conjugates as antibacterial agents against VRSA. *Arch. Pharm. Res.* **31**, 698-704 (2008).

9 Long, Z. *et al.* Amide alkaloids from Scopolia tangutica. *Planta Med.* **80**, 1124-1130 (2014).

10 Heinrich, M. *et al.* High levels of jasmonic acid antagonize the biosynthesis of gibberellins and inhibit the growth of Nicotiana attenuata stems. *Plant J* **73**, 591-606 (2013).
